# Characteristics of Children Prescribed Antipsychotics: Analysis of Routinely Collected Data

**DOI:** 10.1089/cap.2017.0003

**Published:** 2018-04-01

**Authors:** Sinead Brophy, Jonathan Kennedy, Fabiola Fernandez-Gutierrez, Ann John, Robert Potter, Christine Linehan, Michael Kerr

**Affiliations:** ^1^ FARR Institute (CIPHER), Swansea University Medical School, Swansea, United Kingdom.; ^2^ Swansea University Medical School, Swansea, United Kingdom.; ^3^ Population Psychiatry, Suicide and Informatics, FARR Institute (CIPHER), Swansea University Medical School, Swansea, United Kingdom.; ^4^ Cwm Taf Health University Health Board and Institute of Psychological Medicine and Clinical Neuroscience, Cardiff University School of Medicine, Cardiff, Wales.; ^5^ UCD Centre for Disability Studies, University College Dublin, Dublin, Ireland.; ^6^ Institute of Psychological Medicine and Clinical Neuroscience, Cardiff University School of Medicine, Cardiff, Wales.

**Keywords:** antipsychotic, routine data, intellectual difficulty, prior event rate ratio

## Abstract

***Objective:*** Antipsychotics are licensed for psychosis and are also prescribed for behavior control. This study aims to examine characteristics and outcomes of children prescribed antipsychotics.

***Methods:*** A cohort study using general practice and hospital records linked with education records for 1,488,936 children living in Wales between 1999 and 2015. The characteristics of the children who were prescribed antipsychotics are presented using descriptive statistics and outcomes such as respiratory illness, diabetes, and injury were analyzed using multilevel logistic regression and the prior event rate ratio (PERR).

***Results:*** Children with intellectual difficulty/autism were more likely to be prescribed antipsychotics (2.8% have been prescribed an antipsychotic [75% with autism] compared with 0.15% of children without intellectual difficulty). Those with intellectual disabilities/autism were prescribed antipsychotics at a younger age and for a longer period. Antipsychotic use was associated with a higher rate of respiratory illness for all (PERR of hospital admission: 1.55 [95% CI: 1.51–1.598] or increase in rate of 2 per 100 per year in those treated), and for those with intellectual difficulty/autism, there was a higher rate of injury and hospitalized depression. However, among those without intellectual difficulty/autism, there were lower rates of depression (PERR: 0.55 [95% CI: 0.51–0.59]).

***Conclusions:*** This work shows real-world use of antipsychotics and provides information on the rate of possible adverse events in children treated. Antipsychotics are predominantly used for those with intellectual difficulty/autism rather than those with a psychotic diagnosis. There is evidence that rates of respiratory disease, epilepsy, and diabetes are also higher postantipsychotic use for all. In those with intellectual difficulty/autism, hospital-admitted depression and injury are higher postantipsychotic use. The use of antipsychotics for behavioral management is likely to have increased cost implications to the healthcare system.

## Introduction

Antipsychotic medication such as haloperidol, aripiprazole (for schizophrenia, bipolar [mania]), or risperidone (for conduct disorders) are licensed in the United Kingdom for use in children (Kavanagh et al. 2015). Off-label (not licensed for children and adolescents) olanzapine, quetiapine, and amisulpride are also used (Kavanagh et al. 2015) and the majority of antipsychotic prescriptions is off label (Carton et al. 2015). Side effects of modern (atypical) antipsychotics include weight gain, diabetes, high cholesterol, and cardiovascular disease, and they are also associated with an increase in seizure risk (Alper et al. 2007). Long-term use can in rare cases be associated with tardive dyskinesia—a neurological disorder resulting in compulsive movement. Despite the side effects of antipsychotics, their use does appear to be on the rise (Cooper et al. 2004; Olfson et al. 2006) and recent studies suggest that they are prescribed more often for those with intellectual difficulty especially those with autism (Sheehan et al. 2015). Yet, people with intellectual difficulty do not have significantly higher rates of psychosis compared with the general population but are likely to have more aggressive or disruptive behavior. Many of the antipsychotics have sedative properties and some are licensed for rapid tranquilization to manage acute violent behavior. However, there are “deep concerns” about the overuse of antipsychotic medicines in people with intellectual difficulty and autism (Health 2012) in terms of the extent to which these drugs are used outside licensed indications with the aim of managing behavior problems. In addition, the duration of treatment of the antipsychotic medications has been increasing (Kalverdijk et al. 2008; Rani et al. 2008; Tyrer and Kendall 2009).

There is some “limited evidence” that antipsychotics (e.g., risperidone) can reduce aggression and conduct problems in children aged 5 to 18 with disruptive behavior in the short term (4–10 weeks) from a small number of studies in which there was some risk of bias of overestimating the true intervention effect (Loy et al. 2012). NICE Conduct Disorder guidelines (NICE, 2013) recommend that clinicians should not offer pharmacological intervention for the routine management of behavioral problems but suggest risperidone may be considered in some cases for the short-term management of young people with explosive anger and severe emotion dysregulation. In terms of autism, trials show improved behavioral problems, but also higher levels of adverse events such as somnolence, upper respiratory infection (Pandina et al. 2007), and higher weight gain (Scahill et al. 2016; McCracken et al. 2002) Therefore, the evidence base for behavioral management using antipsychotics is still at an early stage and there are few studies examining the long-term effects of this medication when started in childhood. For example, the most recent long-term study has found that antipsychotic use has increased for adolescents and young adults especially among boys, but this study did not look at long-term outcomes associated with these medications (Olfson et al. 2015).

### Aims

This population-based observational study aimed to examine the characteristics of children prescribed antipsychotics as found in general practice (GP) records, before the age of 18, with a special focus on those with intellectual difficulty and/or autism to examine how antipsychotics are prescribed in a real-world setting and evidence of long-term effects.

## Methods

### Study design and setting

A cohort study using routinely collected linked data, including general practitioner, hospital admission, and education data. All children in Wales between 1999 and 2015 were selected and stratified by intellectual difficulty/autism and antipsychotic use.

### Data sources

The Farr Institute of Health Informatics Research comprises four nodes distributed across the United Kingdom. One of the nodes, CIPHER (Centre for Improvement in Population Health through E-records), brings together routine health data using the Secure Anonymised Information Linkage (SAIL) data bank (Ford et al. 2009; Lyons et al. 2014), which anonymously links a wide range of person-based data using a unique personal identifier. For the purpose of this study, the GP records were linked with education data (DECELLS) and hospital admission (inpatient) data. The GP system uses READ codes, which are 5-digit codes that relate to diagnosis, medication, and process of care codes. This study examines GP-prescribed prescriptions (but not hospital prescribing) for 70% of all GP in Wales between 1999 and 2015. The secondary care inpatient data set uses ICD10 codes for diagnosis and surgical interventions, and the education data set contains information regarding special needs status (SNS), key stage achievements, and attendance, and we used the information on SNS in this study. These records were linked at the individual level for all children in Wales and then stratified for subanalysis. Quality of linkage has been assessed and reported as 99.9% for GP records and 99.3% for hospital records (Lyons et al. 2009). All linkage was at the person level. The requirement that a participant needed to have GP who was submitting records to the SAIL data bank meant that this study contained 70% of all children living in Wales between 1999 and 2015.

### Ethical approval

The data held by HIRU in the SAIL system are anonymized and have been obtained with the permission of the relevant Caldicott Guardian/Data Protection Officer; therefore, the National Research Ethics Service has stated that no ethical review is required. Approval was obtained from the SAIL Information Governance Review Panel to use the SAIL system for this research question.

### Participants and diagnosis of interest

Children with intellectual difficulty/autism were identified and flagged as any child who had codes in the GP data for intellectual difficulty health assessment, mental retardation, Down's syndrome, learning disability, autism (excluding Asperger's syndrome), or any child who had codes in the hospital admission for chromosome disorders associated with intellectual disorder, Down's syndrome, mental retardation, or autism (excluding Asperger's syndrome), or any child in the education database who was coded as profound and multiple learning disability, autistic spectrum disorders, severe learning disability, or in the congenital abnormalities data set of mental retardation, pervasive developmental disorders, or Down's syndrome (See Supplementary Table S1 for codes; Supplementary Data are available online at www.liebertpub.com/cap).

The use of antipsychotic medication was identified to include first- and second-generation agents (see Supplementary Table S1 for codes). However, we did not include antidepressants, nonantipsychotic mood stabilizers, anxiolytics, and hypnotics (including benzodiazepines), antidementia drugs, or drugs for attention-deficit/hyperactivity disorder.

#### Variables

Descriptive variables for mental health conditions such as bipolar disorder, schizophrenia, psychosis, and potential outcomes such as diabetes, injury, and seizures were identified (see Supplementary Table S1 for codes. Injury codes are available on request) from GP and hospital admission data.

### Statistical analysis

All data were analyzed using STATA 13 to present demographic and descriptive data of groups stratified by intellectual difficulty/autism and by antipsychotic use.

Logistic regression was used to examine the influence of known confounders on the odds of having diabetes or epileptic seizure or injury code during a 12-month period when prescribed an antipsychotic drug compared with 12-month periods when not on the drug, clustered by individual, thus creating a multilevel or hierarchical model. For this analysis, all full GP records for a 12-month period were recorded per individual with the explanatory variable being a flag for “prescribed” antipsychotic 0 or 1 in that year and the outcome variable being a flag for any diabetes event, epilepsy event, or injury event in the GP/hospital in that year. The flag of 0 (no antipsychotic prescription) was given for years before an antipsychotic prescription and for years when a person was no longer prescribed antipsychotics. Confounders examined were intellectual difficulty, gender, age, and aggression code. This method can adjust for known confounders and can give an estimate of association or the odds ratio for each confounder.

To adjust for unmeasured confounders (e.g., variables such as family characteristics, diet, comorbidities, or personality traits), the prior event rate ratio (PERR) was used. This method examines the rate of diabetes/epilepsy/injury before prescription of the drug and after prescription compared with a before and after a random date period in children not prescribed antipsychotics. The PERR adjustment method estimates the effects of exposure to antipsychotic medication on the likelihood of experiencing the adverse medical outcomes of diabetes, epilepsy, or injury. The time to first event over 3 years of follow-up was calculated from the date of first antipsychotic (follow-up for 3 years) and for the 3 years before the date of first antipsychotic. For those not given an antipsychotic, a random date was chosen to calculate time to first event before and after this random date. The PERR is calculated as follows:

\begin{align*}
 { \rm { PERR } } = \; { \frac { { \rm { Rate \;ratio \;during \;post \;period } } }  { { \rm { Rate \;ratio \;during \;prior \;period } } } } 
\end{align*}

Confidence intervals were obtained by bootstrapping. The method assumes that the confounding effects are constant across the prior and postexposure periods and that there is no confounder by treatment interactions (Uddin et al. 2015).

## Results

**FIG. 1.**
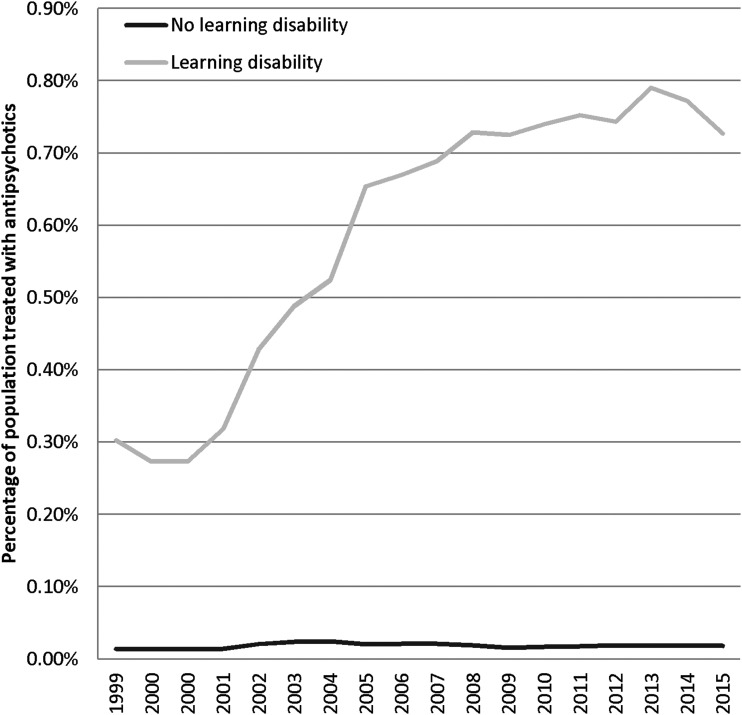
Percentage of child population treated with antipsychotics by year.

A total of 1,488,936 children younger than 18 years were identified from GP records (Table 1 and Supplementary Data S2) and stratified by (1) no intellectual difficulty/autism, no childhood history of antipsychotic use (*n* = 1,457,783, [97.9% of population], 51.4% male), (2) no intellectual difficulty/autism, antipsychotic use (*n* = 2204 [0.15% of population], 60.4% male), (3) intellectual difficulty, no childhood history of antipsychotic use (*n* = 28,125 [1.9% of population], 67% male), and (4) intellectual difficulty and history of antipsychotic use (*n* = 824 [0.04% of population], 78.0% male). Of those with an intellectual difficulty/autism, 2.84% (824/28,949) had antipsychotics compared with 0.15% (2204/1,459,987) who did not have an intellectual difficulty/autism (difference 2.7% [95% CI: 2.5–2.9]). In this cohort, 3028 children were prescribed an antipsychotic, 25% (*n* = 766) with intellectual difficulty/autism and no psychotic disorder code, 1.9% (*n* = 58) with intellectual difficulty/autism and psychotic disorder code, 11.8% (*n* = 357) with psychotic disorder code (and not intellectual difficulty), and 61.3% (*n* = 1847) with no intellectual difficulty/autism or psychotic disorder code (Table 1). The percentage treated with antipsychotics per year is seen in Figure 1.

**Table 1.** Comparison of Characteristics of Children Stratified by Intellectual Disability and Antipsychotic Use

	*Male gender*	*Attending special needs school*	*Deprived (lowest fifth at prescription of antibiotic)*	*Deprived (lowest fifth at diagnosis of ID/A)*
A. General population	749,194 (51.4%)	2166 (0.15%)	—	—
B. Antipsychotic	1331 (60.4%)	103 (4.6%)	658 (29.3%)	—
C. Intellectual difficulty/autism	18,880 (67.13%)	4806 (17.0%)	—	5479 (19.4%)
D. Intellectual difficulty/autism and antipsychotic	643 (78.0%)	402 (48.4%)	194 (23.35%)	177 (21.3%)
Difference between D and B (95% CI)	17.6% (14.1–21.3)	44.1% (40.6–47.6)	−6.3% (−2.8 to −9.7)	—
Difference between D and C (95% CI)	10.9% (7.9–13.7)	31.7% (28.3–35.1)	—	2.0% (−0.7 to 5.0)

	*Psychotic disorder*	*Aggression*	*Tourette*	*Autism*
A. General population	2463 (0.17%)	2968 (0.2%)	475 (0.03%)	—
B. Antipsychotic	357 (16.2%)	169 (7.5%)	161 (7.3%)	—
C. Intellectual difficulty/autism	103 (0.37%)	509 (1.8%)	102 (0.36%)	9559 (33.9%)
D. Intellectual difficulty/autism and antipsychotic	58 (7.0%)	148 (17.8%)	56 (6.8%)	620 (74.6%)
Difference between D and B (95% CI)	−9.16% (−6.7 to −11.4)	10.3% (7.6–13.3)	0.5% (−1.6 to 2.4)	—
Difference between D and C (95% CI)	6.7% (5.1–8.6)	16.2% (13.7–18.9)	6.4% (4.9–8.4)	41.3% (38.2–44.1)

	*ADHD*	*Downs*	*Epilepsy*	*Death in childhood*
			12,907 (0.9%)	
A. General population	1327 (0.09%)	—		17,931 (0.12%)
B. Antipsychotic	81 (3.6%)	—	96 (4.3%)	75 (3.3%)
C. Intellectual difficulty/autism	305 (1.1%)	745 (5.3%)	2185 (7.7%)	428 (1.5%)
D. Intellectual difficulty/autism and antipsychotic	41 (4.9%)	<5	162 (19.5%)	19 (2.3%)
Difference between D and B (95% CI)	1.3% (−0.25 to 3.1)	—	15.3% (12.6 to −18.3)	1.1% (−0.3 to 2.2)
Difference between D and C (95% CI)	3.9% (2.9–5.6)	2.0% (1.2–2.4)	11.9% (9.3–14.8)	0.8% (0–2.1)

	*Aged 0 to 7 at first prescription*	*Aged 8 to 14 at first prescription*	*Aged 15 to 18 at first prescription*	*Low prescription number (<3)*
A. General population	—	—	—	—
B. Antipsychotic	87 (3.9%)	572 (25.5%)	1584 (70.6%)	922 (41.1%)
C. Intellectual difficulty/autism	—	—	—	
D. Intellectual difficulty/autism and antipsychotic	40 (4.5%)	423 (50.9%)	368 (44.3%)	190 (22.9%)
Difference between D and B (95% CI)	0.9% (−0.7 to 2.8)	25.4% (21.5–29.2)	27.2% (23.3–31.0)	18.8% (15.2–22.2)
Difference between D and C (95% CI)	—	—	—	—

	*Prescription number 3 to 12*	*High prescription number (12+)*
A. General population	—	
B. Antipsychotic	759 (33.8%)	562 (25.1%)
C. Intellectual difficulty/autism	—	—
D. Intellectual difficulty/autism and antipsychotic	217(26.1%)	424 (51.0%)
Difference between D and B (95% CI)	8.1% (4.4–11.6)	26.0% (22.1 to −29.7)
Difference between D and C (95% CI)	—	

ID/A, intellectual difficulty/autism.

### Comparison of groups

Those prescribed an antipsychotic were more likely to have an intellectual difficulty/autism, be boys, have epilepsy, ADHD, and more likely to be in a special needs school (Table 1). Those with intellectual difficulty/autism were less likely to have a diagnosis of a psychotic disorder but more likely to have an aggression code and were prescribed antipsychotics at a younger age compared with those prescribed an antipsychotic without an intellectual difficulty/autism. Among those with intellectual difficulty/autism, the children with aggression codes and especially those with autism (75% of those on antipsychotics with an intellectual difficulty had a diagnosis of autism) were more likely to be prescribed an antipsychotic. Children with Tourette's (with or without an intellectual difficulty) were more likely to be prescribed an antipsychotic. However, among those with intellectual difficulties, children with Down's syndrome were less likely to be prescribed an antipsychotic. Those prescribed an antipsychotic and who had intellectual difficulty were less likely to be from a deprived area compared with those prescribed an antipsychotic without an intellectual difficulty. However, when all children with intellectual difficulty were considered, there was no significant difference in deprivation score among those prescribed an antipsychotic or those not prescribed antipsychotics.

### Odds of epilepsy, diabetes, asthma, or injury

#### Epilepsy

The main predictors of an epilepsy event in the GP/hospital data were intellectual difficulty/autism and being prescribed antipsychotics. Taking only those who were prescribed antipsychotics and comparing the years when on an antipsychotic with the years not on antipsychotic give a 1.4 higher rate of epilepsy (when on drug) controlling for gender, intellectual difficulties/autism, age, and aggression codes.

#### Diabetes

The odds of a child going to the GP or hospital with a diabetes event was 2.4 times higher if they were on an antipsychotic, this is controlling for intellectual difficulty/autism, gender, age, and aggression codes. For those children prescribed an antipsychotic, the odds of diabetes increased in the years they were on the antipsychotic and as they got older, but there was no difference of having an intellectual difficulty/autism or by gender.

#### Injury

The odds of injury increased when taking an antipsychotic drug. Taking only those on an antipsychotic showed lower injury rates among those with intellectual difficulty/autism compared with those without intellectual disabilities/autism (Table 2). Injury rates were higher in boys (compared to girls) and increased with age and the presence of aggression codes.

**Table 2.** Logistic Regression Comparing Odds of Event in the Year on an Antipsychotic Drug Compared with Years Not on Drug, Clustered by Individual

	*All children*		*Antipsychotic prescribed children*	
	*Using antipsychotic*	*Not using antipsychotic*	*Using antipsychotic*	*Not using antipsychotic*
(a) Epilepsy, odds of epilepsy event in years taking drug compared with years not taking drugs				
Number of events/number of visits	790/7473 (10.6%)	57,733/14,665,089 (0.37%)	790/7473 (10.6%)	293/4299 (6.8%)
	Odds ratio of epilepsy event in that year in those taking antipsychotic	95% CI	Odds ratio of epilepsy event in that year in those taking antipsychotic	95% CI
Accounting for clustering by individual	29.9	26.3–34.1^*^	1.6	1.2–2.0^*^
Adjusted for:				
Previous antipsychotic prescription (previous years)	5.6	4.8–6.6^*^	1.4	1.12–1.83^*^
Intellectual difficulty	19.2	18.0–20.2^*^	4.7	3.6–6.1^*^
Gender (0 = F, 1 = M)	0.88	0.85–0.92^*^	0.6	0.43 to 0.8^*^
Age	1.05	1.05–1.06^*^	1.00	0.96–1.04
Aggression	3.12	2.3–4.1^*^	1.68	1.0–2.0^*^

(b) Diabetes, odds of diabetes event in year taking drug compared with year not taking drugs				
Number of events/number of visits	81/7473 (1.1%)	34,338/14,665,089 (0.23%)	81/7473 (1.1%)	14/4299 (0.3%)
	Odds ratio of diabetes event in that year in those taking antipsychotic	95% CI	Odds ratio of diabetes event in that year in those taking antipsychotic	95% CI
Accounting for clustering by individual	4.8	4.6–5.0	3.25	1.5–7.6
Adjusted for:				
Previous antipsychotic prescription	2.4	1.67–3.5^*^	3.0	1.32–6.8^*^
Intellectual difficulty	2.0	1.7–2.3^*^	1.63	0.84–3.17
Gender (0 = F, 1 = M)	0.89	0.84–0.94^*^	0.72	0.37–1.37
Age	1.0	1.09–1.1^*^	1.26	1.12–1.42^*^
Aggression	1.87	1.15–3.05^*^	—	—

(c) Injury, odds of injury event in year taking drug compared with year not taking drugs				
Number of events/number of visits	1623/7473 (21.7%)	992,978/14,665,089 (6.8%)	1623/7473 (21.7%)	650/4299 (15.1%)
	Odds ratio of injury event in that year in those taking antipsychotic	95% CI	Odds ratio of injury event in that year in those taking antipsychotic	95% CI
Accounting for clustering by individual	3.8	3.6–4.1	1.6	1.4–1.7
Adjusted for:				
Previous antipsychotic prescription	3.04	2.8–3.2^*^	1.53	1.36–1.72^*^
Intellectual difficulty	1.12	1.09–1.15^*^	0.56	0.48–0.65^*^
Gender (0 = F, 1 = M)	1.27	1.26–1.28^*^	0.8	0.7–0.9^*^
Age	1.023	1.02–1.02^*^	1.07	1.05–1.09^*^
Aggression	3.6	3.3–4.0^*^	1.76	1.24–2.5^*^

(d) Respiratory infection, odds of respiratory infection event in year taking drug compared with year not taking drugs				
Number of events/number of visits	2579/7473 (34.5%)	3,424,618/14,665,089 (23.4%)	2579/7473 (34.5%)	1604/4299 (37.3%)
	Odds ratio of respiratory event in that year in those taking antipsychotic	95% CI	Odds ratio of respiratory event in that year in those taking antipsychotic	95% CI
Accounting for clustering by individual	1.72	1.6–1.8	0.88	0.7–1.01
Adjusted for:				
Previous antipsychotic prescription	2.06	1.9–2.23^*^	0.90	0.73–1.03
Intellectual difficulty	2.6	2.5–2.6^*^	1.53	1.28–1.83^*^
Gender (0 = F, 1 = M)	0.88	0.87–0.88^*^	0.63	0.54–0.75^*^
Age	0.92	0.92–0.92^*^	0.9	0.88–0.92^*^
Aggression	1.77	1.62–1.92^*^	1.0	0.71–1.44

(e) Depression, odds of depression event in year taking drug compared with year not taking drugs				
Number of events/number of visits	750/7473 (10%)	28,749/14,665,089 (0.2%)	750/7473 (10%)	119/4299 (2.8%)
	Odds ratio of depression event in that year in those taking antipsychotic	95% CI	Odds ratio of depression event in that year in those taking antipsychotic	95% CI
Accounting for clustering by individual	56.8	52.1–61.9	3.9	3.2–4.8
Adjusted for:				
Previous antipsychotic prescription	21.18	19.1–23.3^*^	3.7	2.98–4.5^*^
Intellectual difficulty	0.96	0.84–1.11^*^	0.21	0.14–0.28^*^
Gender (0 = F, 1 = M)	0.32	0.31–0.33^*^	0.40	0.34–0.47^*^
Age	1.56	1.54–01.56^*^	1.35	1.33–1.4^*^
Aggression	11.7	8.88–15.38^*^	2.36	1.46–3.83^*^

^*^ Significant.

#### Respiratory infection

The odds of respiratory infection increased with antipsychotic prescription compared with those not on an antipsychotic. However, respiratory infection was not found to be higher in the years on the antipsychotic compared with years not on the drug for those treated with this medication.

#### Depression

The odds of depression were higher in those on antipsychotics and higher in the years on an antipsychotic compared with the years not on an antipsychotic. Depression increases with age and is higher in those with aggression codes but lower in boys and those with intellectual difficulty/autism.

#### Prior event rate ratio

Children on an antipsychotic were more likely to attend their GP for epilepsy and/or diabetes events compared with those not using an antipsychotic and compared to the period before the antipsychotic prescription (Table 3). However, they were less likely to have an inpatient stay in hospital for epilepsy or diabetes after their antipsychotic compared with before their antipsychotic. The rate of respiratory conditions was higher for those using an antipsychotic compared to controls in the after period compared with the before drug period. This was found for both those with intellectual difficulty/autism and those without. Those with intellectual difficulty/autism were more likely to go to their GP and to hospital with injury and more likely to go to hospital with depression while using an antipsychotic (compared with the period before their antipsychotic), but this was not seen for the general population of children having an antipsychotic. In fact, in the general population, the use of antipsychotics was associated with lower levels of depression-related attendance at the GP and at hospital.

**Table 3.** Prior Event Rate Ratio for a Range of Outcomes Before and After Antipsychotic Compared with Nonantipsychotic Control

*Time to first epilepsy event over 3 years of follow-up (GP)*				
*Total population*		*Antipsychotic (*n* = 2419)*	*Nonantipsychotic (*n* = 1,323,759)*	*PERR: postdrug/prior drug ratio (95% CI)*
Prior drug	Number of events	167/6884	5633/3,963,937	
	Rate	2.42 per 100	0.14 per 100	1.27 (1.24–1.29)^*^
Postdrug	Number of events	224/6156.4	5763/3,436,106	
	Rate	3.63 per 100	0.16 per 100	

*Intellectual difficulty/autism*		*(*n* = 647)*	*(*n* = 26,065)*	
Prior drug	Number of events	104/1665	1260/75,480	
	Rate	6.25 per 100	1.67 per 100	1.06 (1.059–1.11)^*^
Postdrug	Number of events	112/1587	1077/60,732	
	Rate	7.06 per 100	1.77 per 100	

*Time to epilepsy event over 3 years of follow-up (HOSPITAL)*				
*Total population*		*Antipsychotic (*n* = 2419)*	*Nonantipsychotic (*n* = 1,326,178)*	*PERR: postdrug/prior drug ratio (95% CI)*
Prior drug	Number of events	86/7115.6	2300/1,323,756	
	Rate	1.2 per 100	0.058 per 100	0.78 (0.74–0.80)^*^
Postdrug	Number of events	64/6260.1	2155/3438951.5	
	Rate	1.023 per 100	0.062 per 100	

*Intellectual difficulty/autism*		*(*n* = 647)*	*(*n* = 26,100)*	
Prior drug	Number of events	62/1833	796/76,884	
	Rate	3.38 per 100	1.0 per 100	0.77 (0.73–0.81)^*^
Postdrug	Number of events	42/1602	630/60,722	
	Rate	2.62 per 100	1.037 per 100	

*Diabetes events over 3 years of follow-up (GP)*				
*Total population*		*Antipsychotic (*n* = 2419)*	*Nonantipsychotic (*n* = 132,745)*	*PERR: postdrug/prior drug ratio (95% CI)*
Prior drug	Number of events	11/7238.7	1982/3971067.6	
	Rate	0.15 per 100	0.05 per 100	1.51 (1.5–1.8)^*^
Postdrug	Number of events	20/6310.6	23713439638.5	
	Rate	0.31 per 100	0.07 per 100	

*Diabetes events over 3 years of follow-up (HOSPITAL)*				
*Total population*		*Antipsychotic (*n* = 2419)*	*Nonantipsychotic (*n* = 1,323,755)*	*PERR: postdrug/prior drug ratio (95% CI)*
Prior drug	Number of events	26/7229	2797/3,970,410	
	Rate	0.35 per 100	0.07 per 100	0.65 (0.67–0.79)^*^
Postdrug	Number of events	18/6305.6	2949/3437858.4	
	Rate	0.29 per 100	0.086 per 100	

*Time to first injury event over 3 years of follow-up (GP)*				
*Total population*		*Antipsychotic (*n* = 2419)*	*Nonantipsychotic (*n* = 1,323,554)*	*PERR: postdrug/prior drug ratio (95% CI)*
Prior drug	Number of events	197/6999.7	50,440/1,323,684	
	Rate	2.8 per 100	1.2 per 100	0.90 (0.89–0.92)^*^
Postdrug	Number of events	149/6141.5	24,561/3409171.4	
	Rate	2.4 per 100	0.72 per 100	

*Intellectual difficulty/autism*		*(*n* = 647)*	*(*n* = 26,094)*	
Prior drug	Number of events	167/1679.4	4917/70,488	
	Rate	9.94 per 100	6.9 per 100	1.154 (1.1–1.17)^*^
Postdrug	Number of events	146/1483.3	3407/56,959	
	Rate	9.84 per 100	5.98 per 100	

*Time to first injury event over 3 years of follow-up (HOSPITAL)*				
*Total population*		*Antipsychotic (*n* = 2419)*	*Nonantipsychotic (*n* = 1,323,726)*	*PERR: Postdrug/prior drug ratio (95% CI)*
Before drug	Number of events	397/6754	36,026/3,920,356	
	Rate	5.88 per 100	0.92 per 100	1.019 (0.99–1.03)
Postdrug	Number of events	353/5939	30,975/3,397,039	
	Rate	5.94 per 100	0.91 per 100	

*Intellectual difficulty/autism*		*(*n* = 647)*	*(*n* = 26,097)*	
Prior drug	Number of events	64/1851	1486/76,063	
	Rate	3.46 per 100	1.95 per 100	1.038 (1.011–1.084)^*^
Postdrug	Number of events	49/1583	1011/60,050	
	Rate	3.09 per 100	1.68 per 100	

*Time to first respiratory event over 3 years of follow-up (GP)*				
*Total population*		*Antipsychotic (*n* = 2419)*	*Nonantipsychotic (*n* = 1,322,985)*	*PERR: postdrug/prior drug ratio (95% CI)*
Prior drug	Number of events	808/5809.5	355,371/3295466.5	
	Rate	13.9 per 100	10.78 per 100	1.27 (0.25–1.30)^*^
Postdrug	Number of events	665/5531.9	232,568/3170022.7	
	Rate	12.0 per 100	7.33 per 100	

*Intellectual difficulty/autism*		*(*n* = 646)*	*(*n* = 26,070)*	
Prior drug	Number of events	246/1482.4	12,008/54426.8	
	Rate	16.59 per 100	22.1 per 100	1.20 (1.15–1.22)^*^
Postdrug	Number of events	163/1448.8	6749/54153.3	
	Rate	11.25 per 100	12.46 per 100	

*Time to first respiratory event over 3 years of follow-up (HOSPITAL)*				
*Total population*		*Antipsychotic (*n* = 2419)*	*Nonantipsychotic (*n* = 1,323,684)*	*PERR: postdrug/prior drug ratio (95% CI)*
Prior drug	Number of events	197/6999.7	50,440/3884917.5	
	Rate	2.8 per 100	1.3 per 100	1.55 (1.51–1.598)^*^
Postdrug	Number of events	149/6141.5	24,561/3409171.4	
	Rate	5.6 per 100	0.72 per 100	

*Intellectual difficulty/autism*		*(*n* = 647)*	*(*n* = 26,097)*	
Prior drug	Number of events	68/1847.5	3194/72,384	
	Rate	3.4 per 100	4.4 per 100	1.475 (1.37–1.53)^*^
Postdrug	Number of events	44/1586.7	1348/59,812	
	Rate	2.8 per 100	2.25 per 100	

*Time to first depression event over 3 years of follow-up (GP)*				
*Total population*		*Antipsychotic (*n* = 2419)*	*Nonantipsychotic (*n* = 1,322,985)*	*PERR: postdrug/prior drug ratio (95% CI)*
Prior drug	Number of events	274/7034	1707/3,973,031	
	Rate	3.9 per 100	0.04 per 100	0.23 (0.21–0.23)^*^
Postdrug	Number of events	252/6959	7036/3,429,799	
	Rate	4.16 per 100	0.25 per 100	

*Intellectual difficulty/autism*		*(*n* = 646)*	*(*n* = 26,070)*	
Prior drug	Number of events	23/1924	21/78,344	
	Rate	1.195 per 100	0.026 per 100	0.278 (0.25–0.31)^*^
Postdrug	Number of events	20/1619	61/61299.3	
	Rate	1.23 per 100	0.1 per 100	

*Time to first depression event over 3 years of follow-up (HOSPITAL)*				
*Total population*		*Antipsychotic (*n* = 2419)*	*Nonantipsychotic (*n* = 1,323,684)*	*PERR: postdrug/prior drug ratio (95% CI)*
Prior drug	Number of events	116/7187	582/3,974,114	
	Rate	1.6 per 100	0.015 per 100	0.55 (0.51–0.59)^*^
Postdrug	Number of events	139/6183	1274/3,439,784	
	Rate	2.25 per 100	0.037 per 100	

*Intellectual difficulty/autism*		*(*n* = 647)*	*(*n* = 26,069)*	
Prior drug	Number of events	9/1935	27/78,339	
	Rate	0.46 per 100	0.034 per 100	1.59 (1.37–1.96)^*^
Postdrug	Number of events	17/1623	30/61,368	
	Rate	1.04 per 100	0.048 per 100	

GP, general practice; PERR, prior event rate ratio.

## Discussion

This study shows the rates of adverse events in 3028 children prescribed antipsychotics and can be used to inform the debate regarding the use of these medications and the implications for the patient and for services in the NHS. The study found that children with intellectual difficulty/autism are more likely to be prescribed an antipsychotic compared with children without intellectual difficulties, and those with autism are the most likely to have antipsychotics. After the prescription of antipsychotics, the number of contacts at hospital and in the GP for respiratory disease increased compared with expected numbers, as respiratory disease decreases with age, so declines in those not prescribed an antipsychotic but does not decline in those on an antipsychotic. Thus, looking at the odds ratio of respiratory disease in those taking antipsychotics (Table 2) does not show higher odds of respiratory infection after antipsychotic compared with before, but looking at rates compared to nonantipsychotic patients (Tables 2 and 3) does show relatively higher rates of respiratory disease. The number of contacts for epilepsy and diabetes increases for the GP, but the number of inpatient hospital visits for epilepsy or diabetes decreases. Children with intellectual difficulty/autism are more likely to have injuries when prescribed an antipsychotic and more likely to go into hospital with depression compared with children without intellectual difficulty/autism. However, those without an intellectual difficulty/autism had lower rates of depression after the antipsychotic prescription.

This study does find evidence that supports evidence from trials that antipsychotics are associated with high rates of respiratory events (Pandina et al. 2007) and provides an assessment of the rate of infection in this group compared with a control. The study gives an estimated additional rate of epilepsy events and diabetes events to inform healthcare planning and decision-making. The findings suggest that children with intellectual difficulty/autism are prescribed antipsychotics predominately for behavioral control/aggression rather than psychotic disorder and predominately in those with autism. Arguably, this is in an environment where some studies show no significant benefit for managing behavioral problems (Ahmed et al. 2000; Tyrer et al. 2008; de Kuijper et al. 2014), and evidence supporting behavioral control is limited but there are significant positive effects on weight and bone density on discontinuation (de Kuijper et al. 2013) of the drug. It could be argued that there is limited evidence that antipsychotics can help manage this behavior (Brylewski and Duggan 2004) and arguably that effects on behavior may be due to the adverse event of somnolence as it is unclear whether risperidone improves the core social and communication impairment of autism (Posey et al. 2008). For example, the increase in injury rates among those with intellectual difficulty/autism may be due to somnolence and this hypothesis may be supported by the finding of higher depression in this population after antipsychotics. The reduction in injury in those without intellectual difficulty/autism may be due to lowering self-harm behavior in this population and this may be supported by the lower rate of depression in this population after antipsychotic use.

The findings of this study showed that 2.8% of children with intellectual difficulty and/or autism were prescribed an antipsychotic; these findings are similar to that seen in England (Glover et al. 2015) where 2.4% of children and young people with an intellectual difficulty/autism were given antipsychotic drugs. In this report from England, the prescriptions rate then rose through adult life. This study finds that seizures as measured in the GP data may increase when using an antipsychotic and this finding could have been predicted as antipsychotic lower seizure threshold (Barry and Huynh 2001; Kavanagh et al. 2015). The finding that diabetes events (in GP data) increase with antipsychotic use is also predictable due to known metabolic adverse effects of antipsychotics, which have been shown to affect children more than adults (Correll and Carlson 2006; Vitiello 2009). However, when using an antipsychotic, the number of hospital admissions for epilepsy or diabetes decreases. It is possible that the lowering of admissions may be due to those on antipsychotics having more outpatient contacts rather than emergency admissions. The outpatient records are not routinely available by specialty and so were not examined in this study. Therefore, we are not able to evaluate if total hospital contacts change but could only say that hospital admissions decrease after antipsychotic prescription and with general practitioners taking on more diabetes care, especially that related to type 2 insulin resistance; this may be a predictable finding. However, one limitation of this study is we could only examine GP and hospital admissions but could not capture other types of contact with the health system such as outpatients, emergency department, or others such as child and adolescent mental health services contacts. Antipsychotics can be prescribed in these other healthcare contacts and in hospital admissions, but these prescriptions are missing from this analysis (unless they are then taken over by the GP). Therefore, the findings in this study will be an underestimate of the proportion of children prescribed an antipsychotic and their healthcare contacts. Certain aspects of this study were underpowered, for example, we could not examine diabetes events in those with intellectual difficulty/autism due to small numbers of events (e.g., less than 10 in the prior events of those using antipsychotics). Therefore, future work could extend this analysis to a larger population such as that in England and Scotland to confirm and validate findings as an increase in diabetes not only leads to a long-term chronic condition in those affected but also has implications for the NHS. In addition, this study examined 3 years of follow-up and this is likely to be too short to really pick up a long-term diabetes risk. It did not examine weight gain, insulin resistance, or metabolic syndrome but only picked up end-stage diabetes and it is likely that true diabetes risk may not be seen until adulthood; a 3-year period is too short. The PERR does assume that the prior event does not distort prescribing behavior and, in theory, a child with prior diabetes or epilepsy would be less likely to be prescribed an antipsychotic. However, looking at the prior rate in the PERR, there is no evidence that prescriptions are less in those with existing comorbidities. In addition, this analysis did not take into account dose or break down the antipsychotics by type and it is likely that the rate of adverse events will be influenced by these factors. Finally, the definition of intellectual difficulty is not straightforward using routine data and some of the diagnostic codes used may be broad and there is potential for confounding in that some individuals without intellectual difficulty but with autism or with borderline problems are included.

## Conclusions

Antipsychotics are prescribed predominantly to those with intellectual difficulty/autism and there is evidence that they can increase rates of respiratory disease, epilepsy, diabetes for all, and of hospital-admitted depression and injury in the intellectual difficulty/autism population. Children in special schools, those with autism and those with aggression, are especially likely to be prescribed an antipsychotic and this may be a marker that they have more severe behavioral problems. Children with intellectual difficulties are likely to be prescribed antipsychotic at a younger age and for a longer period compared with those without intellectual difficulties. There is evidence of higher rates of long-term adverse events and this evidence can be used to inform healthcare provision and costs associated with the use of antipsychotics.

## Clinical Significance

This research finds antipsychotics are prescribed mainly for children with learning difficulty and in this population there are higher rates of adverse events, they are used at a younger age, and for a longer time period. This work adds to the evidence base of the use of antipsychotics in children.

## Supplementary Material

Supplemental data

## Data Sharing

All data are available through the SAIL gateway www.saildatabank.com
