# Supplementary material for: Characteristics of Children Prescribed Antipsychotics: Analysis of Routinely Collected Data
Source: J Child Adolesc Psychopharmacol. 2018 Apr 1;28(3):180–91. doi: 10.1089/cap.2017.0003 (PMC5905863; doi:10.1089/cap.2017.0003)
Supplement: Supplemental data [file Supp_Data.zip › Supp_file 1.docx]

Supplementary file 1: Read codes and ICD-10 codes use to define conditions and medications.

Codes for intellectual disability:

| **DATABASE** | **CODES** | **SUB_GROUP** |
| --- | --- | --- |
| Congenital abnormality | ANOMALY_CD = 'F79X' | MENTAL_RETARDATION |
| Congenital abnormality | ANOMALY_CD = 'F82X' | MOTOR_DELAY |
| Congenital abnormality | ANOMALY_CD = 'F83.X' | LD |
| Congenital abnormality | SYNDROME_ICD = 'F79X' | MENTAL_RETARDATION |
| Congenital abnormality | SYNDROME_ICD = 'F80.2' | DEVELOPMENTAL DELAY |
| Congenital abnormality | SYNDROME_ICD = 'F80.3' | DEVELOPMENTAL DELAY |
| Congenital abnormality | SYNDROME_ICD = 'F80.9' | DEVELOPMENTAL DELAY |
| Congenital abnormality | SYNDROME_ICD = 'F81.9' | DEVELOPMENTAL DELAY |
| Congenital abnormality | SYNDROME_ICD = 'F82X' | MOTOR_DELAY |
| Congenital abnormality | SYNDROME_ICD = 'F83.X' | LD |
| Congenital abnormality | SYNDROME_ICD = 'F89.X' | DEVELOPMENTAL DELAY |
| Congenital abnormality | SYNDROME_ICD_2 = 'F79X' | MENTAL_RETARDATION |
| Congenital abnormality | SYNDROME_ICD_2 = 'F80.2' | DEVELOPMENTAL DELAY |
| Congenital abnormality | SYNDROME_ICD_2 = 'F80.3' | DEVELOPMENTAL DELAY |
| Congenital abnormality | SYNDROME_ICD_2 = 'F80.9' | DEVELOPMENTAL DELAY |
| Congenital abnormality | SYNDROME_ICD_2 = 'F81.9' | DEVELOPMENTAL DELAY |
| Congenital abnormality | SYNDROME_ICD_2 = 'F82X' | MOTOR_DELAY |
| Congenital abnormality | SYNDROME_ICD_2 = 'F83.X' | LD |
| Congenital abnormality | SYNDROME_ICD_2 = 'F89.X' | DEVELOPMENTAL DELAY |
| HOSPITAL ADMISSION | DIAG_CD_1234 = 'F701' -- Mld mental retard sig impairment behav req attent /treat | MENTAL_RETARDATION |
| HOSPITAL ADMISSION | DIAG_CD_1234 = 'F700' -- Mld mental retard with statement no min impairm behav | MENTAL_RETARDATION |
| HOSPITAL ADMISSION | DIAG_CD_1234 = 'F708' -- Mild mental retardation, other impairments of behaviour | MENTAL_RETARDATION |
| HOSPITAL ADMISSION | DIAG_CD_1234 = 'F709' -- Mild mental retardation without mention of impairment behav | MENTAL_RETARDATION |
| HOSPITAL ADMISSION | DIAG_CD_1234 = 'F710' -- Mod mental retard with statement no min impairm behav | MENTAL_RETARDATION |
| HOSPITAL ADMISSION | DIAG_CD_1234 = 'F711' -- Mod mental retard sig impairm of behav req attent /treat | MENTAL_RETARDATION |
| HOSPITAL ADMISSION | DIAG_CD_1234 = 'F718' -- Moderate mental retardation, other impairments of behaviour | MENTAL_RETARDATION |
| HOSPITAL ADMISSION | DIAG_CD_1234 = 'F719' -- Mod mental retard without mention of impairment of behav | MENTAL_RETARDATION |
| HOSPITAL ADMISSION | DIAG_CD_1234 = 'F720' -- Sev mental retard with statement no min impairm behav | MENTAL_RETARDATION |
| HOSPITAL ADMISSION | DIAG_CD_1234 = 'F721' -- Sev mental retard sign impairm behav req attent /treatment | MENTAL_RETARDATION |
| HOSPITAL ADMISSION | DIAG_CD_1234 = 'F728' -- Severe mental retardation, other impairments of behaviour | MENTAL_RETARDATION |
| HOSPITAL ADMISSION | DIAG_CD_1234 = 'F729' -- Severe mental retard without mention of impairment of behav | MENTAL_RETARDATION |
| HOSPITAL ADMISSION | DIAG_CD_1234 = 'F730' -- Prof mental retard with statement no min impairm behav | MENTAL_RETARDATION |
| HOSPITAL ADMISSION | DIAG_CD_1234 = 'F731' -- Prof mental retard sig impairm behav req attent/treatment | MENTAL_RETARDATION |
| HOSPITAL ADMISSION | DIAG_CD_1234 = 'F738' -- Profound mental retardation, other impairments of behaviour | MENTAL_RETARDATION |
| HOSPITAL ADMISSION | DIAG_CD_1234 = 'F739' -- Profound mental retard without mention of impairm behav | MENTAL_RETARDATION |
| HOSPITAL ADMISSION | DIAG_CD_1234 = 'F780' -- Oth mental retard with statment of no min impairm behav | MENTAL_RETARDATION |
| HOSPITAL ADMISSION | DIAG_CD_1234 = 'F781' -- Oth mental retard sig impairm of behav req attent / treat | MENTAL_RETARDATION |
| HOSPITAL ADMISSION | DIAG_CD_1234 = 'F788' -- Other mental retardation, other impairments of behaviour | MENTAL_RETARDATION |
| HOSPITAL ADMISSION | DIAG_CD_1234 = 'F789' -- Other mental retardation with other impairments of behaviour | MENTAL_RETARDATION |
| HOSPITAL ADMISSION | DIAG_CD_1234 = 'F790' -- Unspec mental retard with statement no min impair behav | MENTAL_RETARDATION |
| HOSPITAL ADMISSION | DIAG_CD_1234 = 'F791' -- Unspec mental retard sign impairm behav requir attent/treat | MENTAL_RETARDATION |
| HOSPITAL ADMISSION | DIAG_CD_1234 = 'F798' -- Unspec mental retardation | MENTAL_RETARDATION |
| HOSPITAL ADMISSION | DIAG_CD_1234 = 'F799' -- Unspec mental retard without mention of impairment of behav | MENTAL_RETARDATION |
| HOSPITAL ADMISSION | DIAG_CD_1234 = 'F800' -- Specific speech articulation disorder | DEVELOPMENTAL_DISORDER |
| HOSPITAL ADMISSION | DIAG_CD_1234 = 'F801' -- Expressive language disorder | DEVELOPMENTAL_DISORDER |
| HOSPITAL ADMISSION | DIAG_CD_1234 = 'F802' -- Receptive language disorder | DEVELOPMENTAL_DISORDER |
| HOSPITAL ADMISSION | DIAG_CD_1234 = 'F803' -- Acquired aphasia with epilepsy [Landau-Kleffner] | DEVELOPMENTAL_DISORDER |
| HOSPITAL ADMISSION | DIAG_CD_1234 = 'F808' -- Other developmental disorders of speech and language | DEVELOPMENTAL_DISORDER |
| HOSPITAL ADMISSION | DIAG_CD_1234 = 'F809' -- Developmental disorder of speech and language, unspecified | DEVELOPMENTAL_DISORDER |
| HOSPITAL ADMISSION | DIAG_CD_1234 = 'F810' -- Specific reading disorder | DEVELOPMENTAL_DISORDER |
| HOSPITAL ADMISSION | DIAG_CD_1234 = 'F811' -- Specific spelling disorder | DEVELOPMENTAL_DISORDER |
| HOSPITAL ADMISSION | DIAG_CD_1234 = 'F812' -- Specific disorder of arithmetical skills | DEVELOPMENTAL_DISORDER |
| HOSPITAL ADMISSION | DIAG_CD_1234 = 'F813' -- Mixed disorder of scholastic skills | DEVELOPMENTAL_DISORDER |
| HOSPITAL ADMISSION | DIAG_CD_1234 = 'F818' -- Other developmental disorders of scholastic skills | DEVELOPMENTAL_DISORDER |
| HOSPITAL ADMISSION | DIAG_CD_1234 = 'F819' -- Developmental disorder of scholastic skills, unspecified | DEVELOPMENTAL_DISORDER |
| HOSPITAL ADMISSION | DIAG_CD_1234 = 'F82X' -- Specific developmental disorder of motfunction | DEVELOPMENTAL_DISORDER |
| HOSPITAL ADMISSION | DIAG_CD_1234 = 'F83X' -- Mixed specific developmental disorders | DEVELOPMENTAL_DISORDER |
|  |  |  |
|  |  |  |
| HOSPITAL ADMISSION | DIAG_CD_1234 = 'F842' -- Rett's syndrome | DEVELOPMENTAL_DISORDER |
| HOSPITAL ADMISSION | DIAG_CD_1234 = 'F843' -- Other childhood disintegrative disorder | DEVELOPMENTAL_DISORDER |
| HOSPITAL ADMISSION | DIAG_CD_1234 = 'F844' -- Overactive disord assoc mental retard and stereotype movts | DEVELOPMENTAL_DISORDER |
|  |  |  |
| HOSPITAL ADMISSION | DIAG_CD_1234 = 'F848' -- Other pervasive developmental disorders | DEVELOPMENTAL_DISORDER |
| HOSPITAL ADMISSION | DIAG_CD_1234 = 'F849' -- Pervasive developmental disorder, unspecified | DEVELOPMENTAL_DISORDER |
| HOSPITAL ADMISSION | DIAG_CD_1234 = 'Q910' -- Trisomy 18, meiotic nondisjunction | Q91_6 |
| HOSPITAL ADMISSION | DIAG_CD_1234 = 'Q911' -- Trisomy 18, mosaicism (mitotic nondisjunction) | Q91_6 |
| HOSPITAL ADMISSION | DIAG_CD_1234 = 'Q912' -- Trisomy 18, translocation | Q91_6 |
| HOSPITAL ADMISSION | DIAG_CD_1234 = 'Q913' -- Edwards' syndrome, unspecified | Q91_6 |
| HOSPITAL ADMISSION | DIAG_CD_1234 = 'Q914' -- Trisomy 13, meiotic nondisjunction | Q91_6 |
| HOSPITAL ADMISSION | DIAG_CD_1234 = 'Q915' -- Trisomy 13, mosaicism (mitotic nondisjunction) | Q91_6 |
| HOSPITAL ADMISSION | DIAG_CD_1234 = 'Q916' -- Trisomy 13, translocation | Q91_6 |
| HOSPITAL ADMISSION | DIAG_CD_1234 = 'Q917' -- Patau's syndrome, unspecified | Q91_6 |
| HOSPITAL ADMISSION | DIAG_CD_1234 = 'Q920' -- Whole chromosome trisomy, meiotic nondisjunction | Q91_6 |
| HOSPITAL ADMISSION | DIAG_CD_1234 = 'Q921' -- Whole chromosome trisomy, mosaicism (mitotic nondisjunction) | Q91_6 |
| HOSPITAL ADMISSION | DIAG_CD_1234 = 'Q922' -- Majpartial trisomy | Q91_6 |
| HOSPITAL ADMISSION | DIAG_CD_1234 = 'Q923' -- Minpartial trisomy | Q91_6 |
| HOSPITAL ADMISSION | DIAG_CD_1234 = 'Q924' -- Duplications seen only at prometaphase | Q91_6 |
| HOSPITAL ADMISSION | DIAG_CD_1234 = 'Q925' -- Duplications with other complex rearrangements | Q91_6 |
| HOSPITAL ADMISSION | DIAG_CD_1234 = 'Q926' -- Extra marker chromosomes | Q91_6 |
| HOSPITAL ADMISSION | DIAG_CD_1234 = 'Q927' -- Triploidy and polyploidy | Q91_6 |
| HOSPITAL ADMISSION | DIAG_CD_1234 = 'Q928' -- Other specified trisomies and partial trisomies of autosomes | Q91_6 |
| HOSPITAL ADMISSION | DIAG_CD_1234 = 'Q929' -- Trisomy and partial trisomy of autosomes, unspecified | Q91_6 |
| HOSPITAL ADMISSION | DIAG_CD_1234 = 'Q930' -- Whole chromosome monosomy, meiotic nondisjunction | Q91_6 |
| HOSPITAL ADMISSION | DIAG_CD_1234 = 'Q931' -- Whole chrom monosomy mosaicism (mitotic nondisjunction) | Q91_6 |
| HOSPITAL ADMISSION | DIAG_CD_1234 = 'Q932' -- Chromosome replaced with ring dicentric | Q91_6 |
| HOSPITAL ADMISSION | DIAG_CD_1234 = 'Q933' -- Deletion of short arm of chromosome 4 | Q91_6 |
| HOSPITAL ADMISSION | DIAG_CD_1234 = 'Q934' -- Deletion of short arm of chromosome 5 | Q91_6 |
| HOSPITAL ADMISSION | DIAG_CD_1234 = 'Q935' -- Other deletions of part of a chromosome | Q91_6 |
| HOSPITAL ADMISSION | DIAG_CD_1234 = 'Q936' -- Deletions seen only at prometaphase | Q91_6 |
| HOSPITAL ADMISSION | DIAG_CD_1234 = 'Q937' -- Deletions with other complex rearrangements | Q91_6 |
| HOSPITAL ADMISSION | DIAG_CD_1234 = 'Q938' -- Other deletions from the autosomes | Q91_6 |
| HOSPITAL ADMISSION | DIAG_CD_1234 = 'Q939' -- Deletion from autosomes, unspecified | Q91_6 |
| HOSPITAL ADMISSION | DIAG_CD_1234 = 'Q950' -- Balanced translocation and insertion in normal individual | Q91_6 |
| HOSPITAL ADMISSION | DIAG_CD_1234 = 'Q951' -- Chromosome inversion in normal individual | Q91_6 |
| HOSPITAL ADMISSION | DIAG_CD_1234 = 'Q952' -- Balanced autosomal rearrangement in abnormal individual | Q91_6 |
| HOSPITAL ADMISSION | DIAG_CD_1234 = 'Q953' -- Balanced sex/autosomal rearrangement in abnormal individual | Q91_6 |
| HOSPITAL ADMISSION | DIAG_CD_1234 = 'Q954' -- Individuals with marker heterochromatin | Q91_6 |
| HOSPITAL ADMISSION | DIAG_CD_1234 = 'Q955' -- Individuals with autosomal fragile site | Q91_6 |
| HOSPITAL ADMISSION | DIAG_CD_1234 = 'Q958' -- Other balanced rearrangements and structural markers | Q91_6 |
| HOSPITAL ADMISSION | DIAG_CD_1234 = 'Q959' -- Balanced rearrangement and structural marker, unspecified | Q91_6 |
| HOSPITAL ADMISSION | DIAG_CD_1234 = 'Q960' -- Karyotype 45,X | Q91_6 |
| HOSPITAL ADMISSION | DIAG_CD_1234 = 'Q961' -- Karyotype 46,X iso (Xq) | Q91_6 |
| HOSPITAL ADMISSION | DIAG_CD_1234 = 'Q962' -- Karyotype 46,X with abnormal sex chromosome, except iso (Xq) | Q91_6 |
| HOSPITAL ADMISSION | DIAG_CD_1234 = 'Q963' -- Mosaicism, 45,X/46,XX XY | Q91_6 |
| HOSPITAL ADMISSION | DIAG_CD_1234 = 'Q964' -- Mosaicism 45X/oth cell line(s) with abnorm sex chromosome | Q91_6 |
| HOSPITAL ADMISSION | DIAG_CD_1234 = 'Q968' -- Other variants of Turner's syndrome | Q91_6 |
| HOSPITAL ADMISSION | DIAG_CD_1234 = 'Q969' -- Turner's syndrome, unspecified | Q91_6 |
| HOSPITAL ADMISSION | DIAG_CD_1234 = 'Q970' -- Karyotype 47,XXX | Q9_Other_chromosome_abnormalities |
| HOSPITAL ADMISSION | DIAG_CD_1234 = 'Q971' -- Female with more than three X chromosomes | Q9_Other_chromosome_abnormalities |
| HOSPITAL ADMISSION | DIAG_CD_1234 = 'Q972' -- Mosaicism, lines with various numbers of X chromosomes | Q9_Other_chromosome_abnormalities |
| HOSPITAL ADMISSION | DIAG_CD_1234 = 'Q973' -- Female with 46,XY karyotype | Q9_Other_chromosome_abnormalities |
| HOSPITAL ADMISSION | DIAG_CD_1234 = 'Q978' -- Oth spec sex chromosome abnormalities female phrenotype | Q9_Other_chromosome_abnormalities |
| HOSPITAL ADMISSION | DIAG_CD_1234 = 'Q979' -- Sex chromosome abnormality, female phenotype, unspecified | Q9_Other_chromosome_abnormalities |
| HOSPITAL ADMISSION | DIAG_CD_1234 = 'Q980' -- Klinefelter's syndrome karyotype 47,XXY | Q9_Other_chromosome_abnormalities |
| HOSPITAL ADMISSION | DIAG_CD_1234 = 'Q981' -- Klinefelter's syn male with more than two X chromosomes | Q9_Other_chromosome_abnormalities |
| HOSPITAL ADMISSION | DIAG_CD_1234 = 'Q982' -- Klinefelter's syndrome, male with 46,XX karyotype | Q9_Other_chromosome_abnormalities |
| HOSPITAL ADMISSION | DIAG_CD_1234 = 'Q983' -- Other male with 46,XX karyotype | Q9_Other_chromosome_abnormalities |
| HOSPITAL ADMISSION | DIAG_CD_1234 = 'Q984' -- Klinefelter's syndrome, unspecified | Q9_Other_chromosome_abnormalities |
| HOSPITAL ADMISSION | DIAG_CD_1234 = 'Q985' -- Karyotype 47,XYY | Q9_Other_chromosome_abnormalities |
| HOSPITAL ADMISSION | DIAG_CD_1234 = 'Q986' -- Male with structurally abnormal sex chromosome | Q9_Other_chromosome_abnormalities |
| HOSPITAL ADMISSION | DIAG_CD_1234 = 'Q987' -- Male with sex chromosome mosaicism | Q9_Other_chromosome_abnormalities |
| HOSPITAL ADMISSION | DIAG_CD_1234 = 'Q988' -- Other specified sex chromosome abnormalities, male phenotype | Q9_Other_chromosome_abnormalities |
| HOSPITAL ADMISSION | DIAG_CD_1234 = 'Q989' -- Sex chromosome abnormality, male phenotype, unspecified | Q9_Other_chromosome_abnormalities |
| HOSPITAL ADMISSION | DIAG_CD_1234 = 'Q990' -- Chimera 46,XX/46,XY | Q9_Other_chromosome_abnormalities |
| HOSPITAL ADMISSION | DIAG_CD_1234 = 'Q991' -- 46,XX true hermaphrodite | Q9_Other_chromosome_abnormalities |
| HOSPITAL ADMISSION | DIAG_CD_1234 = 'Q992' -- Fragile X chromosome | Q9_Other_chromosome_abnormalities |
| HOSPITAL ADMISSION | DIAG_CD_1234 = 'Q998' -- Other specified chromosome abnormalities | Q9_Other_chromosome_abnormalities |
| HOSPITAL ADMISSION | DIAG_CD_1234 = 'Q999' -- Chromosomal abnormality, unspecified | Q9_Other_chromosome_abnormalities |
| GP | EVENT_CD = '69DB.' -- Learning disability health examination |  |
| GP | EVENT_CD = '8Ce6.' -- Preferred place of care - learning disability unit |  |
| GP | EVENT_CD = '8H4f.' -- Referral to learning disabilities psychiatrist |  |
| GP | EVENT_CD = '8Hg2.' -- Discharge from learning disability team |  |
| GP | EVENT_CD = '8HHP.' -- Referral to learning disability team |  |
| GP | EVENT_CD = '918e.' -- On learning disability register |  |
| GP | EVENT_CD = '94Z9.' -- Preferred place of death: learning disability unit |  |
| GP | EVENT_CD = '9HB..' -- Learning disabilities administration status |  |
| GP | EVENT_CD = '9HB0.' -- Learning disabilities health action plan declined |  |
| GP | EVENT_CD = '9HB1.' -- Learning disabilities health action plan offered |  |
| GP | EVENT_CD = '9HB2.' -- Learning disabilities health action plan reviewed |  |
| GP | EVENT_CD = '9HB3.' -- Learning disabilities health assessment |  |
| GP | EVENT_CD = '9HB4.' -- Learning disabilities health action plan completed |  |
| GP | EVENT_CD = '9HB5.' -- Learning disabilities annual health assessment |  |
| GP | EVENT_CD = '9HB6.' -- Learning disabilities annual health assessment declined |  |
| GP | EVENT_CD = '9HB7.' -- Did not attend learning disabilities annual health assessmnt |  |
| GP | EVENT_CD = '9hL..' -- Exception reporting: learning disability quality indicators |  |
| GP | EVENT_CD = '9mA..' -- Learning disability annual health check invitation |  |
| GP | EVENT_CD = '9mA0.' -- Learning disability annual health check verbal invitation |  |
| GP | EVENT_CD = '9mA1.' -- Learning disability annual health check telephone invitation |  |
| GP | EVENT_CD = '9mA2.' -- Learning disability annual health check letter invitation |  |
| GP | EVENT_CD = '9mA20' -- Learning disability annual health check invtation 1st letter |  |
| GP | EVENT_CD = '9mA21' -- Learning disability annual health check invtation 2nd letter |  |
| GP | EVENT_CD = '9mA22' -- Learning disability annual health check invtation 3rd letter |  |
| GP | EVENT_CD = '9N0y.' -- Seen in learning disabilities clinic |  |
| GP | EVENT_CD = 'E2F2.' -- Other specific learning difficulty |  |
| GP | EVENT_CD = 'E3...' -- Mental retardation |  |
| GP | EVENT_CD = 'E30..' -- Mild mental retardation, IQ in range 50-70 |  |
| GP | EVENT_CD = 'E31..' -- Other specified mental retardation |  |
| GP | EVENT_CD = 'E310.' -- Moderate mental retardation, IQ in range 35-49 |  |
| GP | EVENT_CD = 'E311.' -- Severe mental retardation, IQ in range 20-34 |  |
| GP | EVENT_CD = 'E312.' -- Profound mental retardation with IQ less than 20 |  |
| GP | EVENT_CD = 'E31z.' -- Other specified mental retardation NOS |  |
| GP | EVENT_CD = 'E3y..' -- Other specified mental retardation |  |
| GP | EVENT_CD = 'E3z..' -- Mental retardation NOS |  |
| GP | EVENT_CD = 'Eu7..' -- [X]Mental retardation |  |
| GP | EVENT_CD = 'Eu70.' -- [X]Mild mental retardation |  |
| GP | EVENT_CD = 'Eu700' -- [X]Mld mental retard with statement no min impairm behav |  |
| GP | EVENT_CD = 'Eu701' -- [X]Mld mental retard sig impairment behav req attent/treatmt |  |
| GP | EVENT_CD = 'Eu70y' -- [X]Mild mental retardation, other impairments of behaviour |  |
| GP | EVENT_CD = 'Eu70z' -- [X]Mild mental retardation without mention impairment behav |  |
| GP | EVENT_CD = 'Eu71.' -- [X]Moderate mental retardation |  |
| GP | EVENT_CD = 'Eu710' -- [X]Mod mental retard with statement no min impairm behav |  |
| GP | EVENT_CD = 'Eu711' -- [X]Mod mental retard sig impairment behav req attent/treatmt |  |
| GP | EVENT_CD = 'Eu71y' -- [X]Mod retard oth behav impair |  |
| GP | EVENT_CD = 'Eu71z' -- [X]Mod mental retardation without mention impairment behav |  |
| GP | EVENT_CD = 'Eu72.' -- [X]Severe mental retardation |  |
| GP | EVENT_CD = 'Eu720' -- [X]Sev mental retard with statement no min impairm behav |  |
| GP | EVENT_CD = 'Eu721' -- [X]Sev mental retard sig impairment behav req attent/treatmt |  |
| GP | EVENT_CD = 'Eu72y' -- [X]Severe mental retardation, other impairments of behaviour |  |
| GP | EVENT_CD = 'Eu72z' -- [X]Sev mental retardation without mention impairment behav |  |
| GP | EVENT_CD = 'Eu73.' -- [X]Profound mental retardation |  |
| GP | EVENT_CD = 'Eu730' -- [X]Profound ment retrd wth statement no min impairm behav |  |
| GP | EVENT_CD = 'Eu731' -- [X]Profound ment retard sig impairmnt behav req attent/treat |  |
| GP | EVENT_CD = 'Eu73y' -- [X]Profound mental retardation, other impairments of behavr |  |
| GP | EVENT_CD = 'Eu73z' -- [X]Prfnd mental retardation without mention impairment behav |  |
| GP | EVENT_CD = 'Eu7y.' -- [X]Other mental retardation |  |
| GP | EVENT_CD = 'Eu7y0' -- [X]Oth mental retard with statement no min impairm behav |  |
| GP | EVENT_CD = 'Eu7y1' -- [X]Oth mental retard sig impairment behav req attent/treatmt |  |
| GP | EVENT_CD = 'Eu7yy' -- [X]Other mental retardation, other impairments of behaviour |  |
| GP | EVENT_CD = 'Eu7yz' -- [X]Other mental retardation without mention impairment behav |  |
| GP | EVENT_CD = 'Eu7z.' -- [X]Unspecified mental retardation |  |
| GP | EVENT_CD = 'Eu7z0' -- [X]Unsp mental retard with statement no min impairm behav |  |
| GP | EVENT_CD = 'Eu7z1' -- [X]Unsp mentl retard sig impairment behav req attent/treatmt |  |
| GP | EVENT_CD = 'Eu7zy' -- [X]Unspecified mental retardatn, other impairments of behav |  |
| GP | EVENT_CD = 'Eu7zz' -- [X]Unsp mental retardation without mention impairment behav |  |
| GP | EVENT_CD = 'Eu814' -- [X]Moderate learning disability |  |
| GP | EVENT_CD = 'Eu815' -- [X]Severe learning disability |  |
| GP | EVENT_CD = 'Eu816' -- [X]Mild learning disability |  |
| GP | EVENT_CD = 'Eu817' -- [X]Profound learning disability |  |
| GP | EVENT_CD = 'Eu81z' -- [X]Developmental disorder of scholastic skills, unspecified |  |
| GP | EVENT_CD = 'ZV400' -- [V]Problems with learning |  |
| GP | EVENT_CD = 'ZV4H6' -- [V]Lack of learning and play experience |  |
| EDUCATION | SENTYPE = 'MLD' |  |
| EDUCATION | SENTYPE = 'MSI' |  |
| EDUCATION | SENTYPE = 'PMLD' |  |
| EDUCATION | SENTYPE = 'SLD' |  |

Autism

| **DATABASE** | **CODES** |
| --- | --- |
| GP | '1J9..', -- Suspected autism |
| GP | 'E140.', -- Infantile autism |
| GP | 'E1400', -- Active infantile autism |
| GP | 'E1401', -- Residual infantile autism |
| GP | 'E140z', -- Infantile autism NOS |
| GP | 'Eu840', -- [X]Childhood autism |
| GP | 'Eu841' -- [X]Atypical autism |
|  |  |
| EDUCATION | SENTYPE = 'ASD' |
| Congenital abnormalities | SYNDROME_ICD = 'F84.0' |
| Congenital abnormalities | SYNDROME_ICD = 'F84.5' |
| Congenital abnormalities | SYNDROME_ICD_2 = 'F84.0' |
| Congenital abnormalities | SYNDROME_ICD_2 = 'F84.5' |
| Congenital abnormalities | ANOMALY_CD = 'F84.0' |
| Congenital abnormalities | ANOMALY_CD = 'F84.5' |
| HOSPITAL ADMSSION | DIAG_CD_1234 = 'F845' -- Asperger's syndrome |
| HOSPITAL ADMISSION | 'F84%' -- AUTISM |
| HOSPITAL ADMISSION | DIAG_CD_1234 = 'F841' -- Atypical autism |
|  |  |

Codes for antipsychotic medication:

| **DATABASE** | **CODES** |
| --- | --- |
| GP | EVENT_CD = 'd4…' -- ANTIPSYCHOTIC DRUGS |
| GP | EVENT_CD = 'd4f..' -- SULPIRIDE |
| GP | EVENT_CD = 'd4f1.' -- DOLMATIL 200mg tablets |
| GP | EVENT_CD = 'd4f2.' -- *SULPITIL 200mg tablets x28CP |
| GP | EVENT_CD = 'd4f3.' -- *SULPITIL 200mg tablets x112CP |
| GP | EVENT_CD = 'd4f4.' -- *SULPAREX 200mg tablets |
| GP | EVENT_CD = 'd4f5.' -- DOLMATIL 400mg tablets |
| GP | EVENT_CD = 'd4f6.' -- SULP200mg/5mL oral solution |
| GP | EVENT_CD = 'd4fw.' -- SULPIRIDE 200mg/5mL oral solution |
| GP | EVENT_CD = 'd4fx.' -- SULPIRIDE 400mg tablets |
| GP | EVENT_CD = 'd4fy.' -- SULPIRIDE 200mg/5mL sugar free solution |
| GP | EVENT_CD = 'd4fz.' -- SULPIRIDE 200mg tablets |
| GP | EVENT_CD = 'd41..' -- CHLORPROMAZINE HYDROCHLORIDE |
| GP | EVENT_CD = 'd411.' -- CHLORPROMAZINE 10mg tablets |
| GP | EVENT_CD = 'd412.' -- CHLORPROMAZINE 25mg tablets |
| GP | EVENT_CD = 'd413.' -- CHLORPROMAZINE 50mg tablets |
| GP | EVENT_CD = 'd414.' -- CHLORPROMAZINE 100mg tablets |
| GP | EVENT_CD = 'd415.' -- CHLORPROMAZINE 25mg/5mL syrup |
| GP | EVENT_CD = 'd416.' -- CHLORACTIL 25mg tablets |
| GP | EVENT_CD = 'd417.' -- CHLORACTIL 50mg tablets |
| GP | EVENT_CD = 'd418.' -- CHLORACTIL 100mg tablets |
| GP | EVENT_CD = 'd419.' -- *DOZINE 25mg/5mL syrup |
| GP | EVENT_CD = 'd41A.' -- CHLORPROMAZINE 25mg/5mL sugar free solution |
| GP | EVENT_CD = 'd41B.' -- CHLORPROMAZINE 100mg/5mL sugar free solution |
| GP | EVENT_CD = 'd41a.' -- *LARGACTIL 10mg tablets |
| GP | EVENT_CD = 'd41b.' -- *LARGACTIL 25mg tablets |
| GP | EVENT_CD = 'd41c.' -- *LARGACTIL 50mg tablets |
| GP | EVENT_CD = 'd41d.' -- *LARGACTIL 100mg tablets |
| GP | EVENT_CD = 'd41e.' -- *LARGACTIL 25mg/5mL syrup |
| GP | EVENT_CD = 'd41f.' -- LARGACTIL FORTE 100mg/5mL syrup |
| GP | EVENT_CD = 'd41g.' -- *LARGACTIL 25mg/mL injection |
| GP | EVENT_CD = 'd41h.' -- LARGACTIL [CNS] 50mg/2mL injection |
| GP | EVENT_CD = 'd41i.' -- *LARGACTIL 100mg suppositories |
| GP | EVENT_CD = 'd41j.' -- CHLORPROMAZINE 100mg/5mL sugar free suspension |
| GP | EVENT_CD = 'd41k.' -- CHLORPROMAZINE 100mg suppositories |
| GP | EVENT_CD = 'd41l.' -- CHLORPROMAZINE 25mg/1mL injection |
| GP | EVENT_CD = 'd41m.' -- CHLORPROMAZINE 50mg/2mL injection |
| GP | EVENT_CD = 'd41o.' -- CHLORPROMAZINE 100mg/5mL syrup |
| GP | EVENT_CD = 'd42..' -- BENPERIDOL |
| GP | EVENT_CD = 'd421.' -- ANQUIL 250micrograms tablets |
| GP | EVENT_CD = 'd422.' -- *BENQUIL 250micrograms tablets |
| GP | EVENT_CD = 'd42z.' -- BENPERIDOL 250microgram tablets |
| GP | EVENT_CD = 'd43..' -- *CHLORPROTHIXENE |
| GP | EVENT_CD = 'd431.' -- *TARACTAN 15mg tablets |
| GP | EVENT_CD = 'd432.' -- *TARACTAN 50mg tablets |
| GP | EVENT_CD = 'd43y.' -- *CHLORPROTHIXENE 15mg tablets |
| GP | EVENT_CD = 'd43z.' -- *CHLORPROTHIXENE 50mg tablets |
| GP | EVENT_CD = 'd44..' -- DROPERIDOL [CENTRAL NERVOUS SYSTEM USE] |
| GP | EVENT_CD = 'd441.' -- *DROLEPTAN 10mg tablets |
| GP | EVENT_CD = 'd442.' -- *DROLEPTAN 1mg/mL oral liquid |
| GP | EVENT_CD = 'd443.' -- *DROLEPTAN 10mg/2mL injection |
| GP | EVENT_CD = 'd444.' -- XOMOLIX 2.5mg/1mL solution finjection |
| GP | EVENT_CD = 'd44w.' -- DROPERIDOL 2.5mg/1mL solution finjection |
| GP | EVENT_CD = 'd44x.' -- *DROPERIDOL 10mg tablets |
| GP | EVENT_CD = 'd44y.' -- *DROPERIDOL 1mg/mL oral liquid |
| GP | EVENT_CD = 'd44z.' -- *DROPERIDOL 10mg/2mL injection |
| GP | EVENT_CD = 'd45..' -- FLUPENTIXOL [ANTIPSYCHOTIC] |
| GP | EVENT_CD = 'd451.' -- DEPIXOL 3mg tablets |
| GP | EVENT_CD = 'd45z.' -- FLUPENTIXOL 3mg tablets |
| GP | EVENT_CD = 'd46..' -- FLUPHENAZINE HYDROCHLORIDE |
| GP | EVENT_CD = 'd461.' -- *MODITEN 1mg tablets |
| GP | EVENT_CD = 'd462.' -- *MODITEN 2.5mg tablets |
| GP | EVENT_CD = 'd463.' -- *MODITEN 5mg tablets |
| GP | EVENT_CD = 'd46x.' -- FLUPHENAZINE HYDROCHLORIDE 1mg tablets |
| GP | EVENT_CD = 'd46y.' -- FLUPHENAZINE HYDROCHLORIDE 2.5mg tablets |
| GP | EVENT_CD = 'd46z.' -- FLUPHENAZINE HYDROCHLORIDE 5mg tablets |
| GP | EVENT_CD = 'd47..' -- HALOPERIDOL [ANTIPSYCHOTIC] |
| GP | EVENT_CD = 'd471.' -- HALOPERIDOL 1.5mg tablets |
| GP | EVENT_CD = 'd472.' -- HALOPERIDOL 5mg tablets |
| GP | EVENT_CD = 'd473.' -- HALOPERIDOL 10mg tablets |
| GP | EVENT_CD = 'd474.' -- HALOPERIDOL 20mg tablets |
| GP | EVENT_CD = 'd475.' -- HALOPERIDOL 2mg/mL liquid |
| GP | EVENT_CD = 'd476.' -- DOZIC 1mg/mL liquid |
| GP | EVENT_CD = 'd477.' -- *DOZIC 2mg/mL liquid |
| GP | EVENT_CD = 'd478.' -- FORTUNAN 500micrograms tablets |
| GP | EVENT_CD = 'd479.' -- *FORTUNAN 1.5mg tablets |
| GP | EVENT_CD = 'd47A.' -- HALOPERIDOL 2mg/5mL sugar free solution |
| GP | EVENT_CD = 'd47B.' -- HALOPERIDOL 1mg/5mL sugar free solution |
| GP | EVENT_CD = 'd47C.' -- KENTACE 1.5mg tablets |
| GP | EVENT_CD = 'd47D.' -- KENTACE 5mg tablets |
| GP | EVENT_CD = 'd47E.' -- KENTACE 10mg tablets |
| GP | EVENT_CD = 'd47F.' -- KENTACE 20mg tablets |
| GP | EVENT_CD = 'd47a.' -- *FORTUNAN 5mg tablets |
| GP | EVENT_CD = 'd47b.' -- *FORTUNAN 10mg tablets |
| GP | EVENT_CD = 'd47c.' -- *FORTUNAN 20mg tablets |
| GP | EVENT_CD = 'd47d.' -- HALDOL 5mg tablets |
| GP | EVENT_CD = 'd47e.' -- HALDOL 10mg tablets |
| GP | EVENT_CD = 'd47f.' -- HALDOL 2mg/mL liquid |
| GP | EVENT_CD = 'd47g.' -- *HALDOL 10mg/mL liquid |
| GP | EVENT_CD = 'd47h.' -- HALDOL 5mg/1mL injection |
| GP | EVENT_CD = 'd47i.' -- *HALDOL 10mg/2mL injection |
| GP | EVENT_CD = 'd47j.' -- SERENACE 500micrograms capsules |
| GP | EVENT_CD = 'd47k.' -- SERENACE 1.5mg tablets |
| GP | EVENT_CD = 'd47l.' -- SERENACE 5mg tablets |
| GP | EVENT_CD = 'd47m.' -- SERENACE 10mg tablets |
| GP | EVENT_CD = 'd47n.' -- SERENACE 20mg tablets |
| GP | EVENT_CD = 'd47o.' -- SERENACE 2mg/mL liquid 100mL |
| GP | EVENT_CD = 'd47p.' -- SERENACE 5mg/1mL injection |
| GP | EVENT_CD = 'd47q.' -- SERENACE 20mg/2mL injection |
| GP | EVENT_CD = 'd47r.' -- HALOPERIDOL 500microgram capsules |
| GP | EVENT_CD = 'd47s.' -- SERENACE 2mg/mL liquid 500mL |
| GP | EVENT_CD = 'd47t.' -- HALOPERIDOL 1mg/mL liquid |
| GP | EVENT_CD = 'd47u.' -- HALOPERIDOL 500micrograms tablets |
| GP | EVENT_CD = 'd47v.' -- HALOPERIDOL 5mg/1mL injection |
| GP | EVENT_CD = 'd47w.' -- HALOPERIDOL 10mg/2mL injection |
| GP | EVENT_CD = 'd47x.' -- HALOPERIDOL 20mg/2mL injection |
| GP | EVENT_CD = 'd47y.' -- HALOPERIDOL 10mg/mL oral solution |
| GP | EVENT_CD = 'd48..' -- LEVOMEPROMAZINE |
| GP | EVENT_CD = 'd481.' -- NOZINAN 25mg/1mL injection |
| GP | EVENT_CD = 'd482.' -- *VERACTIL 25mg tablets |
| GP | EVENT_CD = 'd483.' -- NOZINAN 25mg tablets |
| GP | EVENT_CD = 'd48y.' -- LEVOMEPROMAZINE 25mg/1mL injection |
| GP | EVENT_CD = 'd48z.' -- LEVOMEPROMAZINE 25mg tablets |
| GP | EVENT_CD = 'd49..' -- OXYPERTINE |
| GP | EVENT_CD = 'd491.' -- *INTEGRIN 10mg capsules |
| GP | EVENT_CD = 'd492.' -- *INTEGRIN 40mg tablets |
| GP | EVENT_CD = 'd49y.' -- *OXYPERTINE 10mg capsules |
| GP | EVENT_CD = 'd49z.' -- *OXYPERTINE 40mg tablets |
| GP | EVENT_CD = 'd4a..' -- PERICYAZINE |
| GP | EVENT_CD = 'd4a1.' -- *NEULACTIL 2.5mg tablets |
| GP | EVENT_CD = 'd4a2.' -- *NEULACTIL 10mg tablets |
| GP | EVENT_CD = 'd4a3.' -- *NEULACTIL 25mg tablets |
| GP | EVENT_CD = 'd4a4.' -- *NEULACTIL FORTE 10mg/5mL syrp |
| GP | EVENT_CD = 'd4aw.' -- PERICYAZINE 2.5mg tablets |
| GP | EVENT_CD = 'd4ax.' -- PERICYAZINE 10mg tablets |
| GP | EVENT_CD = 'd4ay.' -- *PERICYAZINE 25mg tablets |
| GP | EVENT_CD = 'd4az.' -- PERICYAZINE 10mg/5mL syrup |
| GP | EVENT_CD = 'd4b..' -- PERPHENAZINE [CENTRAL NERVOUS SYSTEM USE] |
| GP | EVENT_CD = 'd4b1.' -- FENTAZIN 2mg tablets |
| GP | EVENT_CD = 'd4b2.' -- FENTAZIN 4mg tablets |
| GP | EVENT_CD = 'd4b3.' -- *FENTAZIN 8mg tablets |
| GP | EVENT_CD = 'd4b4.' -- *FENTAZIN 5mg/1mL injection |
| GP | EVENT_CD = 'd4b5.' -- PERPHENAZINE 2mg/5mL sugar free solution |
| GP | EVENT_CD = 'd4b6.' -- PERPHENAZINE 4mg/5mL sugar free solution |
| GP | EVENT_CD = 'd4bx.' -- PERPHENAZINE 2mg tablets |
| GP | EVENT_CD = 'd4by.' -- PERPHENAZINE 4mg tablets |
| GP | EVENT_CD = 'd4bz.' -- *PERPHENAZINE 8mg tablets |
| GP | EVENT_CD = 'd4c..' -- PIMOZIDE |
| GP | EVENT_CD = 'd4c1.' -- *ORAP 2mg tablets |
| GP | EVENT_CD = 'd4c2.' -- ORAP 4mg tablets |
| GP | EVENT_CD = 'd4c3.' -- *ORAP 10mg tablets |
| GP | EVENT_CD = 'd4cx.' -- *PIMOZIDE 2mg tablets |
| GP | EVENT_CD = 'd4cy.' -- PIMOZIDE 4mg tablets |
| GP | EVENT_CD = 'd4cz.' -- *PIMOZIDE 10mg tablets |
| GP | EVENT_CD = 'd4d..' -- PROCHLORPERAZINE [antipsych] [see dhe..] |
| GP | EVENT_CD = 'd4e..' -- PROMAZINE HYDROCHLORIDE |
| GP | EVENT_CD = 'd4e1.' -- *SPARINE 50mg/5mL suspension |
| GP | EVENT_CD = 'd4e2.' -- *SPARINE 50mg/1mL injection |
| GP | EVENT_CD = 'd4e3.' -- *SPARINE 100mg/2mL injection |
| GP | EVENT_CD = 'd4e4.' -- PROMAZINE 25mg tablets |
| GP | EVENT_CD = 'd4e5.' -- PROMAZINE 50mg tablets |
| GP | EVENT_CD = 'd4ev.' -- PROMAZINE 25mg/5mL syrup |
| GP | EVENT_CD = 'd4ew.' -- PROMAZINE 50mg/5mL syrup |
| GP | EVENT_CD = 'd4ex.' -- *PROMAZINE 50mg/5mL suspension |
| GP | EVENT_CD = 'd4ey.' -- PROMAZINE 50mg/1mL injection |
| GP | EVENT_CD = 'd4ez.' -- *PROMAZINE 100mg/2mL injection |
| GP | EVENT_CD = 'd4g..' -- THIORIDAZINE |
| GP | EVENT_CD = 'd4g1.' -- *MELLERIL 10mg tablets |
| GP | EVENT_CD = 'd4g2.' -- *MELLERIL 25mg tablets |
| GP | EVENT_CD = 'd4g3.' -- *MELLERIL 50mg tablets |
| GP | EVENT_CD = 'd4g4.' -- *MELLERIL 100mg tablets |
| GP | EVENT_CD = 'd4g5.' -- *MELLERIL 25mg/5mL suspension |
| GP | EVENT_CD = 'd4g6.' -- MELLERIL 100mg/5mL oral suspension |
| GP | EVENT_CD = 'd4g7.' -- MELLERIL 25mg/5mL orange syrup |
| GP | EVENT_CD = 'd4gp.' -- THIORIDAZINE 10mg/5mL syrup |
| GP | EVENT_CD = 'd4gq.' -- THIORIDAZINE 25mg/5mL sugar free solution |
| GP | EVENT_CD = 'd4gr.' -- THIORIDAZINE 50mg/5mL sugar free solution |
| GP | EVENT_CD = 'd4gs.' -- THIORIDAZINE 100mg/5mL sugar free solution |
| GP | EVENT_CD = 'd4gt.' -- *THIORIDAZINE 10mg tablets |
| GP | EVENT_CD = 'd4gu.' -- THIORIDAZINE 25mg tablets |
| GP | EVENT_CD = 'd4gv.' -- THIORIDAZINE 50mg tablets |
| GP | EVENT_CD = 'd4gw.' -- THIORIDAZINE 100mg tablets |
| GP | EVENT_CD = 'd4gx.' -- THIORIDAZINE 25mg/5mL suspension |
| GP | EVENT_CD = 'd4gy.' -- THIORIDAZINE 100mg/5mL oral suspension |
| GP | EVENT_CD = 'd4gz.' -- *THIORIDAZINE 25mg/5mL syrup |
| GP | EVENT_CD = 'd4h..' -- TRIFLUOPERAZINE [ANTIPSYCHOTIC] |
| GP | EVENT_CD = 'd4h1.' -- STELAZINE 1mg tablets |
| GP | EVENT_CD = 'd4h2.' -- STELAZINE 5mg tablets |
| GP | EVENT_CD = 'd4h3.' -- *STELAZINE 2mg m/r capsules |
| GP | EVENT_CD = 'd4h4.' -- *STELAZINE 10mg m/r capsules |
| GP | EVENT_CD = 'd4h5.' -- *STELAZINE 15mg m/r capsules |
| GP | EVENT_CD = 'd4h6.' -- STELAZINE 1mg/5mL syrup |
| GP | EVENT_CD = 'd4h7.' -- STELAZINE CONCENTRATE 10mg/mL liquid |
| GP | EVENT_CD = 'd4h8.' -- *STELAZINE 1mg/1mL injection |
| GP | EVENT_CD = 'd4h9.' -- TRIFLUOPERAZINE 5mg/5mL sugar free syrup |
| GP | EVENT_CD = 'd4hA.' -- STELAZINE FORTE 5mg/5mL sugar free oral suspension |
| GP | EVENT_CD = 'd4hr.' -- TRIFLUOPERAZINE 5mg/5mL sugar free oral suspension |
| GP | EVENT_CD = 'd4hs.' -- TRIFLUOPERAZINE 1mg tablets |
| GP | EVENT_CD = 'd4ht.' -- TRIFLUOPERAZINE 5mg tablets |
| GP | EVENT_CD = 'd4hu.' -- *TRIFLUOPERAZINE 2mg m/r caps |
| GP | EVENT_CD = 'd4hv.' -- *TRIFLUOPERAZINE 10mg m/r caps |
| GP | EVENT_CD = 'd4hw.' -- *TRIFLUOPERAZINE 15mg m/r caps |
| GP | EVENT_CD = 'd4hx.' -- TRIFLUOPERAZINE 1mg/5mL syrup |
| GP | EVENT_CD = 'd4hy.' -- TRIFLUOPERAZINE 10mg/mL liquid |
| GP | EVENT_CD = 'd4hz.' -- TRIFLUOPERAZINE 1mg/1mL injection |
| GP | EVENT_CD = 'd4i1.' -- TRIFLUPERIDOL |
| GP | EVENT_CD = 'd4i2.' -- TRIPERIDOL 500micrograms tablets |
| GP | EVENT_CD = 'd4iy.' -- *TRIPERIDOL 1mg tablets |
| GP | EVENT_CD = 'd4iz.' -- TRIFLUPERIDOL 500microgram tablets |
| GP | EVENT_CD = 'd4l..' -- *TRIFLUPERIDOL 1mg tablets |
| GP | EVENT_CD = 'd4j..' -- ZUCLOPENTHIXOL DIHYDROCHLORIDE |
| GP | EVENT_CD = 'd4j1.' -- CLOPIXOL 2mg tablets |
| GP | EVENT_CD = 'd4j2.' -- CLOPIXOL 10mg tablets |
| GP | EVENT_CD = 'd4j3.' -- CLOPIXOL 25mg tablets |
| GP | EVENT_CD = 'd4jx.' -- ZUCLOPENTHIXOL DIHYDROCHLORIDE 2mg tablets |
| GP | EVENT_CD = 'd4jy.' -- ZUCLOPENTHIXOL DIHYDROCHLORIDE 10mg tablets |
| GP | EVENT_CD = 'd4jz.' -- ZUCLOPENTHIXOL DIHYDROCHLORIDE 25mg tablets |
| GP | EVENT_CD = 'd4k..' -- LOXAPINE SUCCINATE |
| GP | EVENT_CD = 'd4k1.' -- *LOXAPINE 10mg capsules |
| GP | EVENT_CD = 'd4k2.' -- *LOXAPINE 25mg capsules |
| GP | EVENT_CD = 'd4k3.' -- *LOXAPINE 50mg capsules |
| GP | EVENT_CD = 'd4k4.' -- *LOXAPAC 10mg capsules |
| GP | EVENT_CD = 'd4k5.' -- *LOXAPAC 25mg capsules |
| GP | EVENT_CD = 'd4k6.' -- *LOXAPAC 50mg capsules |
| GP | EVENT_CD = 'd4n..' -- ZUCLOPENTHIXOL ACETATE |
| GP | EVENT_CD = 'd4n1.' -- CLOPIXOL ACUPHASE 50mg/1mL injection (oily) |
| GP | EVENT_CD = 'd4n2.' -- CLOPIXOL ACUPHASE 100mg/2mL injection (oily) |
| GP | EVENT_CD = 'd4n3.' -- ZUCLOPENTHIXOL ACETATE 50mg/1mL injection (oily) |
| GP | EVENT_CD = 'd4n4.' -- ZUCLOPENTHIXOL ACETATE 100mg/2mL injection (oily) |
| GP | EVENT_CD = 'd5...' -- ANTIPSYCHOTIC DEPOT INJECTIONS |
| GP | EVENT_CD = 'd51..' -- FLUPENTIXOL DECANOATE |
| GP | EVENT_CD = 'd511.' -- DEPIXOL 20mg/1mL injection |
| GP | EVENT_CD = 'd512.' -- *DEPIXOL 20mg/1mL syringe |
| GP | EVENT_CD = 'd513.' -- DEPIXOL 40mg/2mL injection |
| GP | EVENT_CD = 'd514.' -- *DEPIXOL 40mg/2mL syringe |
| GP | EVENT_CD = 'd515.' -- *DEPIXOL 200mg/10mL injection |
| GP | EVENT_CD = 'd516.' -- DEPIXOL CONC. 100mg/1mL injection |
| GP | EVENT_CD = 'd517.' -- DEPIXOL CONC. 500mg/5mL injection |
| GP | EVENT_CD = 'd518.' -- DEPIXOL CONC. 50mg/0.5mL injection |
| GP | EVENT_CD = 'd519.' -- FLUPENTIXOL 50mg/0.5mL injection |
| GP | EVENT_CD = 'd51a.' -- DEPIXOL LOW VOLUME 200mg/1mL intramuscular injection |
| GP | EVENT_CD = 'd51s.' -- FLUPENTHIXOL DECANOATE 20mg/1mL prefilled syringe |
| GP | EVENT_CD = 'd51t.' -- FLUPENTHIXOL DECANOATE 40mg/2mL prefilled syringe |
| GP | EVENT_CD = 'd51u.' -- FLUPENTIXOL DECANOATE 200mg/1mL intramuscular injection |
| GP | EVENT_CD = 'd51v.' -- FLUPENTIXOL DECANOATE 20mg/1mL injection |
| GP | EVENT_CD = 'd51w.' -- FLUPENTIXOL DECANOATE 40mg/2mL injection |
| GP | EVENT_CD = 'd51x.' -- FLUPENTHIXOL DECANOATE 200mg/10mL injection |
| GP | EVENT_CD = 'd51y.' -- FLUPENTIXOL DECANOATE 100mg/1mL injection |
| GP | EVENT_CD = 'd51z.' -- FLUPENTHIXOL DECANOATE 500mg/5mL injection |
| GP | EVENT_CD = 'd52..' -- FLUPHENAZINE DECANOATE |
| GP | EVENT_CD = 'd521.' -- MODECATE 12.5mg/0.5mL injection |
| GP | EVENT_CD = 'd522.' -- MODECATE 25mg/1mL injection |
| GP | EVENT_CD = 'd523.' -- *MODECATE 25mg/1mL syringe |
| GP | EVENT_CD = 'd524.' -- MODECATE 50mg/2mL injection |
| GP | EVENT_CD = 'd525.' -- *MODECATE 50mg/2mL syringe |
| GP | EVENT_CD = 'd526.' -- *MODECATE 250mg/10mL injection |
| GP | EVENT_CD = 'd527.' -- MODECATE CONCENTRATE 50mg/0.5mL injection |
| GP | EVENT_CD = 'd528.' -- MODECATE CONCENTRATE 100mg/1mL injection |
| GP | EVENT_CD = 'd529.' -- FLUPHENAZINE DECANOATE 50mg/0.5mL injection |
| GP | EVENT_CD = 'd52A.' -- *DECAZATE 25mg/1mL injection |
| GP | EVENT_CD = 'd52B.' -- *DECAZATE 50mg/0.5mL injection |
| GP | EVENT_CD = 'd52C.' -- *DECAZATE 100mg/1mL injection |
| GP | EVENT_CD = 'd52a.' -- FLUPHENAZINE DECANOATE 100mg/1mL injection |
| GP | EVENT_CD = 'd52s.' -- FLUPHENAZINE DECANOATE 25mg/1mL prefilled syringe |
| GP | EVENT_CD = 'd52t.' -- FLUPHENAZINE DECANOATE 50mg/2mL prefilled syringe |
| GP | EVENT_CD = 'd52u.' -- FLUPHENAZINE DECANOATE 12.5mg/0.5mL injection |
| GP | EVENT_CD = 'd52v.' -- FLUPHENAZINE DECANOATE 25mg/1mL injection |
| GP | EVENT_CD = 'd52w.' -- FLUPHENAZINE DECANOATE 50mg/2mL injection |
| GP | EVENT_CD = 'd52x.' -- FLUPHENAZINE DECANOATE 250mg/10mL injection |
| GP | EVENT_CD = 'd53..' -- *FLUPHENAZINE ENANTHATE |
| GP | EVENT_CD = 'd531.' -- MODITEN ENANTHATE 25mg/1mL injection |
| GP | EVENT_CD = 'd532.' -- FLUPHENAZINE ENANTHATE 25mg/1mL injection |
| GP | EVENT_CD = 'd54..' -- FLUSPIRILENE |
| GP | EVENT_CD = 'd541.' -- *REDEPTIN 2mg/1mL injection |
| GP | EVENT_CD = 'd542.' -- *REDEPTIN 6mg/3mL injection |
| GP | EVENT_CD = 'd543.' -- *REDEPTIN 12mg/6mL injection |
| GP | EVENT_CD = 'd544.' -- FLUSPIRILENE 2mg/1mL injection |
| GP | EVENT_CD = 'd545.' -- FLUSPIRILENE 6mg/3mL injection |
| GP | EVENT_CD = 'd546.' -- FLUSPIRILENE 12mg/6mL injection |
| GP | EVENT_CD = 'd55..' -- HALOPERIDOL DECANOATE |
| GP | EVENT_CD = 'd551.' -- HALDOL DECANOATE 50mg/1mL injection |
| GP | EVENT_CD = 'd552.' -- HALDOL DECANOATE 100mg/1mL injection |
| GP | EVENT_CD = 'd553.' -- HALOPERIDOL 50mg/1mL injection |
| GP | EVENT_CD = 'd554.' -- HALOPERIDOL 100mg/1mL injection |
| GP | EVENT_CD = 'd56..' -- PIPOTIAZINE PALMITATE |
| GP | EVENT_CD = 'd561.' -- PIPORTIL DEPOT 50mg/1mL injection |
| GP | EVENT_CD = 'd562.' -- PIPORTIL DEPOT 100mg/2mL injection |
| GP | EVENT_CD = 'd563.' -- PIPOTIAZINE 50mg/1mL injection |
| GP | EVENT_CD = 'd564.' -- PIPOTIAZINE 100mg/2mL injection |
| GP | EVENT_CD = 'd57..' -- ZUCLOPENTHIXOL DECANOATE |
| GP | EVENT_CD = 'd571.' -- CLOPIXOL 200mg/1mL injection |
| GP | EVENT_CD = 'd572.' -- *CLOPIXOL 2g/10mL injection |
| GP | EVENT_CD = 'd573.' -- CLOPIXOL CONC. 500mg/1mL injection |
| GP | EVENT_CD = 'd574.' -- CLOPIXOL ACUPHASE 50mg/1mL injection (oily) |
| GP | EVENT_CD = 'd575.' -- CLOPIXOL ACUPHASE 100mg/2mL injection (oily) |
| GP | EVENT_CD = 'd576.' -- ZUCLOPENTHIXOL DECANOATE 200mg/1mL injection |
| GP | EVENT_CD = 'd577.' -- ZUCLOPENTHIXOL DECANOATE 50mg/1mL injection |
| GP | EVENT_CD = 'd578.' -- ZUCLOPENTHIXOL DECANOATE 100mg/2mL injection |
| GP | EVENT_CD = 'd57y.' -- ZUCLOPENTHIXOL DECANOATE 2g/10mL injection |
| GP | EVENT_CD = 'd57z.' -- ZUCLOPENTHIXOL DECANOATE 500mg/1mL injection |
| GP | EVENT_CD = 'd4l..' -- CLOZAPINE |
| GP | EVENT_CD = 'd4l1.' -- CLOZAPINE 25mg tablets |
| GP | EVENT_CD = 'd4l2.' -- CLOZAPINE 100mg tablets |
| GP | EVENT_CD = 'd4l3.' -- CLOZARIL 25mg tablets x84CP |
| GP | EVENT_CD = 'd4l4.' -- CLOZARIL 100mg tablets x84CP |
| GP | EVENT_CD = 'd4l5.' -- CLOZARIL COMMUNITY PACK 25mg tablets x28CP |
| GP | EVENT_CD = 'd4l6.' -- CLOZARIL COMMUNITY PACK 100mg tablets x28CP |
| GP | EVENT_CD = 'd4l7.' -- DENZAPINE 25mg tablets |
| GP | EVENT_CD = 'd4l8.' -- DENZAPINE 100mg tablets |
| GP | EVENT_CD = 'd4l9.' -- ZAPONEX 25mg tablets |
| GP | EVENT_CD = 'd4lA.' -- ZAPONEX 100mg tablets |
| GP | EVENT_CD = 'd4lB.' -- DENZAPINE 50mg/mL oral suspension 100mL |
| GP | EVENT_CD = 'd4lC.' -- CLOZAPINE 50mg/mL oral suspension |
| GP | EVENT_CD = 'd4lD.' -- DENZAPINE 50mg tablets |
| GP | EVENT_CD = 'd4lE.' -- CLOZAPINE 50mg tablets |
| GP | EVENT_CD = 'd4lF.' -- DENZAPINE 200mg tablets |
| GP | EVENT_CD = 'd4lG.' -- CLOZAPINE 200mg tablets |
| GP | EVENT_CD = 'd4m..' -- REMOXIPRIDE |
| GP | EVENT_CD = 'd4m1.' -- REMOXIPRIDE 150mg m/r capsules |
| GP | EVENT_CD = 'd4m2.' -- REMOXIPRIDE 300mg m/r capsules |
| GP | EVENT_CD = 'd4m3.' -- *ROXIAM 150mg m/r capsules |
| GP | EVENT_CD = 'd4m4.' -- *ROXIAM 300mg m/r capsules |
| GP | EVENT_CD = 'd4p..' -- RISPERIDONE |
| GP | EVENT_CD = 'd4p1.' -- RISPERIDONE 1mg tablets |
| GP | EVENT_CD = 'd4p2.' -- RISPERIDONE 2mg tablets |
| GP | EVENT_CD = 'd4p3.' -- RISPERIDONE 3mg tablets |
| GP | EVENT_CD = 'd4p4.' -- RISPERIDONE 4mg tablets |
| GP | EVENT_CD = 'd4p5.' -- RISPERDAL 1mg tablets |
| GP | EVENT_CD = 'd4p6.' -- RISPERDAL 2mg tablets |
| GP | EVENT_CD = 'd4p7.' -- RISPERDAL 3mg tablets |
| GP | EVENT_CD = 'd4p8.' -- RISPERDAL 4mg tablets |
| GP | EVENT_CD = 'd4p9.' -- RISPERIDONE 1mg/mL liquid |
| GP | EVENT_CD = 'd4pA.' -- RISPERDAL 1mg/mL liquid |
| GP | EVENT_CD = 'd4pB.' -- RISPERIDONE 6mg tablets |
| GP | EVENT_CD = 'd4pC.' -- RISPERDAL 6mg tablets |
| GP | EVENT_CD = 'd4pD.' -- RISPERDAL 0.5mg tablets |
| GP | EVENT_CD = 'd4pE.' -- RISPERDAL CONSTA 25mg powder+solvent fsuspension finjection |
| GP | EVENT_CD = 'd4pF.' -- RISPERDAL CONSTA 37.5mg powder+solvent fsuspension finjection |
| GP | EVENT_CD = 'd4pG.' -- RISPERDAL CONSTA 50mg powder+solvent fsuspension finjection |
| GP | EVENT_CD = 'd4pH.' -- RISPERIDONE 1mg oro-dispersible tablets |
| GP | EVENT_CD = 'd4pJ.' -- RISPERIDONE 2mg oro-dispersible tablets |
| GP | EVENT_CD = 'd4pK.' -- RISPERDAL QUICKLET 1mg oro-dispersible tablets |
| GP | EVENT_CD = 'd4pL.' -- RISPERDAL QUICKLET 2mg oro-dispersible tablets |
| GP | EVENT_CD = 'd4pM.' -- RISPERIDONE 0.5mg oro-dispersible tablets |
| GP | EVENT_CD = 'd4pN.' -- RISPERDAL QUICKLET 0.5mg oro-dispersible tablets |
| GP | EVENT_CD = 'd4pO.' -- RISPERDAL QUICKLET 3mg oro-dispersible tablets |
| GP | EVENT_CD = 'd4pP.' -- RISPERDAL QUICKLET 4mg oro-dispersible tablets |
| GP | EVENT_CD = 'd4pQ.' -- RISPERIDONE 3mg oro-dispersible tablets |
| GP | EVENT_CD = 'd4pR.' -- RISPERIDONE 4mg oro-dispersible tablets |
| GP | EVENT_CD = 'd4pw.' -- RISPERIDONE 50mg powder+solvent fsuspension finjection |
| GP | EVENT_CD = 'd4px.' -- RISPERIDONE 37.5mg powder+solvent fsuspension finjection |
| GP | EVENT_CD = 'd4py.' -- RISPERIDONE 25mg powder+solvent fsuspension finjection |
| GP | EVENT_CD = 'd4pz.' -- RISPERIDONE 0.5mg tablets |
| GP | EVENT_CD = 'd4q..' -- SERTINDOLE |
| GP | EVENT_CD = 'd4q1.' -- SERTINDOLE 4mg tablets |
| GP | EVENT_CD = 'd4q2.' -- SERTINDOLE 12mg tablets |
| GP | EVENT_CD = 'd4q3.' -- SERTINDOLE 16mg tablets |
| GP | EVENT_CD = 'd4q4.' -- SERTINDOLE 20mg tablets |
| GP | EVENT_CD = 'd4q5.' -- SERDOLECT 4mg tablets |
| GP | EVENT_CD = 'd4q6.' -- SERDOLECT 12mg tablets |
| GP | EVENT_CD = 'd4q7.' -- SERDOLECT 16mg tablets |
| GP | EVENT_CD = 'd4q8.' -- SERDOLECT 20mg tablets |
| GP | EVENT_CD = 'd4r..' -- OLANZAPINE |
| GP | EVENT_CD = 'd4r1.' -- OLANZAPINE 5mg tablets |
| GP | EVENT_CD = 'd4r2.' -- OLANZAPINE 7.5mg tablets |
| GP | EVENT_CD = 'd4r3.' -- OLANZAPINE 10mg tablets |
| GP | EVENT_CD = 'd4r4.' -- ZYPREXA 5mg tablets |
| GP | EVENT_CD = 'd4r5.' -- ZYPREXA 7.5mg tablets |
| GP | EVENT_CD = 'd4r6.' -- ZYPREXA 10mg tablets |
| GP | EVENT_CD = 'd4r7.' -- OLANZAPINE 2.5mg tablets |
| GP | EVENT_CD = 'd4r8.' -- ZYPREXA 2.5mg tablets |
| GP | EVENT_CD = 'd4r9.' -- ZYPREXA VELOTAB 5mg dispersible tablets |
| GP | EVENT_CD = 'd4rA.' -- ZYPREXA VELOTAB 10mg dispersible tablets |
| GP | EVENT_CD = 'd4rB.' -- ZYPREXA 15mg tablets |
| GP | EVENT_CD = 'd4rC.' -- ZYPREXA VELOTAB 15mg dispersible tablets |
| GP | EVENT_CD = 'd4rD.' -- ZYPREXA 10mg injection (pdr frecon) |
| GP | EVENT_CD = 'd4rE.' -- ZYPREXA VELOTAB 20mg dispersible tablets |
| GP | EVENT_CD = 'd4rF.' -- ZYPREXA 20mg tablets |
| GP | EVENT_CD = 'd4rG.' -- ZALASTA 2.5mg tablets |
| GP | EVENT_CD = 'd4rH.' -- ZALASTA 5mg tablets |
| GP | EVENT_CD = 'd4rI.' -- ZALASTA 7.5mg tablets |
| GP | EVENT_CD = 'd4rJ.' -- ZALASTA 15mg tablets |
| GP | EVENT_CD = 'd4rK.' -- ZALASTA 20mg tablets |
| GP | EVENT_CD = 'd4rL.' -- ZALASTA 5mg dispersible tablets |
| GP | EVENT_CD = 'd4rM.' -- ZALASTA 10mg dispersible tablets |
| GP | EVENT_CD = 'd4rN.' -- ZALASTA 15mg dispersible tablets |
| GP | EVENT_CD = 'd4rO.' -- ZALASTA 20mg dispersible tablets |
| GP | EVENT_CD = 'd4rP.' -- ZALASTA 10mg tablets |
| GP | EVENT_CD = 'd4rt.' -- OLANZAPINE 20mg tablets |
| GP | EVENT_CD = 'd4ru.' -- OLANZAPINE 20mg dispersible tablets |
| GP | EVENT_CD = 'd4rv.' -- OLANZAPINE 10mg injection (pdr frecon) |
| GP | EVENT_CD = 'd4rw.' -- OLANZAPINE 15mg dispersible tablets |
| GP | EVENT_CD = 'd4rx.' -- OLANZAPINE 15mg tablets |
| GP | EVENT_CD = 'd4ry.' -- OLANZAPINE 5mg dispersible tablets |
| GP | EVENT_CD = 'd4rz.' -- OLANZAPINE 10mg dispersible tablets |
| GP | EVENT_CD = 'd4s..' -- QUETIAPINE |
| GP | EVENT_CD = 'd4s1.' -- QUETIAPINE 25mg tablets |
| GP | EVENT_CD = 'd4s2.' -- QUETIAPINE 100mg tablets |
| GP | EVENT_CD = 'd4s3.' -- QUETIAPINE 200mg tablets |
| GP | EVENT_CD = 'd4s4.' -- QUETIAPINE 25mg+100mg tablets starter pack |
| GP | EVENT_CD = 'd4s5.' -- SEROQUEL 25mg tablets |
| GP | EVENT_CD = 'd4s6.' -- SEROQUEL 100mg tablets |
| GP | EVENT_CD = 'd4s7.' -- SEROQUEL 200mg tablets |
| GP | EVENT_CD = 'd4s8.' -- SEROQUEL 25mg+100mg tablets starter pack |
| GP | EVENT_CD = 'd4s9.' -- SEROQUEL 150mg tablets |
| GP | EVENT_CD = 'd4sA.' -- SEROQUEL 25mg+100mg+150mg tablets starter pack |
| GP | EVENT_CD = 'd4sB.' -- SEROQUEL 300mg tablets |
| GP | EVENT_CD = 'd4sC.' -- SEROQUEL XL 50mg m/r tablets |
| GP | EVENT_CD = 'd4sD.' -- SEROQUEL XL 200mg m/r tablets |
| GP | EVENT_CD = 'd4sE.' -- SEROQUEL XL 300mg m/r tablets |
| GP | EVENT_CD = 'd4sF.' -- SEROQUEL XL 400mg m/r tablets |
| GP | EVENT_CD = 'd4sG.' -- SEROQUEL XL 150mg m/r tablets |
| GP | EVENT_CD = 'd4ss.' -- QUETIAPINE 150mg m/r tablets |
| GP | EVENT_CD = 'd4st.' -- QUETIAPINE 400mg m/r tablets |
| GP | EVENT_CD = 'd4su.' -- QUETIAPINE 300mg m/r tablets |
| GP | EVENT_CD = 'd4sv.' -- QUETIAPINE 200mg m/r tablets |
| GP | EVENT_CD = 'd4sw.' -- QUETIAPINE 50mg m/r tablets |
| GP | EVENT_CD = 'd4sx.' -- QUETIAPINE 300mg tablets |
| GP | EVENT_CD = 'd4sy.' -- QUETIAPINE 25mg+100mg+150mg tablets starter pack |
| GP | EVENT_CD = 'd4sz.' -- QUETIAPINE 150mg tablets |
| GP | EVENT_CD = 'd4t..' -- AMISULPRIDE |
| GP | EVENT_CD = 'd4t1.' -- AMISULPRIDE 50mg tablets |
| GP | EVENT_CD = 'd4t2.' -- AMISULPRIDE 200mg tablets |
| GP | EVENT_CD = 'd4t3.' -- SOLIAN 50mg tablets |
| GP | EVENT_CD = 'd4t4.' -- SOLIAN 200mg tablets |
| GP | EVENT_CD = 'd4t5.' -- SOLIAN 400mg tablets |
| GP | EVENT_CD = 'd4t6.' -- SOLIAN 100mg/mL sugar free oral solution |
| GP | EVENT_CD = 'd4t7.' -- SOLIAN 100mg tablets |
| GP | EVENT_CD = 'd4tx.' -- AMISULPRIDE 100mg tablets |
| GP | EVENT_CD = 'd4ty.' -- AMISULPRIDE 100mg/mL sugar free oral solution |
| GP | EVENT_CD = 'd4tz.' -- AMISULPRIDE 400mg tablets |
| GP | EVENT_CD = 'd4u..' -- ZOTEPINE |
| GP | EVENT_CD = 'd4u1.' -- *ZOTEPINE 25mg tablets |
| GP | EVENT_CD = 'd4u2.' -- *ZOTEPINE 50mg tablets |
| GP | EVENT_CD = 'd4u3.' -- *ZOTEPINE 100mg tablets |
| GP | EVENT_CD = 'd4u4.' -- *ZOLEPTIL 25mg tablets |
| GP | EVENT_CD = 'd4u5.' -- *ZOLEPTIL 50mg tablets |
| GP | EVENT_CD = 'd4u6.' -- *ZOLEPTIL 100mg tablets |
| GP | EVENT_CD = 'd4v..' -- ARIPIPRAZOLE |
| GP | EVENT_CD = 'd4v1.' -- ABILIFY 10mg tablets |
| GP | EVENT_CD = 'd4v2.' -- ABILIFY 15mg tablets |
| GP | EVENT_CD = 'd4v3.' -- ABILIFY 30mg tablets |
| GP | EVENT_CD = 'd4v4.' -- ABILIFY 5mg tablets |
| GP | EVENT_CD = 'd4v5.' -- ABILIFY 10mg oro-dispersible tablets |
| GP | EVENT_CD = 'd4v6.' -- ABILIFY 15mg oro-dispersible tablets |
| GP | EVENT_CD = 'd4v7.' -- ABILIFY 1mg/mL oral solution |
| GP | EVENT_CD = 'd4v8.' -- ABILIFY 9.75mg/1.3mL solution finjection |
| GP | EVENT_CD = 'd4vs.' -- ARIPIPRAZOLE 9.75mg/1.3mL solution finjection |
| GP | EVENT_CD = 'd4vt.' -- ARIPIPRAZOLE 1mg/mL oral solution |
| GP | EVENT_CD = 'd4vu.' -- ARIPIPRAZOLE 10mg oro-dispersible tablets |
| GP | EVENT_CD = 'd4vv.' -- ARIPIPRAZOLE 15mg oro-dispersible tablets |
| GP | EVENT_CD = 'd4vw.' -- ARIPIPRAZOLE 5mg tablets |
| GP | EVENT_CD = 'd4vx.' -- ARIPIPRAZOLE 30mg tablets |
| GP | EVENT_CD = 'd4vy.' -- ARIPIPRAZOLE 15mg tablets |
| GP | EVENT_CD = 'd4vz.' -- ARIPIPRAZOLE 10mg tablets |
| GP | EVENT_CD = 'd4w..' -- PALIPERIDONE |
| GP | EVENT_CD = 'd4w1.' -- INVEGA 3mg m/r tablets |
| GP | EVENT_CD = 'd4w2.' -- INVEGA 6mg m/r tablets |
| GP | EVENT_CD = 'd4w3.' -- INVEGA 9mg m/r tablets |
| GP | EVENT_CD = 'd4w4.' -- *INVEGA 12mg m/r tablets |
| GP | EVENT_CD = 'd4w5.' -- XEPLION 50mg suspension finjection prefilled syringe |
| GP | EVENT_CD = 'd4w6.' -- XEPLION 75mg suspension finjection prefilled syringe |
| GP | EVENT_CD = 'd4w7.' -- XEPLION 100mg suspension finjection prefilled syringe |
| GP | EVENT_CD = 'd4w8.' -- XEPLION 150mg suspension finjection prefilled syringe |
| GP | EVENT_CD = 'd4ws.' -- PALIPERIDONE 150mg suspension finjection pfs |
| GP | EVENT_CD = 'd4wt.' -- PALIPERIDONE 100mg suspension finjection pfs |
| GP | EVENT_CD = 'd4wu.' -- PALIPERIDONE 75mg suspension finjection prefilled syringe |
| GP | EVENT_CD = 'd4wv.' -- PALIPERIDONE 50mg suspension finjection prefilled syringe |
| GP | EVENT_CD = 'd4ww.' -- *PALIPERIDONE 12mg m/r tablets |
| GP | EVENT_CD = 'd4wx.' -- PALIPERIDONE 9mg m/r tablets |
| GP | EVENT_CD = 'd4wy.' -- PALIPERIDONE 6mg m/r tablets |
| GP | EVENT_CD = 'd4wz.' -- PALIPERIDONE 3mg m/r tablets |
| GP | EVENT_CD = 'd4x..' -- ASENAPINE |
| GP | EVENT_CD = 'd4x1.' -- SYCREST 5mg sublingual tablets |
| GP | EVENT_CD = 'd4x2.' -- ASENAPINE 5mg sublingual tablets |
| GP | EVENT_CD = 'd4x3.' -- SYCREST 10mg sublingual tablets |
| GP | EVENT_CD = 'd4x4.' -- ASENAPINE 10mg sublingual tablets |
| GP | EVENT_CD = 'd58..' -- OLANZAPINE PAMOATE |
| GP | EVENT_CD = 'd581.' -- ZYPADHERA 210mg powder+solvent fsuspension finjection |
| GP | EVENT_CD = 'd582.' -- ZYPADHERA 300mg powder+solvent fsuspension finjection |
| GP | EVENT_CD = 'd583.' -- ZYPADHERA 405mg powder+solvent fsuspension finjection |
| GP | EVENT_CD = 'd58x.' -- OLANZAPINE 405mg powder+solvent fsuspension finjection |
| GP | EVENT_CD = 'd58y.' -- OLANZAPINE 300mg powder+solvent fsuspension finjection |
| GP | EVENT_CD = 'd58z.' -- OLANZAPINE 210mg powder+solvent fsuspension finjection |

Other diagnosis:

Psychiatric diagnosis

| GP | '1464.', -- H/O: schizophrenia |
| --- | --- |
| GP | '212T.', -- Psychosis, schizophrenia + bipolar affective disord resolved |
| GP | '212W.', -- Schizophrenia resolved |
| GP | 'E100.', -- Simple schizophrenia |
| GP | 'E1000', -- Unspecified schizophrenia |
| GP | 'E1001', -- Subchronic schizophrenia |
| GP | 'E1003', -- Acute exacerbation of subchronic schizophrenia |
| GP | 'E1004', -- Acute exacerbation of chronic schizophrenia |
| GP | 'E1005', -- Schizophrenia in remission |
| GP | 'E100z', -- Simple schizophrenia NOS |
| GP | 'E101.', -- Hebephrenic schizophrenia |
| GP | 'E1010', -- Unspecified hebephrenic schizophrenia |
| GP | 'E1011', -- Subchronic hebephrenic schizophrenia |
| GP | 'E1012', -- Chronic hebephrenic schizophrenia |
| GP | 'E1013', -- Acute exacerbation of subchronic hebephrenic schizophrenia |
| GP | 'E1014', -- Acute exacerbation of chronic hebephrenic schizophrenia |
| GP | 'E1015', -- Hebephrenic schizophrenia in remission |
| GP | 'E101z', -- Hebephrenic schizophrenia NOS |
| GP | 'E102.', -- Catatonic schizophrenia |
| GP | 'E1020', -- Unspecified catatonic schizophrenia |
| GP | 'E1021', -- Subchronic catatonic schizophrenia |
| GP | 'E1022', -- Chronic catatonic schizophrenia |
| GP | 'E1023', -- Acute exacerbation of subchronic catatonic schizophrenia |
| GP | 'E1024', -- Acute exacerbation of chronic catatonic schizophrenia |
| GP | 'E1025', -- Catatonic schizophrenia in remission |
| GP | 'E102z', -- Catatonic schizophrenia NOS |
| GP | 'E103.', -- Paranoid schizophrenia |
| GP | 'E1030', -- Unspecified paranoid schizophrenia |
| GP | 'E1031', -- Subchronic paranoid schizophrenia |
| GP | 'E1032', -- Chronic paranoid schizophrenia |
| GP | 'E1033', -- Acute exacerbation of subchronic paranoid schizophrenia |
| GP | 'E1034', -- Acute exacerbation of chronic paranoid schizophrenia |
| GP | 'E1035', -- Paranoid schizophrenia in remission |
| GP | 'E103z', -- Paranoid schizophrenia NOS |
| GP | 'E105.', -- Latent schizophrenia |
| GP | 'E1050', -- Unspecified latent schizophrenia |
| GP | 'E1051', -- Subchronic latent schizophrenia |
| GP | 'E1052', -- Chronic latent schizophrenia |
| GP | 'E1053', -- Acute exacerbation of subchronic latent schizophrenia |
| GP | 'E1054', -- Acute exacerbation of chronic latent schizophrenia |
| GP | 'E1055', -- Latent schizophrenia in remission |
| GP | 'E105z', -- Latent schizophrenia NOS |
| GP | 'E106.', -- Residual schizophrenia |
| GP | 'E107.', -- Schizo-affective schizophrenia |
| GP | 'E1070', -- Unspecified schizo-affective schizophrenia |
| GP | 'E1071', -- Subchronic schizo-affective schizophrenia |
| GP | 'E1072', -- Chronic schizo-affective schizophrenia |
| GP | 'E1073', -- Acute exacerbation subchronic schizo-affective schizophrenia |
| GP | 'E1074', -- Acute exacerbation of chronic schizo-affective schizophrenia |
| GP | 'E1075', -- Schizo-affective schizophrenia in remission |
| GP | 'E107z', -- Schizo-affective schizophrenia NOS |
| GP | 'E10y.', -- Other schizophrenia |
| GP | 'E10y0', -- Atypical schizophrenia |
| GP | 'E10y1', -- Coenesthopathic schizophrenia |
| GP | 'E10yz', -- Other schizophrenia NOS |
| GP | 'E10z.', -- Schizophrenia NOS |
| GP | 'Eu052', -- [X]Organic delusional [schizophrenia-like] disorder |
| GP | 'Eu2..', -- [X]Schizophrenia, schizotypal and delusional disorders |
| GP | 'Eu20.', -- [X]Schizophrenia |
| GP | 'Eu200', -- [X]Paranoid schizophrenia |
| GP | 'Eu201', -- [X]Hebephrenic schizophrenia |
| GP | 'Eu202', -- [X]Catatonic schizophrenia |
| GP | 'Eu203', -- [X]Undifferentiated schizophrenia |
| GP | 'Eu205', -- [X]Residual schizophrenia |
| GP | 'Eu206', -- [X]Simple schizophrenia |
| GP | 'Eu20y', -- [X]Other schizophrenia |
| GP | 'Eu20z', -- [X]Schizophrenia, unspecified |
| GP | 'Eu232', -- [X]Acute schizophrenia-like psychotic disorder |
| GP | 'ZV110', -- [V]Personal history of schizophrenia |
| GP | '212T.', -- Psychosis, schizophrenia + bipolar affective disord resolved |
| GP | '212V.', -- Bipolar affective disorder resolved |
| GP | 'E114.', -- Bipolar affective disorder, currently manic |
| GP | 'E1140', -- Bipolar affective disorder, currently manic, unspecified |
| GP | 'E1141', -- Bipolar affective disorder, currently manic, mild |
| GP | 'E1142', -- Bipolar affective disorder, currently manic, moderate |
| GP | 'E1143', -- Bipolar affect disord, currently manic, severe, no psychosis |
| GP | 'E1144', -- Bipolar affect disord, currently manic,severe with psychosis |
| GP | 'E1145', -- Bipolar affect disord,currently manic, part/unspec remission |
| GP | 'E1146', -- Bipolar affective disorder, currently manic, full remission |
| GP | 'E114z', -- Bipolar affective disorder, currently manic, NOS |
| GP | 'E115.', -- Bipolar affective disorder, currently depressed |
| GP | 'E1150', -- Bipolar affective disorder, currently depressed, unspecified |
| GP | 'E1151', -- Bipolar affective disorder, currently depressed, mild |
| GP | 'E1152', -- Bipolar affective disorder, currently depressed, moderate |
| GP | 'E1153', -- Bipolar affect disord, now depressed, severe, no psychosis |
| GP | 'E1154', -- Bipolar affect disord, now depressed, severe with psychosis |
| GP | 'E1155', -- Bipolar affect disord, now depressed, part/unspec remission |
| GP | 'E1156', -- Bipolar affective disorder, now depressed, in full remission |
| GP | 'E115z', -- Bipolar affective disorder, currently depressed, NOS |
| GP | 'E116.', -- Mixed bipolar affective disorder |
| GP | 'E1160', -- Mixed bipolar affective disorder, unspecified |
| GP | 'E1161', -- Mixed bipolar affective disorder, mild |
| GP | 'E1162', -- Mixed bipolar affective disorder, moderate |
| GP | 'E1163', -- Mixed bipolar affective disorder, severe, without psychosis |
| GP | 'E1164', -- Mixed bipolar affective disorder, severe, with psychosis |
| GP | 'E1165', -- Mixed bipolar affective disorder, partial/unspec remission |
| GP | 'E1166', -- Mixed bipolar affective disorder, in full remission |
| GP | 'E116z', -- Mixed bipolar affective disorder, NOS |
| GP | 'E117.', -- Unspecified bipolar affective disorder |
| GP | 'E1170', -- Unspecified bipolar affective disorder, unspecified |
| GP | 'E1171', -- Unspecified bipolar affective disorder, mild |
| GP | 'E1172', -- Unspecified bipolar affective disorder, moderate |
| GP | 'E1173', -- Unspecified bipolar affective disorder, severe, no psychosis |
| GP | 'E1174', -- Unspecified bipolar affective disorder,severe with psychosis |
| GP | 'E1175', -- Unspecified bipolar affect disord, partial/unspec remission |
| GP | 'E1176', -- Unspecified bipolar affective disorder, in full remission |
| GP | 'E117z', -- Unspecified bipolar affective disorder, NOS |
| GP | 'Eu31.', -- [X]Bipolar affective disorder |
| GP | 'Eu310', -- [X]Bipolar affective disorder, current episode hypomanic |
| GP | 'Eu311', -- [X]Bipolar affect disorder cur epi manic wout psychotic symp |
| GP | 'Eu312', -- [X]Bipolar affect disorder cur epi manic with psychotic symp |
| GP | Eu313', -- [X]Bipolar affect disorder cur epi mild moderate depressn |
| GP | 'Eu315', -- [X]Bipolar affect dis cur epi severe depres with psyc symp |
| GP | 'Eu316', -- [X]Bipolar affective disorder, current episode mixed |
| GP | 'Eu317', -- [X]Bipolar affective disorder, currently in remission |
| GP | 'Eu318', -- [X]Bipolar affective disorder type I |
| GP | 'Eu319', -- [X]Bipolar affective disorder type II |
| GP | 'Eu31y', -- [X]Other bipolar affective disorders |
| GP | 'Eu31z', -- [X]Bipolar affective disorder, unspecified |
| GP | '128A.', -- FH: Psychotic illness |
| GP | 'E0...', -- Organic psychotic conditions |
| GP | 'E00..', -- Senile and presenile organic psychotic conditions |
| GP | 'Eu105', -- [X]Mental & behav dis due to use alcohol: psychotic disorder |
| GP | 'Eu115', -- [X]Mental & behav dis due to use opioids: psychotic disorder |
| GP | 'Eu125', -- [X]Mental & behav dis due to cannabinoids: psychotic disordr |
| GP | 'Eu135', -- [X]Mental & behav dis due to seds/hypntcs: psychotic disordr |
| GP | 'Eu145', -- [X]Mental & behav dis due to use cocaine: psychotic disorder |
| GP | 'Eu155', -- [X]Mental/behav dis oth stims inc caffeine: psychotic dis |
| GP | 'Eu165', -- [X]Mental & behav dis due to hallucinogens: psychotic disord |
| GP | 'Eu175', -- [X]Mental & behav dis due to use tobacco: psychotic disorder |
| GP | 'Eu185', -- [X]Mental & behav dis due to vol solvents: psychotic disordr |
| GP | 'Eu187', -- [X]Mnt/bh dis vol solvents: resid & late-onset psychotic dis |
| GP | 'Eu1A5', -- [X]Mental behav disord due crack cocaine: psychotic disorder |
| GP | 'Eu23.', -- [X]Acute and transient psychotic disorders |
| GP | 'Eu232', -- [X]Acute schizophrenia-like psychotic disorder |
| GP | 'Eu233', -- [X]Other acute predominantly delusional psychotic disorders |
| GP | 'Eu23y', -- [X]Other acute and transient psychotic disorders |
| GP | 'Eu23z', -- [X]Acute and transient psychotic disorder, unspecified |
| GP | 'Eu2y.', -- [X]Other nonorganic psychotic disorders |
| GP | 'Eu301', -- [X]Mania without psychotic symptoms |
| GP | 'Eu302', -- [X]Mania with psychotic symptoms |
| GP | 'Eu312', -- [X]Bipolar affect disorder cur epi manic with psychotic symp |
| GP | 'Eu323', -- [X]Severe depressive episode with psychotic symptoms |
| GP | Eu328', -- [X]Majdepression, severe with psychotic symptoms |
| GP | '1287.', -- FH: Manic depressive state |
| GP | '146D.', -- H/O: manic depressive disorder |
| GP | E112.', -- Single majdepressive episode |
| GP | E1120', -- Single majdepressive episode, unspecified |
| GP | E1121', -- Single majdepressive episode, mild |
| GP | E1122', -- Single majdepressive episode, moderate |
| GP | E1124', -- Single majdepressive episode, severe, with psychosis |
| GP | E1125', -- Single majdepressive episode, partial unspec remission |
| GP | E1126', -- Single majdepressive episode, in full remission |
| GP | E112z', -- Single majdepressive episode NOS |
| GP | E113.', -- Recurrent majdepressive episode |
| GP | E1130', -- Recurrent majdepressive episodes, unspecified |
| GP | E1131', -- Recurrent majdepressive episodes, mild |
| GP | E1132', -- Recurrent majdepressive episodes, moderate |
| GP | E1133', -- Recurrent majdepressive episodes, severe, no psychosis |
| GP | E1134', -- Recurrent majdepressive episodes, severe, with psychosis |
| GP | E1135', -- Recurrent majdepressive episodes,partial/unspec remission |
| GP | E1136', -- Recurrent majdepressive episodes, in full remission |
| GP | E113z', -- Recurrent majdepressive episode NOS |
| GP | 'E11y.', -- Other and unspecified manic-depressive psychoses |
| GP | 'E11y0', -- Unspecified manic-depressive psychoses |
| GP | 'E11y2', -- Atypical depressive disorder |
| GP | 'E11y3', -- Other mixed manic-depressive psychoses |
| GP | 'E11yz', -- Other and unspecified manic-depressive psychoses NOS |
| GP | 'Eu412', -- [X]Mixed anxiety and depressive disorder |
| HOSPITAL ADMISSION | 'F06%' |
| HOSPITAL ADMISSION | DIAG_CD_1234 LIKE 'F2%' |
| HOSPITAL ADMISSION | DIAG_CD_1234 LIKE 'F31%' |
| HOSPITAL ADMISSION | DIAG_CD_1234 LIKE 'F30%' |
| HOSPITAL ADMISSION | DIAG_CD_1234 = 'F333' |
| HOSPITAL ADMISSION | DIAG_CD_1234 = 'F322' |
| HOSPITAL ADMISSION | DIAG_CD_1234 = 'F323' |

Tourettes

| GP | 'E2723', -- Gilles de la Tourette's disorder |
| --- | --- |
| GP | 'Eu952' -- [X]Comb vocal multiple motor tic disorder - de la Tourette |
| HOSPITAL ADMISSION | 'F95%' -- TOURETTES |

Aggression

| GP | '1P5..', -- Aggressive behaviour |
| --- | --- |
| GP | 'E2930', -- Adjustment reaction with aggression |
| GP | 'E2C0.', -- Aggressive unsocial conduct disorder |
| GP | 'E2C00', -- Aggressive outburst |
| GP | 'E2C0z' -- Aggressive unsocial conduct disorder NOS |
| HOSPITAL ADMISSION | 'F91%' -- AGGRESION |
| HOSPITAL ADMISSION | DIAG_CD_1234 = 'F603' -- AGGRESION |

Downs Syndrome

| **DATABASE** | **CODES** |
| --- | --- |
| GP | EVENT_CD = 'PJ0..' -- Down's syndrome - trisomy 21 |
| GP | EVENT_CD = 'PJ00.' -- Trisomy 21, meiotic nondisjunction |
| GP | EVENT_CD = 'PJ01.' -- Trisomy 21, mosaicism |
| GP | EVENT_CD = 'PJ02.' -- Trisomy 21, translocation |
| GP | EVENT_CD = 'PJ0z.' -- Down's syndrome NOS |
| HOSPITAL ADMISSION | DIAG_CD_1234 = 'Q900' -- Trisomy 21, meiotic nondisjunction |
| HOSPITAL ADMISSION | DIAG_CD_1234 = 'Q901' -- Trisomy 21, mosaicism (mitotic nondisjunction) |
| HOSPITAL ADMISSION | DIAG_CD_1234 = 'Q902' -- Trisomy 21, translocation |
| HOSPITAL ADMISSION | DIAG_CD_1234 = 'Q909' -- Down's syndrome, unspecified |
| HOSPITAL ADMISSION | (SYNDROME_ICD LIKE 'Q90%') |
| HOSPITAL ADMISSION | (SYNDROME_ICD_2 LIKE 'Q90%') |
| HOSPITAL ADMISSION | ANOMALY_CD = 'Q18.9' -- Trisomy 21, meiotic nondisjunction |

Epilepsy

|  | **CODE** | **DESCRIPTION** |
| --- | --- | --- |
| GP | F25.. | Epilepsy |
| GP | F250. | Generalised nonconvulsive epilepsy |
| GP | F2500 | Petit mal (minor) epilepsy |
| GP | F2501 | Pykno-epilepsy |
| GP | F2502 | Epileptic seizures - atonic |
| GP | F2503 | Epileptic seizures - akinetic |
| GP | F2504 | Juvenile absence epilepsy |
| GP | F2505 | Lennox-Gastaut syndrome |
| GP | F250y | Other specified generalised nonconvulsive epilepsy |
| GP | F250z | Generalised nonconvulsive epilepsy NOS |
| GP | F251. | Generalised convulsive epilepsy |
| GP | F2510 | Grand mal (major) epilepsy |
| GP | F2511 | Neonatal myoclonic epilepsy |
| GP | F2512 | Epileptic seizures - clonic |
| GP | F2513 | Epileptic seizures - myoclonic |
| GP | F2514 | Epileptic seizures - tonic |
| GP | F2515 | Tonic-clonic epilepsy |
| GP | F2516 | Grand mal seizure |
| GP | F251y | Other specified generalised convulsive epilepsy |
| GP | F251z | Generalised convulsive epilepsy NOS |
| GP | F252. | Petit mal status |
| GP | F253. | Grand mal status |
| GP | F254. | Partial epilepsy with impairment of consciousness |
| GP | F2540 | Temporal lobe epilepsy |
| GP | F2541 | Psychomotor epilepsy |
| GP | F2542 | Psychosensory epilepsy |
| GP | F2543 | Limbic system epilepsy |
| GP | F2544 | Epileptic automatism |
| GP | F2545 | Complex partial epileptic seizure |
| GP | F254z | Partial epilepsy with impairment of consciousness NOS |
| GP | F255. | Partial epilepsy without impairment of consciousness |
| GP | F2550 | Jacksonian, focal or motor epilepsy |
| GP | F2551 | Sensory induced epilepsy |
| GP | F2552 | Somatosensory epilepsy |
| GP | F2553 | Visceral reflex epilepsy |
| GP | F2554 | Visual reflex epilepsy |
| GP | F2555 | Unilateral epilepsy |
| GP | F2556 | Simple partial epileptic seizure |
| GP | F255y | Partial epilepsy without impairment of consciousness OS |
| GP | F255z | Partial epilepsy without impairment of consciousness NOS |
| GP | F256. | Infantile spasms |
| GP | F256. | Infantile spasms |
| GP | F2560 | Hypsarrhythmia |
| GP | F2561 | Salaam attacks |
| GP | F256z | Infantile spasms NOS |
| GP | F257. | Kojevnikov's epilepsy |
| GP | F258. | Post-ictal state |
| GP | F259. | Early infant epileptic encephalopathy wth suppression bursts |
| GP | F25A. | Juvenile myoclonic epilepsy |
| GP | F25B. | Alcohol-induced epilepsy |
| GP | F25C. | Drug-induced epilepsy |
| GP | F25D. | Menstrual epilepsy |
| GP | F25E. | Stress-induced epilepsy |
| GP | F25F. | Photosensitive epilepsy |
| GP | F25X. | Status epilepticus, unspecified |
| GP | F25y. | Other forms of epilepsy |
| GP | F25y0 | Cursive (running) epilepsy |
| GP | F25y1 | Gelastic epilepsy |
| GP | F25y2 | Locl-rlt(foc)(part)idiop epilep&epilptic syn seiz locl onset |
| GP | F25y3 | Complex partial status epilepticus |
| GP | F25y4 | Benign Rolandic epilepsy |
| GP | F25y5 | Panayiotopoulos syndrome |
| GP | F25yz | Other forms of epilepsy NOS |
| GP | F25z. | Epilepsy NOS |
| GP | 667.. | Epilepsy monitoring |
| GP | 6671 | Initial epilepsy assessment |
| GP | 6672 | Follow-up epilepsy assessment |
| GP | 6673 | Driving licence |
| GP | 6674 | Epilepsy associated problems |
| GP | 6675 | Fit frequency |
| GP | 6676 | Last fit |
| GP | 6677 | Epilepsy drug side effects |
| GP | 6678 | Epilepsy treatment changed |
| GP | 6679 | Epilepsy treatment started |
| GP | 667A. | Epilepsy treatment stopped |
| GP | 667B. | Nocturnal epilepsy |
| GP | 667C. | Epilepsy control good |
| GP | 667D. | Epilepsy control poor |
| GP | 667E. | Epilepsy care arrangement |
| GP | 667F. | Seizure free >12 months |
| GP | 667G. | Epilepsy restricts employment |
| GP | 667H. | Epilepsy prevents employment |
| GP | 667J. | Epilepsy impairs education |
| GP | 667K. | Epilepsy limits activities |
| GP | 667L. | Epilepsy does not limit activities |
| GP | 667M. | Epilepsy management plan given |
| GP | 667N. | Epilepsy severity |
| GP | 667P. | No seizures on treatment |
| GP | 667Q. | 1 to 12 seizures a year |
| GP | 667R. | 2 to 4 seizures a month |
| GP | 667S. | 1 to 7 seizures a week |
| GP | 667T. | Daily seizures |
| GP | 667V. | Many seizures a day |
| GP | 667W. | Emergency epilepsy treatment since last appointment |
| GP | 667X. | No epilepsy drug side effects |
| GP | 667Z. | Epilepsy monitoring NOS |
| GP | 1030. |  |
| GP | dn... | CONTROL OF EPILEPSY |
| GP | dn1.. | ACETAZOLAMIDE [EPILEPSY] |
| GP | dn11. | DIAMOX [EP] 500mg m/r capsules |
| GP | dn12. | DIAMOX [EP] 250mg tablets |
| GP | dn13. | DIAMOX [EP] 500mg injection |
| GP | dn1x. | ACETAZOLAMIDE [EP] 500mg m/r capsules |
| GP | dn1y. | ACETAZOLAMIDE [EP] 250mg tablets |
| GP | dn1z. | ACETAZOLAMIDE [EP] 500mg injection |
| GP | dn2.. | *BECLAMIDE |
| GP | dn21. | *NYDRANE 500mg tablets |
| GP | dn2z. | *BECLAMIDE 500mg tablets |
| GP | dn3.. | CARBAMAZEPINE |
| GP | dn31. | CARBAMAZEPINE 100mg tablets |
| GP | dn32. | CARBAMAZEPINE 200mg tablets |
| GP | dn33. | CARBAMAZEPINE 400mg tablets |
| GP | dn34. | TEGRETOL 100mg tablets |
| GP | dn35. | TEGRETOL 200mg tablets |
| GP | dn36. | TEGRETOL 400mg tablets |
| GP | dn37. | TEGRETOL 100mg/5mL sugar free liquid |
| GP | dn38. | TEGRETOL RETARD 200mg m/r tabs |
| GP | dn39. | TEGRETOL RETARD 400mg m/r tabs |
| GP | dn3a. | CARBAMAZEPINE 200mg m/r tabs |
| GP | dn3A. | *EPIMAZ 100mg tablets |
| GP | dn3b. | CARBAMAZEPINE 400mg m/r tabs |
| GP | dn3B. | *EPIMAZ 200mg tablets |
| GP | dn3c. | TEGRETOL 100mg chewable tablets |
| GP | dn3C. | *EPIMAZ 400mg tablets |
| GP | dn3d. | TEGRETOL 200mg chewable tablets |
| GP | dn3D. | TEGRETOL 125mg suppositories |
| GP | dn3e. | *ARBIL MR 200mg m/r tablets |
| GP | dn3E. | TEGRETOL 250mg suppositories |
| GP | dn3f. | *ARBIL MR 400mg m/r tablets |
| GP | dn3F. | TIMONIL RETARD 200mg m/r tablets |
| GP | dn3G. | TIMONIL RETARD 400mg m/r tablets |
| GP | dn3H. | *TERIL CR 200mg m/r tablets |
| GP | dn3I. | *TERIL CR 400mg m/r tablets |
| GP | dn3J. | CARBAGEN SR 200mg m/r tablets |
| GP | dn3K. | CARBAGEN SR 400mg m/r tablets |
| GP | dn3v. | CARBAMAZEPINE 125mg suppositories |
| GP | dn3w. | CARBAMAZEPINE 250mg suppositories |
| GP | dn3x. | CARBAMAZEPINE 200mg chewable tablets |
| GP | dn3y. | CARBAMAZEPINE 100mg chewable tablets |
| GP | dn3z. | CARBAMAZEPINE 100mg/5mL sugar free liquid |
| GP | dn4.. | CLONAZEPAM [EPILEPSY CONTROL] |
| GP | dn41. | RIVOTRIL 500micrograms tablets |
| GP | dn42. | RIVOTRIL 2mg tablets |
| GP | dn4w. | CLONAZEPAM 0.5mg/5mL sugar free oral solution |
| GP | dn4x. | CLONAZEPAM 2mg/5mL sugar free oral solution |
| GP | dn4y. | CLONAZEPAM 500microgram tablets |
| GP | dn4z. | CLONAZEPAM 2mg tablets |
| GP | dn5.. | ETHOSUXIMIDE |
| GP | dn51. | *ETHOSUXIMIDE 250mg capsules |
| GP | dn52. | *ETHOSUXIMIDE 250mg/5mL elixir |
| GP | dn53. | *EMESIDE 250mg capsules |
| GP | dn54. | EMESIDE 250mg/5mL syrup |
| GP | dn55. | *ZARONTIN 250mg capsules |
| GP | dn56. | ZARONTIN 250mg/5mL syrup |
| GP | dn5x. | ETHOSUXIMIDE 250mg capsules |
| GP | dn5y. | *ETHOSUXIMIDE 250mg/5mL elixir |
| GP | dn5z. | ETHOSUXIMIDE 250mg/5mL syrup |
| GP | dn6.. | METHYLPHENOBARBITAL |
| GP | dn61. | *PROMINAL 30mg tablets |
| GP | dn62. | *PROMINAL 60mg tablets |
| GP | dn63. | *PROMINAL 200mg tablets |
| GP | dn6x. | METHYLPHENOBARBITONE 30mg tablets |
| GP | dn6y. | METHYLPHENOBARBITONE 60mg tablets |
| GP | dn6z. | METHYLPHENOBARBITONE 200mg tablets |
| GP | dn7.. | PHENOBARBITAL |
| GP | dn71. | PHENOBARBITAL 15mg tablets |
| GP | dn72. | PHENOBARBITAL 30mg tablets |
| GP | dn73. | PHENOBARBITAL 60mg tablets |
| GP | dn74. | PHENOBARBITAL 100mg tablets |
| GP | dn75. | PHENOBARBITONE SODIUM 30mg tablets |
| GP | dn76. | PHENOBARBITONE SODIUM 60mg tablets |
| GP | dn77. | PHENOBARBITONE 15mg/10mL elixir |
| GP | dn78. | PHENOBARBITAL 200mg/1mL injection |
| GP | dn79. | *GARDENAL 200mg/1mL injection |
| GP | dn7a. | *LUMINAL 15mg tablets |
| GP | dn7b. | *LUMINAL 30mg tablets |
| GP | dn7c. | *LUMINAL 60mg tablets |
| GP | dn7d. | PHENOBARBITAL 15mg/5mL elixir |
| GP | dn8.. | PHENYTOIN |
| GP | dn81. | EPANUTIN 30mg/5mL suspension |
| GP | dn82. | EPANUTIN 50mg Infatabs |
| GP | dn83. | PHENYTOIN 50mg chewable tablets |
| GP | dn8y. | PHENYTOIN 30mg/5mL suspension |
| GP | dn8z. | PHENYTOIN 90mg/5mL sugar free suspension |
| GP | dn9.. | PHENYTOIN SODIUM |
| GP | dn91. | PHENYTOIN 50mg tablets |
| GP | dn92. | PHENYTOIN 100mg tablets |
| GP | dn93. | EPANUTIN 25mg capsules |
| GP | dn94. | EPANUTIN 50mg capsules |
| GP | dn95. | EPANUTIN 100mg capsules |
| GP | dn96. | EPANUTIN 300mg capsules |
| GP | dn97. | *PENTRAN 50mg tablets |
| GP | dn98. | *PENTRAN 100mg tablets |
| GP | dn9w. | PHENYTOIN SODIUM 300mg capsules |
| GP | dn9x. | PHENYTOIN SODIUM 25mg caps |
| GP | dn9y. | PHENYTOIN SODIUM 50mg capsules |
| GP | dn9z. | PHENYTOIN SODIUM 100mg capsules |
| GP | dna.. | PRIMIDONE |
| GP | dna1. | MYSOLINE 250mg tablets |
| GP | dna2. | MYSOLINE 250mg/5mL oral suspension |
| GP | dna3. | MYSOLINE 50mg tablets |
| GP | dnax. | PRIMIDONE 50mg tablets |
| GP | dnay. | PRIMIDONE 250mg tablets |
| GP | dnaz. | PRIMIDONE 250mg/5mL oral suspension |
| GP | dnb.. | SODIUM VALPROATE |
| GP | dnb1. | EPILIM 100mg crushable tablets |
| GP | dnb2. | EPILIM 200mg e/c tablets |
| GP | dnb3. | EPILIM 500mg e/c tablets |
| GP | dnb4. | EPILIM 200mg/5mL sugar free liquid |
| GP | dnb5. | EPILIM 200mg/5mL syrup |
| GP | dnb6. | EPILIM IV 400mg/4mL injection |
| GP | dnb7. | SODIUM VALPROATE 200mg e/c tablets |
| GP | dnb8. | SODIUM VALPROATE 500mg e/c tablets |
| GP | dnb9. | ORLEPT STARTER PACK 200mg e/c tablets x10 |
| GP | dnba. | *ORLEPT 200mg e/c tablets |
| GP | dnbA. | ORLEPT 200mg/5mL sugar free liquid |
| GP | dnbb. | *ORLEPT 500mg e/c tablets |
| GP | dnbB. | EPIVAL CR 300mg m/r tablets |
| GP | dnbc. | EPILIM CHRONO 200 m/r tablets |
| GP | dnbC. | EPIVAL CR 500mg m/r tablets |
| GP | dnbd. | EPILIM CHRONO 300 m/r tablets |
| GP | dnbD. | EPISENTA 300mg/3mL solution for injection |
| GP | dnbe. | EPILIM CHRONO 500 m/r tablets |
| GP | dnbE. | SODIUM VALPROATE 300mg/3mL solution for injection |
| GP | dnbF. | EPISENTA 150mg m/r capsules |
| GP | dnbG. | EPISENTA 300mg m/r capsules |
| GP | dnbH. | EPISENTA 500mg/sachet m/r granules |
| GP | dnbI. | EPISENTA 1g/sachet m/r granules |
| GP | dnbJ. | SODIUM VALPROATE 150mg m/r capsules |
| GP | dnbK. | SODIUM VALPROATE 300mg m/r capsules |
| GP | dnbL. | SODIUM VALPROATE 500mg/sachet m/r granules |
| GP | dnbM. | SODIUM VALPROATE 1g/sachet m/r granules |
| GP | dnbn. | SODIUM VALPROATE 50mg/sachet m/r granules |
| GP | dnbN. | SODIUM VALPROATE 1g/10mL solution for injection |
| GP | dnbo. | SODIUM VALPROATE 100mg/sachet m/r granules |
| GP | dnbO. | EPISENTA 1g/10mL solution for injection |
| GP | dnbp. | SODIUM VALPROATE 250mg/sachet m/r granules |
| GP | dnbP. | EPILIM CHRONOSPHERE 50mg/sachet m/r granules |
| GP | dnbq. | SODIUM VALPROATE 750mg/sachet m/r granules |
| GP | dnbQ. | EPILIM CHRONOSPHERE 100mg/sachet m/r granules |
| GP | dnbr. | SODIUM VALPROATE 200mg m/r tablets |
| GP | dnbR. | EPILIM CHRONOSPHERE 250mg/sachet m/r granules |
| GP | dnbs. | SODIUM VALPROATE 300mg m/r tablets |
| GP | dnbS. | EPILIM CHRONOSPHERE 500mg/sachet m/r granules |
| GP | dnbt. | SODIUM VALPROATE 500mg m/r tablets |
| GP | dnbT. | EPILIM CHRONOSPHERE 750mg/sachet m/r granules |
| GP | dnbu. | SODIUM VALPROATE 400mg/4mL injection |
| GP | dnbU. | EPILIM CHRONOSPHERE 1g/sachet m/r granules |
| GP | dnbv. | SODIUM VALPROATE 100mg crushable tablets |
| GP | dnbw. | SODIUM VALPROATE 200mg crushable tablets |
| GP | dnbx. | SODIUM VALPROATE 500mg tablets |
| GP | dnby. | SODIUM VALPROATE 200mg/5mL sugar free liquid |
| GP | dnbz. | SODIUM VALPROATE 200mg/5mL syrup |
| GP | dnc.. | CLOBAZAM [EPILEPSY ONLY] |
| GP | dnc1. | *CLOBAZAM SLS 10mg capsules |
| GP | dne.. | VIGABATRIN |
| GP | dne1. | VIGABATRIN 500mg tablets |
| GP | dne2. | SABRIL 500mg tablets |
| GP | dne3. | VIGABATRIN 500mg powder sachets |
| GP | dne4. | SABRIL 500mg powder sachets |
| GP | dnf.. | LAMOTRIGINE |
| GP | dnf1. | LAMOTRIGINE 50mg tablets |
| GP | dnf2. | LAMOTRIGINE 100mg tablets |
| GP | dnf3. | LAMICTAL 50mg tablets |
| GP | dnf4. | LAMICTAL 100mg tablets |
| GP | dnf5. | LAMOTRIGINE 25mg tablets |
| GP | dnf6. | LAMICTAL 25mg tablets |
| GP | dnf7. | LAMICTAL 5mg dispersible tablets |
| GP | dnf8. | LAMICTAL 25mg dispersible tablets |
| GP | dnf9. | LAMICTAL 100mg dispersible tablets |
| GP | dnfA. | LAMOTRIGINE 5mg dispersible tablets |
| GP | dnfB. | LAMOTRIGINE 25mg dispersible tablets |
| GP | dnfC. | LAMOTRIGINE 100mg dispersible tablets |
| GP | dnfD. | LAMICTAL 200mg tablets |
| GP | dnfE. | LAMOTRIGINE 200mg tablets |
| GP | dnfF. | LAMICTAL MONOTHERAPY 25mg starter pack |
| GP | dnfG. | LAMICTAL VALPROATE ADD-ON 25mg starter pack |
| GP | dnfH. | LAMICTAL NON-VALPROATE ADD-ON 50mg starter pack |
| GP | dnfJ. | LAMICTAL 2mg dispersible tablets |
| GP | dnfz. | LAMOTRIGINE 2mg dispersible tablets |
| GP | dng.. | PIRACETAM |
| GP | dng1. | NOOTROPIL 800mg tablets |
| GP | dng2. | NOOTROPIL 1.2g tablets |
| GP | dng3. | NOOTROPIL 33% oral solution |
| GP | dng4. | PIRACETAM 800mg tablets |
| GP | dng5. | PIRACETAM 1.2g tablets |
| GP | dng6. | PIRACETAM 333.3mg/mL oral solution |
| GP | dnh.. | VALPROIC ACID |
| GP | dnh1. | CONVULEX 150mg e/c capsules |
| GP | dnh2. | CONVULEX 300mg e/c capsules |
| GP | dnh3. | CONVULEX 500mg e/c capsules |
| GP | dnh4. | VALPROIC ACID 150mg e/c capsules |
| GP | dnh5. | VALPROIC ACID 300mg e/c capsules |
| GP | dnh6. | VALPROIC ACID 500mg e/c capsules |
| GP | dnh7. | DEPAKOTE 250mg e/c tablets |
| GP | dnh8. | DEPAKOTE 500mg e/c tablets |
| GP | dnhy. | VALPROIC ACID 500mg e/c tablets |
| GP | dnhz. | VALPROIC ACID 250mg e/c tablets |
| GP | dni.. | FOSPHENYTOIN SODIUM |
| GP | dni1. | FOSPHENYTOIN SODIUM 750mg/10mL injection concentrate |
| GP | dni2. | PRO-EPANUTIN 750mg/10mL injection concentrate |
| GP | dnj.. | GABAPENTIN |
| GP | dnj1. | GABAPENTIN 100mg capsules |
| GP | dnj2. | GABAPENTIN 300mg capsules |
| GP | dnj3. | GABAPENTIN 400mg capsules |
| GP | dnj4. | NEURONTIN 100mg capsules |
| GP | dnj5. | NEURONTIN 300mg capsules |
| GP | dnj6. | NEURONTIN 400mg capsules |
| GP | dnj7. | NEURONTIN 600mg tablets |
| GP | dnj8. | NEURONTIN 800mg tablets |
| GP | dnj9. | NEURONTIN 300mg capsules/600mg tablets titration pack |
| GP | dnjx. | GABAPENTIN 300mg capsules/600mg tablets titration pack |
| GP | dnjy. | GABAPENTIN 600mg tablets |
| GP | dnjz. | GABAPENTIN 800mg tablets |
| GP | dnk.. | TOPIRAMATE |
| GP | dnk1. | TOPIRAMATE 50mg tablets |
| GP | dnk2. | TOPIRAMATE 100mg tablets |
| GP | dnk3. | TOPIRAMATE 200mg tablets |
| GP | dnk4. | TOPAMAX 50mg tablets |
| GP | dnk5. | TOPAMAX 100mg tablets |
| GP | dnk6. | TOPAMAX 200mg tablets |
| GP | dnk7. | TOPIRAMATE 25mg tablets |
| GP | dnk8. | TOPAMAX 25mg tablets |
| GP | dnk9. | TOPIRAMATE 15mg beads in capsules |
| GP | dnkA. | TOPIRAMATE 25mg beads in capsules |
| GP | dnkB. | TOPAMAX SPRINKLE 15mg capsules |
| GP | dnkC. | TOPAMAX SPRINKLE 25mg capsules |
| GP | dnkD. | TOPIRAMATE 50mg beads in capsules |
| GP | dnkE. | TOPAMAX SPRINKLE 50mg capsules |
| GP | dnl.. | TIAGABINE |
| GP | dnl1. | TIAGABINE 5mg tablets |
| GP | dnl2. | TIAGABINE 10mg tablets |
| GP | dnl3. | TIAGABINE 15mg tablets |
| GP | dnl4. | GABITRIL 5mg tablets |
| GP | dnl5. | GABITRIL 10mg tablets |
| GP | dnl6. | GABITRIL 15mg tablets |
| GP | dnm.. | OXCARBAZEPINE |
| GP | dnm1. | TRILEPTAL 150 tablets |
| GP | dnm2. | TRILEPTAL 300 tablets |
| GP | dnm3. | TRILEPTAL 600 tablets |
| GP | dnm4. | TRILEPTAL 60mg/mL sugar free oral suspension |
| GP | dnmw. | OXCARBAZEPINE 60mg/mL sugar free oral suspension |
| GP | dnmx. | OXCARBAZEPINE 150mg tablets |
| GP | dnmy. | OXCARBAZEPINE 300mg tablets |
| GP | dnmz. | OXCARBAZEPINE 600mg tablets |
| GP | dno.. | LEVETIRACETAM |
| GP | dno1. | KEPPRA 250mg tablets |
| GP | dno2. | KEPPRA 500mg tablets |
| GP | dno3. | KEPPRA 1g tablets |
| GP | dno4. | KEPPRA 750mg tablets |
| GP | dno5. | KEPPRA 100mg/mL s/f oral solution |
| GP | dno6. | KEPPRA 500mg/5mL solution for injection |
| GP | dnou. | LEVETIRACETAM 500mg/5mL solution for injection |
| GP | dnov. | LEVETIRACETAM 100mg/mL s/f oral solution |
| GP | dnow. | LEVETIRACETAM 750mg tablets |
| GP | dnox. | LEVETIRACETAM 1g tablets |
| GP | dnoy. | LEVETIRACETAM 500mg tablets |
| GP | dnoz. | LEVETIRACETAM 250mg tablets |
| GP | dnp.. | PREGABALIN |
| GP | dnp1. | LYRICA 25mg capsules |
| GP | dnp2. | LYRICA 50mg capsules |
| GP | dnp3. | LYRICA 75mg capsules |
| GP | dnp4. | LYRICA 100mg capsules |
| GP | dnp5. | LYRICA 150mg capsules |
| GP | dnp6. | LYRICA 200mg capsules |
| GP | dnp7. | LYRICA 300mg capsules |
| GP | dnp8. | LYRICA 225mg capsules |
| GP | dnps. | PREGABALIN 225mg capsules |
| GP | dnpt. | PREGABALIN 300mg capsules |
| GP | dnpu. | PREGABALIN 200mg capsules |
| GP | dnpv. | PREGABALIN 100mg capsules |
| GP | dnpw. | PREGABALIN 150mg capsules |
| GP | dnpx. | PREGABALIN 75mg capsules |
| GP | dnpy. | PREGABALIN 50mg capsules |
| GP | dnpz. | PREGABALIN 25mg capsules |
| GP | dnq.. | ZONISAMIDE |
| GP | dnq1. | ZONISAMIDE 25mg capsules |
| GP | dnq2. | ZONISAMIDE 50mg capsules |
| GP | dnq3. | ZONISAMIDE 100mg capsules |
| GP | dnq4. | ZONEGRAN 25mg capsules |
| GP | dnq5. | ZONEGRAN 50mg capsules |
| GP | dnq6. | ZONEGRAN 100mg capsules |
| GP | dnr.. | RUFINAMIDE |
| GP | dnr1. | INOVELON 100mg tablets |
| GP | dnr2. | INOVELON 200mg tablets |
| GP | dnr3. | INOVELON 400mg tablets |
| GP | dnrx. | RUFINAMIDE 400mg tablets |
| GP | dnry. | RUFINAMIDE 200mg tablets |
| GP | dnrz. | RUFINAMIDE 100mg tablets |
| GP | dns.. | STIRIPENTOL |
| GP | dns1. | DIACOMIT 250mg capsules |
| GP | dns2. | DIACOMIT 500mg capsules |
| GP | dns3. | DIACOMIT 250mg/sachet powder for oral suspension |
| GP | dns4. | DIACOMIT 500mg/sachet powder for oral suspension |
| GP | dnsw. | STIRIPENDOL 500mg/sachet powder for oral suspension |
| GP | dnsx. | STIRIPENTOL 250mg/sachet powder for oral suspension |
| GP | dnsy. | STIRIPENTOL 500mg capsules |
| GP | dnsz. | STIRIPENTOL 250mg capsules |
| GP | dnt.. | LACOSAMIDE |
| GP | dnt1. | VIMPAT 200mg/20mL solution for injection |
| GP | dnt2. | VIMPAT 15mg/1mL sugar free liquid |
| GP | dnt3. | VIMPAT 50mg tablets |
| GP | dnt4. | VIMPAT 100mg tablets |
| GP | dnt5. | VIMPAT 150mg tablets |
| GP | dnt6. | VIMPAT 200mg tablets |
| GP | dnt7. | LACOSAMIDE 200mg/20mL solution for injection |
| GP | dnt8. | LACOSAMIDE 15mg/1mL sugar free liquid |
| GP | dnt9. | LACOSAMIDE 50mg tablets |
| GP | dntA. | LACOSAMIDE 100mg tablets |
| GP | dntB. | LACOSAMIDE 150mg tablets |
| GP | dntC. | LACOSAMIDE 200mg tablets |
| GP | dnu.. | ESLICARBAZEPINE |
| GP | dnu1. | ZEBINIX 800mg tablets |
| GP | dnu2. | ESLICARBAZEPINE ACETATE 800mg tablets |
| GP | dnv.. | RETIGABINE |
| GP | dnv1. | TROBALT 50mg tablets |
| GP | dnv2. | TROBALT 100mg tablets |
| GP | dnv3. | TROBALT 200mg tablets |
| GP | dnv4. | TROBALT 300mg tablets |
| GP | dnv5. | TROBALT 400mg tablets |
| GP | dnv6. | TROBALT tablets initiation pack |
| GP | dnv7. | RETIGABINE 50mg tablets |
| GP | dnv8. | RETIGABINE 100mg tablets |
| GP | dnv9. | RETIGABINE 200mg tablets |
| GP | dnvA. | RETIGABINE 300mg tablets |
| GP | dnvB. | RETIGABINE 400mg tablets |
| GP | dnvC. | RETIGABINE 50mg+100mg tablets initiation pack |
| GP | do... | STATUS EPILEPTICUS DRUGS |
| GP | do1.. | DIAZEPAM [EPILEPSY USE] |
| GP | do11. | DIAZEMULS [EP] 10mg/2mL injection |
| GP | do12. | STESOLID [EP] 10mg/2mL injection |
| GP | do13. | *STESOLID 20mg/4mL injection |
| GP | do14. | STESOLID 5mg/2.5mL rectal solution |
| GP | do15. | STESOLID 10mg/2.5mL rectal solution |
| GP | do16. | VALIUM [EP] 10mg/2mL injection |
| GP | do17. | VALIUM [EP] 20mg/4mL injection |
| GP | do18. | DIAZEPAM 5mg/2.5mL RecTubes |
| GP | do19. | DIAZEPAM 10mg/2.5mL RecTubes |
| GP | do1A. | DIAZEPAM 2.5mg/1.25mL RecTubes |
| GP | do1B. | *DIAZEPAM 20mg/5mL RecTubes |
| GP | do1t. | DIAZEPAM 2.5mg/1.25mL rectal solution |
| GP | do1u. | DIAZEPAM 20mg/5mL rectal solution |
| GP | do1v. | DIAZEPAM 10mg/2mL emulsion injection |
| GP | do1w. | DIAZEPAM 10mg/2mL injection |
| GP | do1x. | DIAZEPAM 5mg/2.5mL rectal solution |
| GP | do1y. | DIAZEPAM 10mg/2.5mL rectal solution |
| GP | do1z. | *DIAZEPAM 20mg/4mL injection |
| GP | do2.. | CLONAZEPAM [STATUS EPILEPSY] |
| GP | do21. | RIVOTRIL 1mg/1mL injection |
| GP | do2z. | CLONAZEPAM 1mg/1mL injection |
| GP | do3.. | CLOMETHIAZOLE EDISYLATE [CENTRAL NERVOUS SYSTEM USE] |
| GP | do31. | HEMINEVRIN [CNS] 8mg/mL intravenous infusion |
| GP | do3z. | CLOMETHIAZOLE EDISYLATE 8mg/mL intravenous infusion |
| GP | do4.. | LORAZEPAM [EPILEPSY] |
| GP | do41. | ATIVAN [EP] 4mg/mL injection |
| GP | do5.. | PARALDEHYDE |
| GP | do51. | *PARALDEHYDE injection 5mL |
| GP | do52. | *PARALDEHYDE injection 10mL |
| GP | do6.. | PHENYTOIN SODIUM [STATUS EPILEPSY] |
| GP | do61. | EPANUTIN [EP] 250mg/5mL injection |
| GP | do6z. | PHENYTOIN SODIUM 250mg/5mL injection |
| HOSPITAL ADMISSION | ICD10 G40% | Epilepsy |
|  |  |  |

Diabetes

| **GP** | **CODE** | **READ_DESC** |
| --- | --- | --- |
| GP | C10.. | Diabetes mellitus |
| GP | C100. | Diabetes mellitus with no mention of complication |
| GP | C1000 | Diabetes mellitus, juvenile type, no mention of complication |
| GP | C1001 | Diabetes mellitus, adult onset, no mention of complication |
| GP | C100z | Diabetes mellitus NOS with no mention of complication |
| GP | C101. | Diabetes mellitus with ketoacidosis |
| GP | C1010 | Diabetes mellitus, juvenile type, with ketoacidosis |
| GP | C1011 | Diabetes mellitus, adult onset, with ketoacidosis |
| GP | C101y | Other specified diabetes mellitus with ketoacidosis |
| GP | C101z | Diabetes mellitus NOS with ketoacidosis |
| GP | C102. | Diabetes mellitus with hyperosmolar coma |
| GP | C1020 | Diabetes mellitus, juvenile type, with hyperosmolar coma |
| GP | C1021 | Diabetes mellitus, adult onset, with hyperosmolar coma |
| GP | C102z | Diabetes mellitus NOS with hyperosmolar coma |
| GP | C103. | Diabetes mellitus with ketoacidotic coma |
| GP | C1030 | Diabetes mellitus, juvenile type, with ketoacidotic coma |
| GP | C1031 | Diabetes mellitus, adult onset, with ketoacidotic coma |
| GP | C103y | Other specified diabetes mellitus with coma |
| GP | C103z | Diabetes mellitus NOS with ketoacidotic coma |
| GP | C104. | Diabetes mellitus with renal manifestation |
| GP | C1040 | Diabetes mellitus, juvenile type, with renal manifestation |
| GP | C1041 | Diabetes mellitus, adult onset, with renal manifestation |
| GP | C104y | Other specified diabetes mellitus with renal complications |
| GP | C104z | Diabetes mellitus with nephropathy NOS |
| GP | C105. | Diabetes mellitus with ophthalmic manifestation |
| GP | C1050 | Diabetes mellitus, juvenile type, + ophthalmic manifestation |
| GP | C1051 | Diabetes mellitus, adult onset, + ophthalmic manifestation |
| GP | C105y | Other specified diabetes mellitus with ophthalmic complicatn |
| GP | C105z | Diabetes mellitus NOS with ophthalmic manifestation |
| GP | C106. | Diabetes mellitus with neurological manifestation |
| GP | C1060 | Diabetes mellitus, juvenile, + neurological manifestation |
| GP | C1061 | Diabetes mellitus, adult onset, + neurological manifestation |
| GP | C106y | Other specified diabetes mellitus with neurological comps |
| GP | C106z | Diabetes mellitus NOS with neurological manifestation |
| GP | C107. | Diabetes mellitus with peripheral circulatory disorder |
| GP | C1070 | Diabetes mellitus, juvenile +peripheral circulatory disorder |
| GP | C1071 | Diabetes mellitus, adult, + peripheral circulatory disorder |
| GP | C1072 | Diabetes mellitus, adult with gangrene |
| GP | C1073 | IDDM with peripheral circulatory disorder |
| GP | C1074 | NIDDM with peripheral circulatory disorder |
| GP | C107y | Other specified diabetes mellitus with periph circ comps |
| GP | C107z | Diabetes mellitus NOS with peripheral circulatory disorder |
| GP | C108. | Insulin dependent diabetes mellitus |
| GP | C1080 | Insulin-dependent diabetes mellitus with renal complications |
| GP | C1081 | Insulin-dependent diabetes mellitus with ophthalmic comps |
| GP | C1082 | Insulin-dependent diabetes mellitus with neurological comps |
| GP | C1083 | Insulin dependent diabetes mellitus with multiple complicatn |
| GP | C1084 | Unstable insulin dependent diabetes mellitus |
| GP | C1085 | Insulin dependent diabetes mellitus with ulcer |
| GP | C1086 | Insulin dependent diabetes mellitus with gangrene |
| GP | C1087 | Insulin dependent diabetes mellitus with retinopathy |
| GP | C1088 | Insulin dependent diabetes mellitus - poor control |
| GP | C1089 | Insulin dependent diabetes maturity onset |
| GP | C108A | Insulin-dependent diabetes without complication |
| GP | C108B | Insulin dependent diabetes mellitus with mononeuropathy |
| GP | C108C | Insulin dependent diabetes mellitus with polyneuropathy |
| GP | C108D | Insulin dependent diabetes mellitus with nephropathy |
| GP | C108E | Insulin dependent diabetes mellitus with hypoglycaemic coma |
| GP | C108F | Insulin dependent diabetes mellitus with diabetic cataract |
| GP | C108G | Insulin dependent diab mell with peripheral angiopathy |
| GP | C108H | Insulin dependent diabetes mellitus with arthropathy |
| GP | C108J | Insulin dependent diab mell with neuropathic arthropathy |
| GP | C108y | Other specified diabetes mellitus with multiple comps |
| GP | C108z | Unspecified diabetes mellitus with multiple complications |
| GP | C109. | Non-insulin dependent diabetes mellitus |
| GP | C1090 | Non-insulin-dependent diabetes mellitus with renal comps |
| GP | C1091 | Non-insulin-dependent diabetes mellitus with ophthalm comps |
| GP | C1092 | Non-insulin-dependent diabetes mellitus with neuro comps |
| GP | C1093 | Non-insulin-dependent diabetes mellitus with multiple comps |
| GP | C1094 | Non-insulin dependent diabetes mellitus with ulcer |
| GP | C1095 | Non-insulin dependent diabetes mellitus with gangrene |
| GP | C1096 | Non-insulin-dependent diabetes mellitus with retinopathy |
| GP | C1097 | Non-insulin dependent diabetes mellitus - poor control |
| GP | C1099 | Non-insulin-dependent diabetes mellitus without complication |
| GP | C109A | Non-insulin dependent diabetes mellitus with mononeuropathy |
| GP | C109B | Non-insulin dependent diabetes mellitus with polyneuropathy |
| GP | C109C | Non-insulin dependent diabetes mellitus with nephropathy |
| GP | C109D | Non-insulin dependent diabetes mellitus with hypoglyca coma |
| GP | C109E | Non-insulin depend diabetes mellitus with diabetic cataract |
| GP | C109F | Non-insulin-dependent d m with peripheral angiopath |
| GP | C109G | Non-insulin dependent diabetes mellitus with arthropathy |
| GP | C109H | Non-insulin dependent d m with neuropathic arthropathy |
| GP | C109J | Insulin treated Type 2 diabetes mellitus |
| GP | C109K | Hyperosmolar non-ketotic state in type 2 diabetes mellitus |
| GP | C10A. | Malnutrition-related diabetes mellitus |
| GP | C10A0 | Malnutrition-related diabetes mellitus with coma |
| GP | C10A1 | Malnutrition-related diabetes mellitus with ketoacidosis |
| GP | C10A2 | Malnutrition-related diabetes mellitus with renal complicatn |
| GP | C10A3 | Malnutrit-related diabetes mellitus wth ophthalmic complicat |
| GP | C10A4 | Malnutrition-related diabetes mellitus wth neuro complicatns |
| GP | C10A5 | Malnutritn-relat diabetes melitus wth periph circul complctn |
| GP | C10A6 | Malnutrition-related diabetes mellitus with multiple comps |
| GP | C10A7 | Malnutrition-related diabetes mellitus without complications |
| GP | C10AW | Malnutrit-related diabetes mellitus with unspec complics |
| GP | C10AX | Malnutrit-relat diabetes mellitus with other spec comps |
| GP | C10B. | Diabetes mellitus induced by steroids |
| GP | C10B0 | Steroid induced diabetes mellitus without complication |
| GP | C10C. | Diabetes mellitus autosomal dominant |
| GP | C10C. | Diabetes mellitus autosomal dominant |
| GP | C10D. | Diabetes mellitus autosomal dominant type 2 |
| GP | C10D. | Diabetes mellitus autosomal dominant type 2 |
| GP | C10E. | Type 1 diabetes mellitus |
| GP | C10E. | Type 1 diabetes mellitus |
| GP | C10E0 | Type 1 diabetes mellitus with renal complications |
| GP | C10E0 | Type 1 diabetes mellitus with renal complications |
| GP | C10E1 | Type 1 diabetes mellitus with ophthalmic complications |
| GP | C10E1 | Type 1 diabetes mellitus with ophthalmic complications |
| GP | C10E2 | Type 1 diabetes mellitus with neurological complications |
| GP | C10E3 | Type 1 diabetes mellitus with multiple complications |
| GP | C10E3 | Type 1 diabetes mellitus with multiple complications |
| GP | C10E4 | Unstable type 1 diabetes mellitus |
| GP | C10E5 | Type 1 diabetes mellitus with ulcer |
| GP | C10E6 | Type 1 diabetes mellitus with gangrene |
| GP | C10E7 | Type 1 diabetes mellitus with retinopathy |
| GP | C10E8 | Type 1 diabetes mellitus - poor control |
| GP | C10E8 | Type 1 diabetes mellitus - poor control |
| GP | C10E9 | Type 1 diabetes mellitus maturity onset |
| GP | C10E9 | Type 1 diabetes mellitus maturity onset |
| GP | C10EA | Type 1 diabetes mellitus without complication |
| GP | C10EB | Type 1 diabetes mellitus with mononeuropathy |
| GP | C10EC | Type 1 diabetes mellitus with polyneuropathy |
| GP | C10ED | Type 1 diabetes mellitus with nephropathy |
| GP | C10EE | Type 1 diabetes mellitus with hypoglycaemic coma |
| GP | C10EF | Type 1 diabetes mellitus with diabetic cataract |
| GP | C10EG | Type 1 diabetes mellitus with peripheral angiopathy |
| GP | C10EG | Type 1 diabetes mellitus with peripheral angiopathy |
| GP | C10EH | Type 1 diabetes mellitus with arthropathy |
| GP | C10EJ | Type 1 diabetes mellitus with neuropathic arthropathy |
| GP | C10EK | Type 1 diabetes mellitus with persistent proteinuria |
| GP | C10EL | Type 1 diabetes mellitus with persistent microalbuminuria |
| GP | C10EM | Type 1 diabetes mellitus with ketoacidosis |
| GP | C10EN | Type 1 diabetes mellitus with ketoacidotic coma |
| GP | C10EP | Type 1 diabetes mellitus with exudative maculopathy |
| GP | C10EQ | Type 1 diabetes mellitus with gastroparesis |
| GP | C10ER | Latent autoimmune diabetes mellitus in adult |
| GP | C10F. | Type 2 diabetes mellitus |
| GP | C10F. | Type 2 diabetes mellitus |
| GP | C10F0 | Type 2 diabetes mellitus with renal complications |
| GP | C10F1 | Type 2 diabetes mellitus with ophthalmic complications |
| GP | C10F1 | Type 2 diabetes mellitus with ophthalmic complications |
| GP | C10F2 | Type 2 diabetes mellitus with neurological complications |
| GP | C10F2 | Type 2 diabetes mellitus with neurological complications |
| GP | C10F3 | Type 2 diabetes mellitus with multiple complications |
| GP | C10F4 | Type 2 diabetes mellitus with ulcer |
| GP | C10F5 | Type 2 diabetes mellitus with gangrene |
| GP | C10F6 | Type 2 diabetes mellitus with retinopathy |
| GP | C10F7 | Type 2 diabetes mellitus - poor control |
| GP | C10F7 | Type 2 diabetes mellitus - poor control |
| GP | C10F9 | Type 2 diabetes mellitus without complication |
| GP | C10FA | Type 2 diabetes mellitus with mononeuropathy |
| GP | C10FB | Type 2 diabetes mellitus with polyneuropathy |
| GP | C10FC | Type 2 diabetes mellitus with nephropathy |
| GP | C10FD | Type 2 diabetes mellitus with hypoglycaemic coma |
| GP | C10FD | Type 2 diabetes mellitus with hypoglycaemic coma |
| GP | C10FE | Type 2 diabetes mellitus with diabetic cataract |
| GP | C10FF | Type 2 diabetes mellitus with peripheral angiopathy |
| GP | C10FF | Type 2 diabetes mellitus with peripheral angiopathy |
| GP | C10FG | Type 2 diabetes mellitus with arthropathy |
| GP | C10FH | Type 2 diabetes mellitus with neuropathic arthropathy |
| GP | C10FH | Type 2 diabetes mellitus with neuropathic arthropathy |
| GP | C10FJ | Insulin treated Type 2 diabetes mellitus |
| GP | C10FJ | Insulin treated Type 2 diabetes mellitus |
| GP | C10FK | Hyperosmolar non-ketotic state in type 2 diabetes mellitus |
| GP | C10FL | Type 2 diabetes mellitus with persistent proteinuria |
| GP | C10FM | Type 2 diabetes mellitus with persistent microalbuminuria |
| GP | C10FN | Type 2 diabetes mellitus with ketoacidosis |
| GP | C10FP | Type 2 diabetes mellitus with ketoacidotic coma |
| GP | C10FQ | Type 2 diabetes mellitus with exudative maculopathy |
| GP | C10FR | Type 2 diabetes mellitus with gastroparesis |
| GP | C10FS | Maternally inherited diabetes mellitus |
| GP | C10G. | Secondary pancreatic diabetes mellitus |
| GP | C10G0 | Secondary pancreatic diabetes mellitus without complication |
| GP | C10H. | Diabetes mellitus induced by non-steroid drugs |
| GP | C10H0 | DM induced by non-steroid drugs without complication |
| GP | C10J. | Insulin autoimmune syndrome |
| GP | C10J0 | Insulin autoimmune syndrome without complication |
| GP | C10K. | Type A insulin resistance |
| GP | C10K0 | Type A insulin resistance without complication |
| GP | C10L. | Fibrocalculous pancreatopathy |
| GP | C10L0 | Fibrocalculous pancreatopathy without complication |
| GP | C10M. | Lipoatrophic diabetes mellitus |
| GP | C10M0 | Lipoatrophic diabetes mellitus without complication |
| GP | C10N. | Secondary diabetes mellitus |
| GP | C10N0 | Secondary diabetes mellitus without complication |
| GP | C10N1 | Cystic fibrosis related diabetes mellitus |
| GP | C10y. | Diabetes mellitus with other specified manifestation |
| GP | C10y0 | Diabetes mellitus, juvenile, + other specified manifestation |
| GP | C10y1 | Diabetes mellitus, adult, + other specified manifestation |
| GP | C10yy | Other specified diabetes mellitus with other spec comps |
| GP | C10yz | Diabetes mellitus NOS with other specified manifestation |
| GP | C10z. | Diabetes mellitus with unspecified complication |
| GP | C10z0 | Diabetes mellitus, juvenile type, + unspecified complication |
| GP | C10z1 | Diabetes mellitus, adult onset, + unspecified complication |
| GP | C10zy | Other specified diabetes mellitus with unspecified comps |
| GP | C10zz | Diabetes mellitus NOS with unspecified complication |
| GP | f1... | SHORT-ACTING INSULIN PREPARATIONS |
| GP | f11.. | *SOLUBLE INSULIN |
| GP | f111. | INSULIN 100iu/mL injection 10mL |
| GP | f112. | *HYPURIN injection 10mL |
| GP | f12.. | SOLUBLE NEUTRAL INSULIN |
| GP | f121. | NEUTRAL INSULIN 100iu/mL injection 10mL |
| GP | f122. | HYPURIN NEUTRAL 100iu/mL injection 10mL |
| GP | f123. | *NEUSULIN 100iu/mL injection |
| GP | f124. | *QUICKSOL 100iu/mL injection |
| GP | f125. | VELOSULIN 100iu/mL injection 10mL |
| GP | f126. | VELOSULIN CARTRIDGE 100iu/mL injection 5.7mL |
| GP | f127. | HUMAN ACTRAPID 100iu/mL injection 10mL |
| GP | f128. | HUMAN ACTRAPID 100iu/mL penfill cartridges |
| GP | f129. | HUMAN VELOSULIN 100iu/mL injection 10mL |
| GP | f12a. | HUMULIN S 100iu/mL injection 10mL |
| GP | f12A. | HUMAN ACTRAPID 100iu/mL penfill cartridges 1.5mL |
| GP | f12b. | *NOVOPEN |
| GP | f12B. | HUMAN ACTRAPID 100iu/mL preloaded injection pen 3mL |
| GP | f12c. | *PENJECT |
| GP | f12C. | HUMAJECT S 100iu/mL prefilled pen |
| GP | f12d. | PUR-IN NEUTRAL 100iu/mL vials 10mL |
| GP | f12D. | PORK VELOSULIN 100units/mL injection 10mL |
| GP | f12e. | PUR-IN NEUTRAL 100iu/mL cartridges 3mL |
| GP | f12E. | NEUTRAL INSULIN 100iu/mL injection cartridge |
| GP | f12f. | *AUTOPEN |
| GP | f12F. | HYPURIN BOVINE NEUTRAL 100iu/mL injection cartridge 1.5mL |
| GP | f12g. | HUMULIN S 100iu/mL cartridges 1.5mL |
| GP | f12G. | HYPURIN PORCINE NEUTRAL 100iu/mL injection cartridge 1.5mL |
| GP | f12h. | *NOVOPEN II device |
| GP | f12H. | HYPURIN BOVINE NEUTRAL 100iu/mL injection 10mL |
| GP | f12i. | *BD PEN device |
| GP | f12I. | HYPURIN PORCINE NEUTRAL 100iu/mL injection 10mL |
| GP | f12j. | *PUR-IN PEN device |
| GP | f12J. | ACTRAPID (HUMAN) PENFILL 100iu/mL cartridges 3mL |
| GP | f12k. | *PUR-IN PEN 1 device |
| GP | f12K. | PORK ACTRAPID 100iu/mL injection 10mL |
| GP | f12L. | INSUMAN RAPID 100iu/mL injection vials 5mL |
| GP | f12m. | *PUR-IN PEN 2 device |
| GP | f12M. | INSUMAN RAPID 100iu/mL injection cartridge 3mL |
| GP | f12n. | *PUR-IN PEN 4 device |
| GP | f12N. | BD ULTRA PEN 3.0mL 1 unit device |
| GP | f12p. | *DIAPEN 1 device |
| GP | f12P. | BD ULTRA PEN 1.5mL 1 unit device |
| GP | f12q. | *DIAPEN 2 device |
| GP | f12Q. | INSUMAN RAPID OPTISET 100iu/mL prefilled pen 3mL |
| GP | f12r. | *NOVOPEN I device |
| GP | f12R. | HUMAN VELOSULIN (PYR) 100iu/mL injection 10mL |
| GP | f12s. | HUMULIN S 100iu/mL cartridges 3mL |
| GP | f12S. | HYPURIN BOVINE NEUTRAL 100iu/mL injection cartridge 3mL |
| GP | f12t. | *AUTOPEN 1.5mL one unit device |
| GP | f12T. | HYPURIN PORCINE NEUTRAL 100iu/mL injection cartridge 3mL |
| GP | f12u. | *AUTOPEN 1.5mL two unit device |
| GP | f12U. | EXUBERA 1mg powder for inhalation |
| GP | f12v. | *AUTOPEN 3mL two unit device |
| GP | f12V. | EXUBERA 3mg powder for inhalation |
| GP | f12W. | HUMAN INSULIN 1mg powder for inhalation |
| GP | f12X. | HUMAN INSULIN 3mg powder for inhalation |
| GP | f12y. | HUMAN INSULIN 100units/mL injection cartridge |
| GP | f12z. | HUMAN INSULIN 100iu/mL injection vials |
| GP | f13.. | INSULIN LISPRO |
| GP | f131. | INSULIN LISPRO 100iu/mL vials |
| GP | f132. | HUMALOG 100iu/mL injection 10mL |
| GP | f133. | INSULIN LISPRO 100iu/mL cartridges |
| GP | f134. | HUMALOG 100iu/mL cartridges 1.5mL |
| GP | f135. | HUMALOG 100iu/mL cartridges 3mL |
| GP | f136. | INSULIN LISPRO 100iu/mL prefilled pen |
| GP | f137. | HUMALOG-PEN 100iu/mL prefilled pen 3mL |
| GP | f138. | HUMALOG KWIKPEN 100iu/mL prefilled pen 3mL |
| GP | f14.. | INSULIN ASPART |
| GP | f141. | NOVORAPID 100units/mL injection vial |
| GP | f142. | NOVORAPID NOVOLET 100units/mL prefilled syringe 3mL |
| GP | f143. | NOVORAPID PENFILL 100units/mL cartridges 3mL |
| GP | f144. | NOVORAPID FLEXPEN 100units/mL prefilled pen 3mL |
| GP | f145. | NOVORAPID FLEXTOUCH 100units/mL soln for injection pen 3mL |
| GP | f14w. | INSULIN ASPART 100units/mL prefilled pen |
| GP | f14x. | INSULIN ASPART 100units/mL injection vial |
| GP | f14y. | INSULIN ASPART 100units/mL prefilled syringe |
| GP | f14z. | INSULIN ASPART 100units/mL cartridges |
| GP | f15.. | INSULIN GLULISINE |
| GP | f151. | APIDRA 100iu/mL injection vials 10mL |
| GP | f152. | APIDRA 100iu/mL injection cartridges 3mL |
| GP | f153. | APIDRA 100iu/mL OptiSet prefilled pen 3mL |
| GP | f154. | APIDRA 100iu/mL OptiClik cartridges 3mL |
| GP | f155. | APIDRA 100iu/mL SoloStar prefilled pen 3mL |
| GP | f15x. | INSULIN GLULISINE 100iu/mL prefilled pen |
| GP | f15y. | INSULIN GLULISINE 100iu/mL injection cartridges |
| GP | f15z. | INSULIN GLULISINE 100iu/mL injection vials |
| GP | f2... | MEDIUM/LONG-ACTING INSULINS |
| GP | f21.. | BIPHASIC INSULIN |
| GP | f211. | RAPITARD MC 100iu/mL injection 10mL |
| GP | f212. | *PENMIX cartridges 1.5mL |
| GP | f22.. | INSULIN ZINC SUSPENSION |
| GP | f221. | INSULIN ZINC LENTE 100iu/mL injection 10mL |
| GP | f222. | HYPURIN LENTE 100iu/mL injection 10mL |
| GP | f223. | LENTARD MC 100iu/mL injection 10mL |
| GP | f224. | *NEULENTE 100iu/mL injection |
| GP | f225. | *TEMPULIN 100iu/mL injection |
| GP | f226. | HUMAN MONOTARD 100iu/mL injection 10mL |
| GP | f227. | HUMULIN LENTE 100iu/mL injection 10mL |
| GP | f228. | HYPURIN BOVINE LENTE 100iu/mL injection 10mL |
| GP | f23.. | INSULIN ZINC SUSPENSION - AMORPHOUS |
| GP | f231. | SEMITARD MC 100iu/mL injection 10mL |
| GP | f24.. | INSULIN ZINC SUSPENSION - CRYSTALLINE |
| GP | f241. | HUMAN ULTRATARD 100iu/mL injection 10mL |
| GP | f242. | HUMULIN ZN 100iu/mL injection 10mL |
| GP | f25.. | ISOPHANE INSULIN |
| GP | f251. | ISOPHANE INSULIN 100iu/mL injection 10mL |
| GP | f252. | HYPURIN ISOPHANE 100iu/mL injection 10mL |
| GP | f253. | INSULATARD 100iu/mL injection 10mL |
| GP | f254. | *MONOPHANE 100iu/mL injection |
| GP | f255. | *NEUPHANE 100iu/mL injection |
| GP | f256. | *INITARD 50/50 injection 10mL |
| GP | f257. | *MIXTARD injection 10mL |
| GP | f258. | HUMAN INSULATARD 100iu/mL injection 10mL |
| GP | f259. | HUMAN PROTAPHANE 100iu/mL injection 10mL |
| GP | f25a. | HUMULIN I 100iu/mL injection 10mL |
| GP | f25A. | HUMULIN I 100iu/mL prefilled pen 3mL |
| GP | f25b. | HUMAN ACTRAPHANE injection 10mL |
| GP | f25B. | INSUMAN BASAL OPTISET 100iu/mL prefilled pen 3mL |
| GP | f25c. | *HUMAN INITARD injection 10mL |
| GP | f25C. | INSULATARD INNOLET 100units/mL prefilled syringe 3mL |
| GP | f25d. | *HUMAN MIXTARD injection 10mL |
| GP | f25D. | INSULATARD FLEXPEN 100iu/mL prefilled pen 3mL |
| GP | f25e. | *HUMULIN M1 injection 10mL |
| GP | f25E. | HYPURIN BOVINE ISOPHANE 100iu/mL injection cartridge 3mL |
| GP | f25f. | *HUMULIN M2 injection 10mL |
| GP | f25F. | HYPURIN PORCINE ISOPHANE 100iu/mL injection cartridge 3mL |
| GP | f25g. | *HUMULIN M3 injection 10mL |
| GP | f25G. | HUMULIN I KWIKPEN 100iu/mL prefilled pen 3mL |
| GP | f25h. | *HUMULIN M4 injection 10mL |
| GP | f25H. | INSUMAN BASAL SOLOSTAR 100iu/mL prefilled pen 3mL |
| GP | f25i. | HUMAN PROTAPHANE penfill 1.5mL |
| GP | f25j. | PUR-IN ISOPHANE 100iu/mL vials 10mL |
| GP | f25k. | PUR-IN ISOPHANE 100iu/mL cartridges 3mL |
| GP | f25l. | HUMULIN I 100iu/mL cartridges 1.5mL |
| GP | f25m. | HUMAN INSULATARD 100iu/mL preloaded injection pen 3mL |
| GP | f25n. | HUMAJECT I 100iu/mL prefilled pen |
| GP | f25o. | HUMULIN I 100iu/mL cartridges 3mL |
| GP | f25p. | HUMAN INSULATARD ge injection 10mL |
| GP | f25q. | HUMAN INSULATARD PENFILL cartridges 1.5mL |
| GP | f25r. | PORK INSULATARD 100units/mL injection 10mL |
| GP | f25s. | ISOPHANE INSULIN 100iu/mL injection cartridge |
| GP | f25t. | HYPURIN BOVINE ISOPHANE 100iu/mL injection cartridge 1.5mL |
| GP | f25u. | HYPURIN PORCINE ISOPHANE 100iu/mL injection cartridge 1.5mL |
| GP | f25v. | HYPURIN BOVINE ISOPHANE 100iu/mL injection 10mL |
| GP | f25w. | HYPURIN PORCINE ISOPHANE 100iu/mL injection 10mL |
| GP | f25W. | HUMAN ISOPHANE INSULIN 100units/mL prefilled syringe |
| GP | f25x. | INSULATARD (HUMAN) PENFILL cartridges 3mL |
| GP | f25X. | HUMAN ISOPHANE INSULIN 100units/mL injection cartridge |
| GP | f25y. | INSUMAN BASAL 100iu/mL injection vials 5mL |
| GP | f25Y. | HUMAN ISOPHANE INSULIN 100units/mL injection vials |
| GP | f25z. | INSUMAN BASAL 100iu/mL injection cartridge 3mL |
| GP | f26.. | PROTAMINE ZINC INSULIN |
| GP | f261. | HYPURIN PROTAMINE ZINC injection 10mL |
| GP | f262. | HYPURIN BOVINE PROTAMINE ZINC 100iu/mL injection 10mL |
| GP | f27.. | BIPHASIC ISOPHANE INSULIN |
| GP | f271. | *MIXTARD 30/70 injection 10mL |
| GP | f272. | *PENMIX 30/70 cartridges 1.5mL |
| GP | f273. | *PUR-IN MIX 15/85 vials 10mL |
| GP | f274. | PUR-IN MIX 15/85 cartridges 3mL |
| GP | f275. | *PUR-IN MIX 25/75 vials 10mL |
| GP | f276. | PUR-IN MIX 25/75 cartridges 3mL |
| GP | f277. | *PUR-IN MIX 50/50 vials 10mL |
| GP | f278. | PUR-IN MIX 50/50 cartridges 3mL |
| GP | f279. | *HUMULIN M1 10/90 vials 10mL |
| GP | f27a. | HUMULIN M1 10/90 cartridges 1.5mL |
| GP | f27A. | HUMAJECT M1 100iu/mL prefilled pen |
| GP | f27b. | *HUMULIN M2 20/80 vials 10mL |
| GP | f27B. | HUMAJECT M2 100iu/mL prefilled pen |
| GP | f27c. | HUMULIN M2 20/80 cartridges 1.5mL |
| GP | f27C. | HUMAJECT M3 100iu/mL prefilled pen |
| GP | f27d. | HUMULIN M3 30/70 vials 10mL |
| GP | f27D. | HUMAJECT M4 100iu/mL prefilled pen |
| GP | f27e. | HUMULIN M3 30/70 cartridges 1.5mL |
| GP | f27E. | HUMAJECT M5 100iu/mL prefilled pen |
| GP | f27f. | *HUMULIN M4 40/60 vials 10mL |
| GP | f27F. | HUMULIN M4 40/60 cartridges 3mL |
| GP | f27g. | HUMULIN M4 40/60 cartridges 1.5mL |
| GP | f27G. | HUMULIN M5 50/50 cartridges 3mL |
| GP | f27h. | *INITARD 50/50 injection 10mL |
| GP | f27H. | HUMAN MIXTARD 30 ge injection 10mL |
| GP | f27i. | HUMAN ACTRAPHANE 30/70 injection 10mL |
| GP | f27I. | HYPURIN PORCINE BIPHASIC ISOPHANE 30/70 injection cart 3mL |
| GP | f27j. | HUMAN MIXTARD 30/70 injection 10mL |
| GP | f27J. | HUMAN MIXTARD 10 PENFILL cartridges 1.5mL |
| GP | f27k. | HUMAN INITARD 50/50 injection 10mL |
| GP | f27K. | HUMAN MIXTARD 20 PENFILL cartridges 1.5mL |
| GP | f27l. | *PENMIX 10/90 cartridges 1.5mL |
| GP | f27L. | HUMAN MIXTARD 30 PENFILL cartridges 1.5mL |
| GP | f27m. | *PENMIX 20/80 cartridges 1.5mL |
| GP | f27M. | HUMAN MIXTARD 40 PENFILL cartridges 1.5mL |
| GP | f27n. | *PENMIX 40/60 cartridges 1.5mL |
| GP | f27N. | HUMAN MIXTARD 50 PENFILL cartridges 1.5mL |
| GP | f27o. | *PENMIX 50/50 cartridges 1.5mL |
| GP | f27O. | MIXTARD 30 INNOLET 100units/mL prefilled syringe 3mL |
| GP | f27p. | PENMIX 30/70 preloaded injection pen |
| GP | f27P. | HUMAN MIXTARD 10 prefilled pen |
| GP | f27q. | PENMIX 10/90 preloaded injection pen |
| GP | f27Q. | HUMAN MIXTARD 20 prefilled pen |
| GP | f27r. | PENMIX 20/80 preloaded injection pen |
| GP | f27R. | HUMAN MIXTARD 30 prefilled pen |
| GP | f27s. | PENMIX 40/60 preloaded injection pen |
| GP | f27S. | HUMAN MIXTARD 40 prefilled pen |
| GP | f27t. | PENMIX 50/50 preloaded injection pen |
| GP | f27T. | HUMAN MIXTARD 50 prefilled pen |
| GP | f27u. | *HUMULIN M5 50/50 vials 10mL |
| GP | f27v. | HUMULIN M5 50/50 cartridges 1.5mL |
| GP | f27V. | PORK MIXTARD 30 100units/mL injection 10mL |
| GP | f27w. | HUMULIN M1 10/90 cartridges 3mL |
| GP | f27W. | *HUMAN MIXTARD 50 vials 10mL |
| GP | f27x. | HUMULIN M2 20/80 cartridges 3mL |
| GP | f27X. | HYPURIN PORCINE BIPHASIC ISOPHANE 30/70 injection 10mL |
| GP | f27y. | INSUMAN COMB 25 100iu/mL injection cartridge 3mL |
| GP | f27Y. | HYPURIN PORCINE BIPHASIC ISOPHANE 30/70 injection cart 1.5mL |
| GP | f27z. | HUMULIN M3 30/70 cartridges 3mL |
| GP | f27Z. | INSUMAN COMB 25 100iu/mL injection vials 5mL |
| GP | f28.. | BIPHASIC ISOPHANE INSULIN 2 |
| GP | f281. | MIXTARD 10 (HUMAN) PENFILL cartridges 3mL |
| GP | f282. | MIXTARD 20 (HUMAN) PENFILL cartridges 3mL |
| GP | f283. | MIXTARD 30 (HUMAN) PENFILL cartridges 3mL |
| GP | f284. | MIXTARD 40 (HUMAN) PENFILL cartridges 3mL |
| GP | f285. | MIXTARD 50 (HUMAN) PENFILL cartridges 3mL |
| GP | f286. | INSUMAN COMB 15 100iu/mL injection cartridge 3mL |
| GP | f287. | INSUMAN COMB 50 100iu/mL injection vials 5mL |
| GP | f288. | INSUMAN COMB 15 OPTISET 100iu/mL prefilled pen 3mL |
| GP | f289. | INSUMAN COMB 25 OPTISET 100iu/mL prefilled pen 3mL |
| GP | f28A. | INSUMAN COMB 50 OPTISET 100iu/mL prefilled pen 3mL |
| GP | f28B. | INSUMAN COMB 15 100iu/mL injection vials 5mL |
| GP | f28C. | INSUMAN COMB 50 100iu/mL injection cartridge 3mL |
| GP | f28D. | HUMULIN M3 100iu/mL prefilled pen 3mL |
| GP | f28E. | HUMULIN M3 KWIKPEN 100iu/mL prefilled pen 3mL |
| GP | f28F. | INSUMAN COMB 25 SOLOSTAR 100iu/mL prefilled pen 3mL |
| GP | f29.. | INSULIN GLARGINE |
| GP | f291. | INSULIN GLARGINE 100iu/mL injection cartridges |
| GP | f292. | INSULIN GLARGINE 100iu/mL injection vials |
| GP | f293. | INSULIN GLARGINE 100iu/mL prefilled pen |
| GP | f294. | LANTUS 100iu/mL injection cartridges 3mL |
| GP | f295. | LANTUS 100iu/mL injection vials 10mL |
| GP | f296. | LANTUS 100iu/mL OptiSet prefilled pen 3mL |
| GP | f297. | LANTUS 100iu/mL OptiClik cartridges 3mL |
| GP | f298. | LANTUS 100iu/mL SoloStar prefilled pen 3mL |
| GP | f2A.. | INSULIN DETEMIR |
| GP | f2A1. | LEVEMIR PENFILL 100iu/mL injection cartridges 3mL |
| GP | f2A2. | LEVEMIR FLEXPEN 100iu/mL prefilled pen 3mL |
| GP | f2A3. | LEVEMIR INNOLET 100iu/mL prefilled syringe 3mL |
| GP | f2Ax. | INSULIN DETEMIR 100iu/mL prefilled syringe |
| GP | f2Ay. | INSULIN DETEMIR 100iu/mL prefilled pen |
| GP | f2Az. | INSULIN DETEMIR 100iu/mL injection cartridges |
| GP | fw... | SHORT WITH INTERMEDIATE-ACTING INSULINS |
| GP | fw1.. | BIPHASIC ISOPHANE INSULIN LISPRO |
| GP | fw11. | HUMALOG MIX25 100iu/mL cartridges 3mL |
| GP | fw12. | HUMALOG MIX25 100iu/mL prefilled pen 3mL |
| GP | fw13. | HUMALOG MIX50 100iu/mL prefilled pen 3mL |
| GP | fw14. | HUMALOG MIX50 100iu/mL cartridges 3mL |
| GP | fw15. | HUMALOG MIX25 KWIKPEN 100iu/mL prefilled pen 3mL |
| GP | fw16. | HUMALOG MIX50 KWIKPEN 100iu/mL prefilled pen 3mL |
| GP | fw2.. | BIPHASIC INSULIN ASPART |
| GP | fw21. | NOVOMIX 30 PENFILL 100units/mL injection cartridges 3mL |
| GP | fw22. | NOVOMIX 30 FLEXPEN 100units/mL injection prefilled pen 3mL |
| GP | f3... | SULPHONYLUREAS |
| GP | f31.. | *ACETOHEXAMIDE |
| GP | f311. | *DIMELOR 500mg tablets |
| GP | f31z. | *ACETOHEXAMIDE 500mg tablets |
| GP | f32.. | CHLORPROPAMIDE |
| GP | f321. | CHLORPROPAMIDE 100mg tablets |
| GP | f322. | CHLORPROPAMIDE 250mg tablets |
| GP | f323. | *DIABINESE 100mg tablets |
| GP | f324. | *DIABINESE 250mg tablets |
| GP | f325. | GLYMESE 250mg tablets |
| GP | f33.. | GLIBENCLAMIDE |
| GP | f331. | GLIBENCLAMIDE 2.5mg tablets |
| GP | f332. | GLIBENCLAMIDE 5mg tablets |
| GP | f333. | *DAONIL 5mg tablets |
| GP | f334. | *SEMI-DAONIL 2.5mg tablets |
| GP | f335. | *EUGLUCON 2.5mg tablets |
| GP | f336. | *EUGLUCON 5mg tablets |
| GP | f337. | *LIBANIL 2.5mg tablets |
| GP | f338. | *LIBANIL 5mg tablets |
| GP | f339. | *MALIX 2.5mg tablets |
| GP | f33a. | *MALIX 5mg tablets |
| GP | f33b. | *DAONIL CP 5mg tablets |
| GP | f33c. | *SEMI-DAONIL CP 2.5mg tablets |
| GP | f33d. | *DIABETAMIDE 2.5mg tablets |
| GP | f33e. | *DIABETAMIDE 5mg tablets |
| GP | f33f. | *CALABREN 2.5mg tablets |
| GP | f33g. | *CALABREN 5mg tablets |
| GP | f34.. | *GLIBORNURIDE |
| GP | f341. | *GLUTRIL 25mg tablets |
| GP | f34z. | *GLIBORNURIDE 25mg tablets |
| GP | f35.. | GLICLAZIDE |
| GP | f351. | DIAMICRON 80mg tablets |
| GP | f352. | DIAGLYK 80mg tablets |
| GP | f353. | VIVAZIDE 80mg tablets |
| GP | f354. | DIAMICRON MR 30mg m/r tablets |
| GP | f355. | NAZDOL MR 30mg m/r tablets |
| GP | f356. | EDICIL MR 30mg m/r tablets |
| GP | f357. | ZICRON 40mg tablets |
| GP | f358. | VITILE XL 30mg m/r tablets |
| GP | f35x. | GLICLAZIDE 40mg tablets |
| GP | f35y. | GLICLAZIDE 30mg m/r tablets |
| GP | f35z. | GLICLAZIDE 80mg tablets |
| GP | f36.. | GLIPIZIDE |
| GP | f361. | GLIPIZIDE 5mg tablets |
| GP | f362. | *GLIBENESE 5mg tablets |
| GP | f363. | *MINODIAB 2.5mg tablets |
| GP | f364. | MINODIAB 5mg tablets |
| GP | f36y. | *GLIPIZIDE 5mg tablets |
| GP | f36z. | GLIPIZIDE 2.5mg tablets |
| GP | f37.. | GLIQUIDONE |
| GP | f371. | *GLURENORM 30mg tablets |
| GP | f37z. | *GLIQUIDONE 30mg tablets |
| GP | f38.. | *GLYMIDINE |
| GP | f381. | *GONDAFON 500mg tablets |
| GP | f38z. | *GLYMIDINE 500mg tablets |
| GP | f39.. | TOLAZAMIDE |
| GP | f391. | TOLANASE 100mg tablets |
| GP | f392. | TOLANASE 250mg tablets |
| GP | f39y. | TOLAZAMIDE 100mg tablets |
| GP | f39z. | TOLAZAMIDE 250mg tablets |
| GP | f3a.. | TOLBUTAMIDE |
| GP | f3a1. | TOLBUTAMIDE 500mg tablets |
| GP | f3a2. | GLYCONON 500mg tablets |
| GP | f3a3. | *PRAMIDEX 500mg tablets |
| GP | f3a4. | *RASTINON 500mg tablets |
| GP | f3A.. | GLIMEPIRIDE |
| GP | f3A1. | GLIMEPIRIDE 2mg tablets |
| GP | f3A2. | AMARYL 2mg tablets |
| GP | f3A3. | GLIMEPIRIDE 1mg tablets |
| GP | f3A4. | GLIMEPIRIDE 3mg tablets |
| GP | f3A5. | GLIMEPIRIDE 4mg tablets |
| GP | f3A6. | AMARYL 1mg tablets |
| GP | f3A7. | AMARYL 3mg tablets |
| GP | f3A8. | AMARYL 4mg tablets |
| GP | f3A9. | NIDDARYL 1mg tablets |
| GP | f3AA. | NIDDARYL 2mg tablets |
| GP | f3AB. | NIDDARYL 3mg tablets |
| GP | f3AC. | NIDDARYL 4mg tablets |
| GP | f4... | BIGUANIDES |
| GP | f41.. | METFORMIN HYDROCHLORIDE |
| GP | f411. | GLUCOPHAGE 500mg tablets |
| GP | f412. | GLUCOPHAGE 850mg tablets |
| GP | f413. | *ORABET 500mg tablets |
| GP | f414. | *ORABET 850mg tablets |
| GP | f415. | *GLUCAMET-500 tablets |
| GP | f416. | *GLUCAMET-850 tablets |
| GP | f417. | GLUCOPHAGE SR 500mg m/r tablets |
| GP | f418. | METSOL 500mg/5mL oral solution |
| GP | f419. | GLUCOPHAGE SR 750mg m/r tablets |
| GP | f41A. | GLUCOPHAGE SR 1000mg m/r tablets |
| GP | f41B. | BOLAMYN SR 500mg m/r tablets |
| GP | f41C. | GLUCOPHAGE 500mg/sachet oral powder |
| GP | f41D. | GLUCOPHAGE 1000mg/sachet oral powder |
| GP | f41E. | METABET SR 500mg m/r tablets |
| GP | f41F. | METABET SR 1000mg m/r tablets |
| GP | f41G. | GLUCIENT SR 500mg m/r tablets |
| GP | f41s. | METFORMIN HYDROCHLORIDE 1000mg/sachet oral powder |
| GP | f41t. | METFORMIN HYDROCHLORIDE 500mg/sachet oral powder |
| GP | f41u. | METFORMIN HYDROCHLORIDE 1000mg m/r tablets |
| GP | f41v. | METFORMIN HYDROCHLORIDE 750mg m/r tablets |
| GP | f41w. | METFORMIN 500mg/5mL oral solution |
| GP | f41x. | METFORMIN HYDROCHLORIDE 500mg m/r tablets |
| GP | f41y. | METFORMIN HYDROCHLORIDE 500mg tablets |
| GP | f41z. | METFORMIN HYDROCHLORIDE 850mg tablets |
| GP | ft... | OTHER DRUGS USED IN DIABETES |
| GP | ft1.. | ACARBOSE |
| GP | ft11. | ACARBOSE 50mg tablets |
| GP | ft12. | ACARBOSE 100mg tablets |
| GP | ft13. | GLUCOBAY 50 tablets |
| GP | ft14. | GLUCOBAY 100 tablets |
| GP | ft2.. | TROGLITAZONE |
| GP | ft21. | *TROGLITAZONE 200mg tablets |
| GP | ft22. | *TROGLITAZONE 300mg tablets |
| GP | ft23. | *TROGLITAZONE 400mg tablets |
| GP | ft24. | *ROMOZIN 200mg tablets |
| GP | ft25. | *ROMOZIN 300mg tablets |
| GP | ft26. | *ROMOZIN 400mg tablets |
| GP | ft3.. | REPAGLINIDE |
| GP | ft31. | REPAGLINIDE 0.5mg tablets |
| GP | ft32. | REPAGLINIDE 1mg tablets |
| GP | ft33. | REPAGLINIDE 2mg tablets |
| GP | ft34. | *NOVONORM 0.5mg tablets |
| GP | ft35. | *NOVONORM 1mg tablets |
| GP | ft36. | *NOVONORM 2mg tablets |
| GP | ft37. | PRANDIN 500micrograms tablets |
| GP | ft38. | PRANDIN 1mg tablets |
| GP | ft39. | PRANDIN 2mg tablets |
| GP | ft4.. | ROSIGLITAZONE |
| GP | ft41. | *AVANDIA 4mg tablets |
| GP | ft42. | *AVANDIA 8mg tablets |
| GP | ft43. | *AVANDAMET 1mg / 500mg tablets |
| GP | ft44. | *AVANDAMET 2mg / 500mg tablets |
| GP | ft45. | AVANDAMET 2mg / 1000mg tablets |
| GP | ft46. | AVANDAMET 4mg / 1000mg tablets |
| GP | ft4u. | ROSIGLITAZONE 2mg / METFORMIN 1000mg tablets |
| GP | ft4v. | ROSIGLITAZONE 4mg / METFORMIN 1000mg tablets |
| GP | ft4w. | ROSIGLITAZONE 2mg / METFORMIN 500mg tablets |
| GP | ft4x. | ROSIGLITAZONE 1mg / METFORMIN 500mg tablets |
| GP | ft4y. | *ROSIGLITAZONE 8mg tablets |
| GP | ft4z. | *ROSIGLITAZONE 4mg tablets |
| GP | ft5.. | PIOGLITAZONE |
| GP | ft51. | ACTOS 15mg tablets |
| GP | ft52. | ACTOS 30mg tablets |
| GP | ft53. | ACTOS 45mg tablets |
| GP | ft5x. | PIOGLITAZONE 45mg tablets |
| GP | ft5y. | PIOGLITAZONE 30mg tablets |
| GP | ft5z. | PIOGLITAZONE 15mg tablets |
| GP | ft6.. | NATEGLINIDE |
| GP | ft61. | STARLIX 60mg tablets |
| GP | ft62. | STARLIX 120mg tablets |
| GP | ft63. | STARLIX 180mg tablets |
| GP | ft6x. | NATEGLINIDE 180mg tablets |
| GP | ft6y. | NATEGLINIDE 120mg tablets |
| GP | ft6z. | NATEGLINIDE 60mg tablets |
| GP | ft7.. | METFORMIN + PIOGLITAZONE |
| GP | ft71. | COMPETACT 15mg/850mg tablets |
| GP | ft7z. | METFORMIN 850mg/PIOGLITAZONE 15mg tablets |
| GP | ft8.. | SITAGLIPTIN |
| GP | ft81. | JANUVIA 100mg tablets |
| GP | ft8z. | SITAGLIPTIN 100mg tablets |
| GP | ft9.. | EXENATIDE |
| GP | ft91. | BYETTA 5micrograms/0.02mL injection prefilled pen |
| GP | ft92. | BYETTA 10micrograms/0.04mL injection prefilled pen |
| GP | ft93. | BYDUREON 2mg powder and solvent for suspension for injection |
| GP | ft9x. | EXENATIDE 2mg powder+solvent for suspension for injection |
| GP | ft9y. | EXENATIDE 10micrograms/0.04mL injection prefilled pen |
| GP | ft9z. | EXENATIDE 5micrograms/0.02mL injection prefilled pen |
| GP | fta.. | VILDAGLIPTIN |
| GP | fta1. | GALVUS 50mg tablets |
| GP | ftaZ. | VILDAGLIPTIN 50mg tablets |
| GP | ftb.. | METFORMIN + VILDAGLIPTIN |
| GP | ftb1. | EUCREAS 50mg/850mg tablets |
| GP | ftb2. | EUCREAS 50mg/1000mg tablets |
| GP | ftby. | VILDAGLIPTIN/METFORMIN 50mg/1000mg tablets |
| GP | ftbz. | VILDAGLIPTIN/METFORMIN 50mg/850mg tablets |
| GP | ftc.. | LIRAGLUTIDE |
| GP | ftc1. | VICTOZA 6mg/mL solution for injection prefilled pen 3mL |
| GP | ftc2. | LIRAGLUTIDE 6mg/mL solution for injection prefilled pen |
| GP | ftd.. | SAXAGLIPTIN |
| GP | ftd1. | ONGLYZA 5mg tablets |
| GP | ftd2. | ONGLYZA 2.5mg tablets |
| GP | ftdy. | SAXAGLIPTIN 2.5mg tablets |
| GP | ftdz. | SAXAGLIPTIN 5mg tablets |
| GP | fte.. | METFORMIN + SITAGLIPTIN |
| GP | fte1. | JANUMET 50mg/1000mg tablets |
| GP | ftez. | SITAGLIPTIN/METFORMIN HYDROCHLORIDE 50mg/1000mg tablets |
| GP | ftf.. | LINAGLIPTIN |
| GP | ftf1. | TRAJENTA 5mg tablets |
| GP | ftf2. | LINAGLIPTIN 5mg tablets |

|  | **CODE** | **DESCRIPTION** |
| --- | --- | --- |
| HOSPITAL ADMISSION | E10% | Type 1 Diabetes |
| HOSPITAL ADMISSION | E11% | Type 2 Diabetes |
| HOSPITAL ADMISSION | E13% | Other specified diabetes mellitus |
| HOSPITAL ADMISSION | E14% | Unspecified diabetes mellitus |

Asthma:

| GP | 1737 | Wheezing | |  |
| --- | --- | --- | --- | --- |
| GP | 173A. | Exercise induced asthma | | |
| GP | 173B. | Nocturnal cough / wheeze | | |
| GP | 173e. | Viral wheeze | |  |
| GP | 178.. | Asthma trigger | |  |
| GP | 1780 | Aspirin induced asthma | | |
| GP | 1781 | Asthma trigger - pollen | | |
| GP | 1782 | Asthma trigger - tobacco smoke | | |
| GP | 1783 | Asthma trigger - warm air | | |
| GP | 1784 | Asthma trigger - emotion | | |
| GP | 1785 | Asthma trigger - damp | | |
| GP | 1786 | Asthma trigger - animals | | |
| GP | 1787 | Asthma trigger - seasonal | | |
| GP | 1788 | Asthma trigger - cold air | | |
| GP | 1789 | Asthma trigger - respiratory infection | | |
| GP | 178A. | Asthma trigger - airborne dust | | |
| GP | 178B. | Asthma trigger - exercise | | |
| GP | 1J70. | Suspected asthma | |  |
| GP | 1O2.. | Asthma confirmed | |  |
| GP | 2326 | O/E - expiratory wheeze | | |
| GP | 23D2. | O/E - rhonchi present | | |
| GP | 6635 | Increasing exercise wheeze | | |
| GP | 663d. | Emergency asthma admission since last appointment | | |
| GP | 663e. | Asthma restricts exercise | | |
| GP | 6.63E+02 | Asthma sometimes restricts exercise | | |
| GP | 6.63E+03 | Asthma severely restricts exercise | | |
| GP | 663f. | Asthma never restricts exercise | | |
| GP | 663j. | Asthma - currently active | | |
| GP | 663J. | Airways obstruction reversible | | |
| GP | 663K. | Airways obstructn irreversible | | |
| GP | 663m. | Asthma accident and emergency attendance since last visit | | |
| GP | 663n. | Asthma treatment compliance satisfactory | | |
| GP | 663N. | Asthma disturbing sleep | | |
| GP | 663N0 | Asthma causing night waking | | |
| GP | 663N1 | Asthma disturbs sleep weekly | | |
| GP | 663N2 | Asthma disturbs sleep frequently | | |
| GP | 663O. | Asthma not disturbing sleep | | |
| GP | 663O0 | Asthma never disturbs sleep | | |
| GP | 663p. | Asthma treatment compliance unsatisfactory | | |
| GP | 663P. | Asthma limiting activities | | |
| GP | 663P0 | Asthma limits activities 1 to 2 times per month | | |
| GP | 663P1 | Asthma limits activities 1 to 2 times per week | | |
| GP | 663P2 | Asthma limits activities most days | | |
| GP | 663q. | Asthma daytime symptoms | | |
| GP | 663Q. | Asthma not limiting activities | | |
| GP | 663r. | Asthma causes night symptoms 1 to 2 times per month | | |
| GP | 663s. | Asthma never causes daytime symptoms | | |
| GP | 663t. | Asthma causes daytime symptoms 1 to 2 times per month | | |
| GP | 663u. | Asthma causes daytime symptoms 1 to 2 times per week | | |
| GP | 663v. | Asthma causes daytime symptoms most days | | |
| GP | 663V. | Asthma severity | |  |
| GP | 663V0 | Occasional asthma | |  |
| GP | 663V1 | Mild asthma | |  |
| GP | 663V2 | Moderate asthma | |  |
| GP | 663V3 | Severe asthma | |  |
| GP | 663w. | Asthma limits walking up hills or stairs | | |
| GP | 663x. | Asthma limits walking on the flat | | |
| GP | 663y. | Number of asthma exacerbations in past year | | |
| GP | 66Y9. | Step up change in asthma management plan | | |
| GP | 66YC. | Absent from work or school due to asthma | | |
| GP | 66YK. | Asthma follow-up | |  |
| GP | 66YP. | Asthma night-time symptoms | | |
| GP | 66Yq. | Asthma causes night time symptoms 1 to 2 times per week | | |
| GP | 66Yr. | Asthma causes symptoms most nights | | |
| GP | 8793 | Asthma control step 0 | | |
| GP | 8794 | Asthma control step 1 | | |
| GP | 8795 | Asthma control step 2 | | |
| GP | 8796 | Asthma control step 3 | | |
| GP | 8797 | Asthma control step 4 | | |
| GP | 8798 | Asthma control step 5 | | |
| GP | 8H2P. | Emergency admission, asthma | | |
| GP | H302. | Wheezy bronchitis | |  |
| GP | H3120 | Chronic asthmatic bronchitis | | |
| GP | H33.. | Asthma |  |  |
| GP | H330. | Extrinsic (atopic) asthma | | |
| GP | H3300 | Extrinsic asthma without status asthmaticus | | |
| GP | H3301 | Extrinsic asthma with status asthmaticus | | |
| GP | H330z | Extrinsic asthma NOS | | |
| GP | H331. | Intrinsic asthma | |  |
| GP | H3310 | Intrinsic asthma without status asthmaticus | | |
| GP | H3311 | Intrinsic asthma with status asthmaticus | | |
| GP | H331z | Intrinsic asthma NOS | | |
| GP | H332. | Mixed asthma | |  |
| GP | H333. | Acute exacerbation of asthma | | |
| GP | H334. | Brittle asthma | |  |
| GP | H335. | Chronic asthma with fixed airflow obstruction | | |
| GP | H33z. | Asthma unspecified | |  |
| GP | H33z0 | Status asthmaticus NOS | | |
| GP | H33z1 | Asthma attack | |  |
| GP | H33z2 | Late-onset asthma | |  |
| GP | H33zz | Asthma NOS | |  |
| GP | H35y7 | Wood asthma | |  |
| GP | H47y0 | Detergent asthma | |  |
| GP | H5y16 | Bronchospasm | |  |
| GP | R0609 | [D]Wheezing | |  |
| GP | R060E | [D]Mild wheeze | |  |
| GP | R060F | [D]Moderate wheeze | | |
| GP | R060G | [D]Severe wheeze | |  |
| GP | R060H | [D]Very severe wheeze | | |
| HOSPITAL ADMISSION | J45.8 | Mixed asthma | | |
| HOSPITAL ADMISSION | J45.8 | Mixed asthma | | |
| HOSPITAL ADMISSION | J45.9 | Asthma, unspecified | | |
| HOSPITAL ADMISSION | J45.9 | Asthma, unspecified | | |
| HOSPITAL ADMISSION | J45 | Asthma | | |
| HOSPITAL ADMISSION | J45 | Asthma | | |
| HOSPITAL ADMISSION | J45.1 | Nonallergic asthma | | |
| HOSPITAL ADMISSION | J45.1 | Nonallergic asthma | | |
| HOSPITAL ADMISSION | J45.0 | Predominantly allergic asthma | | |
| HOSPITAL ADMISSION | J45.0 | Predominantly allergic asthma | | |

ADHD

| GP | 6A61. | Attention deficit hyperactivity disorder annual review |
| --- | --- | --- |
| GP | 9Ol8. | Attention deficit hyperactivity disorder monitoring invitation first letter |
| GP | 9Ol9. | Attention deficit hyperactivity disorder monitoring invitation second letter |
| GP | 9OlA. | Attention deficit hyperactivity disorder monitoring invitation third letter |
| GP | E2E01 | Attention deficit with hyperactivity |
