# Supplementary material for: Characteristics of Children Prescribed Antipsychotics: Analysis of Routinely Collected Data
Source: J Child Adolesc Psychopharmacol. 2018 Apr 1;28(3):180–91. doi: 10.1089/cap.2017.0003 (PMC5905863; doi:10.1089/cap.2017.0003)
Supplement: Supplemental data [file Supp_Data.zip › Supp_Data.pdf]

Supplementary Data

Overlap Between General Practice Records and Educational Records

A total of 32,526 children were identified who had a code for learning difficulty in at least one database. Of these, 15.9% had a general practice (GP) record, but no education records. This may be because the child was too young to have sat national tests or was in a private/independent school. 31.6% had a learning disability as identified by the GP records, but the education record did not mention the learning disability. 42.3% had a learning disability in both GP and education records, and 10.1% had a learning difficulty as recorded in the education data set, but there were no GP records for this child (GP not submitting data to the Secure Anonymised Information Linkage [SAIL] databank).
